# Structural evidence that a lipid plug controls K_2P_6.1(TWIK-2) function

**DOI:** 10.1038/s41467-026-74535-6

**Published:** 2026-06-16

**Authors:** Abhisek Mondal, Sangeeta Niranjan, Daniel L. Minor

**Affiliations:** 1 Cardiovascular Research Institute, University of California, San Francisco, CA USA; 2 Department of Biochemistry and Biophysics, University of California, San Francisco, CA USA; 3 Department of Cellular and Molecular Pharmacology, University of California, San Francisco, CA USA; 4 California Institute for Quantitative Biomedical Research, University of California, San Francisco, CA USA; 5 Kavli Institute for Fundamental Neuroscience, University of California, San Francisco, CA USA; 6 Molecular Biophysics and Integrated Bio-imaging Division Lawrence Berkeley National Laboratory, Berkeley, CA USA

**Keywords:** Molecular biophysics, Cryoelectron microscopy, Potassium channels

## Abstract

Lipids are integral to ion channel function yet delineating mechanisms by which they affect function remains challenging. Within the K_2P_ family of leak potassium channels, observation of tubular densities interpreted as alkyl chains occupying lateral fenestrations linking the pore and bilayer raised the possibility that lipid access from the bilayer acts as a regulatory mechanism. Here, we present cryo-electron microscopy (cryo-EM) structures of the human leak potassium channel K_2P_6.1 (TWIK2) and mutants in nanodisc and detergent environments that reveal an unusual conformation in the first selectivity filter (SF1) and a pair of two-chain lipids within the channel cavity (denoted the ‘lipid plug’). The chains of each plug lipid occupy separate binding sites that laterally extend to the bilayer from the channel cavity. One, the upper tail, matches the previously identified alkyl chain binding site. The second, the lower tail, occupies a fenestration common with K_2P_1.1 (TWIK1). Together, they demonstrate a two-tailed means to coordinate each plug lipid that offers a reinterpretation of previous observations. Structures of a K_2P_6.1 (TWIK2) mutant that directs the channel to the plasma membrane and an R257A mutant that increases function yield plugged and unplugged forms. Notably, the R257A plugged form shows a change in lipid plug position, indicating a key role for this residue in lipid binding. Molecular dynamics simulations support the stability of the lipid plug and highlight the role of the Arg257 site in plug coordination. Together, our data suggest a model in which occupation of the central cavity by the lipid plug serves as a mechanism to render the TWIK channels inactive and points to the importance of lipid plug removal to create an ion permeable pore. Such a mechanism could provide a potent way for limiting the leak function of K_2P_s based on cellular location or other contextual factors.

## Abstract (web summary)

Here the authors use cryo-EM to reveal a lipid plug that blocks the K2P6.1(TWIK-2) leak potassium channel pore. This plug is removed when the channel is sent to the plasma membrane suggesting a structural mechanism for silencing channel function.

## Introduction

K_2P_ potassium channels produce leak currents that are important for controlling resting membrane potential in diverse cell types^1–3^. Fifteen human K_2P_ channel subunits comprise six subfamilies (TWIK, TREK, THIK, TASK, TALK, and TRESK) that each respond to diverse sets of physiological cues including physical force, lipid modulation, and various signaling cascades^4^. Structural studies of members from the TWIK^5,6^, TREK^7–9^, TALK^10^, TASK^11^, and THIK^12,13^ subfamilies reveal a shared architecture^4^ in which the two pore domains (PD1 and PD2) of an individual K_2P_s subunit form a pseudo-tetrameric pore upon subunit dimerization. In contrast to other types of potassium channels, the selectivity filter (SF) “C-type” gate acts as the principal site of K_2P_ modulation for most K_2P_s^4,7,14–18^. Despite their relatively small size (~60–70 kDa), K_2P_s contain a wealth of binding sites for various classes of lipid and small molecule modulators whose roles in channel gating remain imperfectly understood^4^. In particular, the potential role of lipid access through a lateral fenestration below the selectivity filter has attracted much attention^6,19^, but whether this represents a mechanism for direct block of the pore remains a point of controversy, as simulations indicate that even though lipid tails can enter, they do not block the pore^20,21^.

K_2P_6.1 (TWIK2)^22–24^ belongs to the TWIK subfamily comprising K_2P_1.1 (TWIK1), K_2P_6.1 (TWIK2), and K_2P_7.1^2^ and is found in the gastrointestinal tract, vasculature, and immune system^22^ where it is linked to vascular^25^ and pulmonary^26^ hypertension and sepsis responses^27^. K_2P_6.1 (TWIK2) and K_2P_1.1 (TWIK1) are notable for their endolysosomal localization^28^. Functional and cell biology studies indicate that in K_2P_6.1 (TWIK2), this intracellular distribution originates from internalization signals that favor this location and largely prevent plasma membrane expression^29^. Interestingly, in macrophages, transit of endosomally sequestered K_2P_6.1 (TWIK2) to the plasma membrane following a signal initiated by extracellular ATP has been implicated in the process underlying NLRP3 (NOD, LRR and pyrin domain-containing protein 3) inflammasome activation^27,30–32^. This K_2P_6.1 (TWIK2) function parallels that described for the THIK subfamily in NLRP3 activation in microglia and interleukin 1β (IL−1β) release^33–35^, highlighting the importance of the link between K_2P_ cellular location and function.

Here, we report structural studies of human K_2P_6.1 (TWIK2) and mutants in lipid nanodisc and detergent environments. The data reveal a channel architecture that largely resembles K_2P_1.1 (TWIK1) having three notable features: an extra helix in the extracellular cap domain (the “ear helix”), an unusual conformation of a key residue in the first selectivity filter (SF1), and block by pair of intracellular lipids that engage the channel through a set of two-tailed interactions with lateral fenestrations linking the pore and bilayer. Elements from this last feature occupy the site that has been identified in K_2P_1.1 (TWIK1)^5,6^ and other K_2P_s^9,19,36^ as a binding site for a single alkyl chain^5,9,19,36^ or detergent^5,37^. Structures of a previously characterized K_2P_6.1 (TWIK2) mutant that increases the plasma membrane localization and activity^29^ yield plugged and unplugged forms that highlight the importance of lipid removal for channel function. Moreover, the structure of the R257A mutant that enhances function and affects a key lipid coordinating residue reveals a descent of the lipid plug towards the intracellular opening, highlighting the important role of Arg257 in lipid coordination. This key role for the Arg257 site is further supported by molecular dynamics studies that reveal dynamic interactions between Arg257 and the lipid plug. The proposal that the lipid plug forms a physical barrier that prevents ion passage and that this plug can be destabilized by mutations that enhance channel function suggests a working model in which the function of the TWIK subfamily depends on lipid plug removal.

## Results

### Human K_2P_6.1(TWIK-2) structure reveals an unusual selectivity filter conformation and a lipid plug

**Fig. 1:**
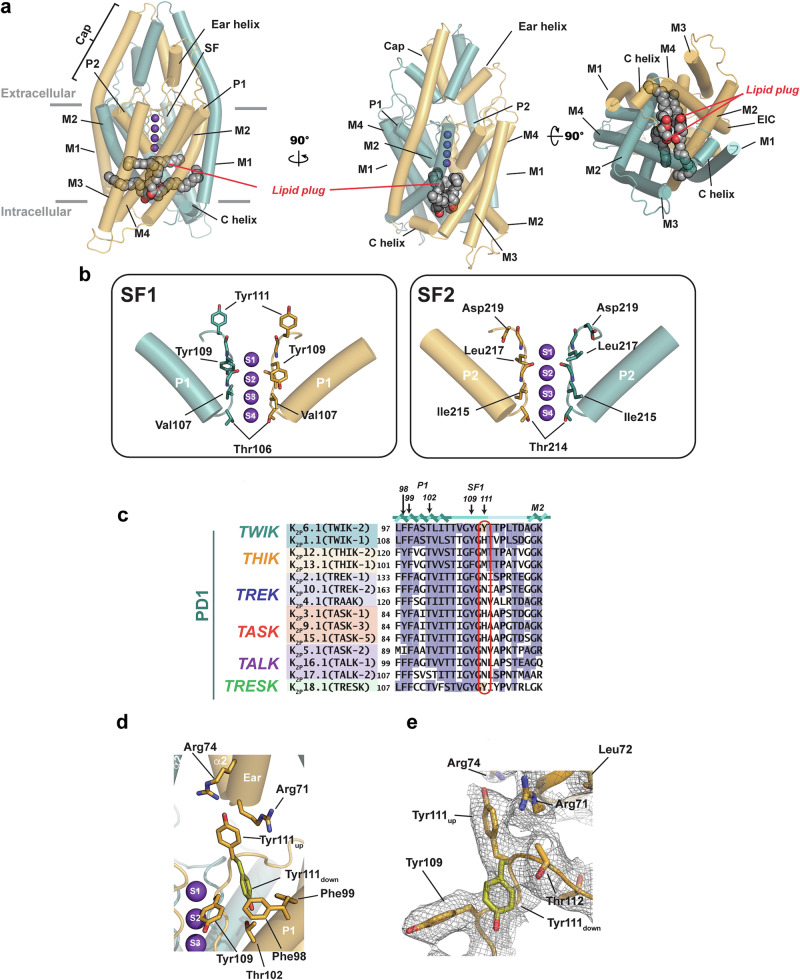
K_2P_6.1(TWIK-2) structural features. **a** K_2P_6.1(TWIK-2) cartoon diagram (deep teal and bright orange) showing side (left and center) and intracellular (right) views. Lipid plug (grey) is shown in space filling. Grey bars indicate membrane. Potassium ions are shown as purple spheres. **b** Comparison of K_2P_6.1(TWIK-2) SF1 (left) and SF2 (right) filter conformations. Select matching positions are shown as sticks. **c** Sequence comparison P1, SF1, and initial segment of M2 from PD1 for K_2P_6.1 (TWIK-2) with other K_2P_s. Red oval indicates Tyr111 site. Labels indicate residues that interact with Tyr111 in the ‘down’ state. Conservation is indicated in blue. Tyr111 and similar residues are shaded red. Sequences are for human: K_2P_6.1(TWIK-2) (GENBANK 4758624); K_2P_ 1.1(TWIK-1) (GENBANK 4504847); K_2P_13.1(THIK-1) (GENBANK 16306555); K_2P_12.1(THIK-2) (GENBANK 11545761); K_2P_2.1(TREK-1) (GENBANK 14589851); K_2P_10.1(TREK-2) (GENBANK 20143944); K_2P_4.1(TRAAK) (GENBANK 15718767); K_2P_3.1(TASK-1) (GENBANK 4504849); K_2P_9.1 (TASK-3) (GENBANK 542133161); K_2P_15.1(TASK-5) (GENBANK 333440483); K_2P_5.1(TASK-2) (GENBANK 333440483); K_2P_16.1(TALK-1) (GENBANK 14149764); K_2P_17.1(TALK-2) (GENBANK 17025230); and K_2P_18.1(TRESK) (GENBANK 32469495). **d** K_2P_13.1(THIK-1) Tyr111 environment and interactions. ‘up’ (bright orange) and ‘down’ (olive) Tyr111 conformations are indicated. **e** Cryo-EM density for the region including Tyr111 (σ = 2.5).

Expression and purification of full-length human K_2P_6.1(TWIK-2) (denoted “TWIK2”) from HEK293 cells yielded a sample suitable for cryo-EM studies (Fig. S1a) that we used for structural characterization in MSP1E1^38^ lipid nanodiscs under high potassium (200 mM KCl) conditions (Figs. S1 and S2, and Table 1). The structure (TWIK2:ND) has an overall resolution of 3.2 Å, with the best-defined parts reaching 2.2 Å (Figs. 1a and S2c, and Table 1). TWIK2 has the canonical architecture found throughout the K_2P_ family^4^ (Fig. S3) and is very similar to its related TWIK subfamily member, K_2P_1.1 (TWIK-1)^5,6^ (RMSD_Cα_ = 0.817 Å 3UKM^5^, 1.836 Å 7SK0^6^, 1.338 Å 7SK1^6^). The two PDs from each subunit are arranged around the central axis of the channel with the M2 and M4 helices lining the pore and the M1 and M3 helices located on the periphery. The M1 helix is domain swapped between the two subunits and connects to the extracellular Cap domain as in other K_2P_s^4^. There is a branched aqueous pathway, the extracellular ion pathway (EIP), between the Cap and extracellular face of the pore that connects the external mouth of the channel selectivity filter (SF) with the extracellular space^4,5,36^. Different from other K_2P_s, the TWIK2 Cap domain has a third helix (denoted the “ear helix”) that connects the Cap to the loop leading to the P1 helix (Figs. 1b, and S4a, b). Ear helix residues Gly73, Val76, Leu77, and Asn79 from one subunit pack against the α2 helix from its own Cap and the α1 and α2 Cap helices from its dimeric partner. These positions are largely conserved among vertebrate TWIK2s (Fig. S5) and with the exception of a hydrogen bond pair (Glu41-Asn79) are conserved in TWIK1 (Fig. S4b). By contrast, these sites are poorly conserved in other K_2P_ classes (Fig. S4a, b), suggesting that the Ear helix is a feature of the TWIK subfamily. Evaluation of surface electrostatics highlights the electronegative character of the extracellular EIP, a prominent positively charged surface on the intracellular M2-M3 loop, and a relatively hydrophobic inner cavity bounded by the selectivity filter and Arg257 (Fig. S6).

**Table 1:** Cryo-EM data collection, refinement and validation statistics

	TWIK2:ND	TWIK2:Det	TWIK2_RM_ :Det		TWIK2_RM_ R257A:Det		
	(EMD-70828) (PDB:9OTA)	(EMD-70837) (PDB:9OTK)	(EMD-70885) (PDB:9OV0)	(EMD-70894) (PDB:9OV9)	(EMD-70898) (PDB:9OVD)		(EMD-70899) (PDB:9OVE)
**Data collection and processing**							
Magnification	105,000	105,000	105,000		105,000		
Voltage (kV)	300	300	300		300		
Total dose (e–/Å^2^)	47.7	47.7	47.7		60		
Exposure time (s)	2	2	2		2.8		
Defocus range (μm)	–1.0 to –2.2	–1.0 to –2.2	–1.0 to –2.2		–1.0 to –2.5		
Pixel size (Å)	0.82	0.82	0.82		0.86		
Symmetry imposed	C2	C2	C2		C2		
Number of Movies	10,315	13,561	11,690		24,106		
Initial particle number	6,918,264	21,209,849	12,520,054		14,584,764		
			*plugged*	*unplugged*	*plugged*	*unplugged*	
Final particle number	164,658	174,536	112,432	112,195	134,257	187,094	
Map resolution (Å) FSC threshold (0.143)	3.2 Å	3.7Å	3.9Å	3.9Å	3.9Å	4.1Å	
Map resolution range (Å)	2.2–5.5Å	2.2–5.5Å	2.4–6.1Å	2.5–6.0Å	2.2–5.6Å	2.3–6.3Å	
**Refinement**							
Initial model used (PDB code)	8UE2						
Model resolution (Å) FSC threshold (0.143)	3.2Å	3.7Å	3.9Å	3.9Å	3.9Å	4.1Å	
Model resolution range (Å)	3.1–3.4Å	3.6–3.9Å	3.7–6.1Å	3.5–6.2Å	3.4–4.5Å	3.8–4.2Å	
Map sharpening *B* factor (Å^2^)	175.6	227	252.1	198.4	229.3	210.4	
Model composition							
Non-hydrogen atoms	4256	4210	4059	4055	4128	4039	
Protein residues	534	544	532	532	532	531	
Ligands	8	6	5	3	6	3	
*B* factors (Å^2^)							
Protein	40.3	88.7	110.1	201.5	48.9	219.4	
Ligand	25.2	74.6	59.1	35.4	27.2	161.1	
R.m.s. deviations							
Bond lengths (Å)	0.003	0.003	0.004	0.004	0.003	0.002	
Bond angles (°)	0.48	0.586	0.849	0.707	0.570	0.561	
Validation							
MolProbity score	2.0	1.9	1.9	2.3	2.1	2.0	
Clashscore	4.5	5.7	9.7	10.0	6.3	6.6	
Rotamer outliers (%)	4.3	2.3	0.7	2.6	3.1	3.4	
Ramachandran plot							
Favored (%)	95.2	95.5	94.8	93.9	93.7	96.8	
Allowed (%)	4.7	4.2	4.9	5.9	6.1	3.0	
Outliers (%)	0.0	0.1	0.1	0.1	0.1	0.1	

Consistent with structure solution in high potassium conditions, the TWIK2 selectivity filter shows density for ions at the S1–S4 positions (Figs. 1b, e and S2b). The selectivity filters of K_2P_ differ from some canonical features seen in other potassium channels. Notably, diverse amino acids occupy the last position of SF1 potassium channel signature selectivity filter sequence “TxTTxGYGD”^4,39^ (Fig. 1c), and contrast the SF2 sequence where this site is strongly conserved as an aspartic acid (Fig. S7a) as found in homomeric potassium channels^40^. Despite the SF1 sequence variation, in most K_2P_ structures^4^ both SFs adopt essentially the same local conformations in which the sidechain of the residue following the last SF glycine interacts with a network of residues behind the selectivity filter (Fig. S7b), similar to homomeric potassium channels^41^. We also find a lipid in the outer leaflet at the P2/M2 interface near Phe201. SF1 in TWIK2 stands out by having a large aromatic residue, Tyr111, following the selectivity filter “GYG” sequence (Fig. 1c). The structure shows that rather than adopting the canonical “down” conformation seen in SF1 from K_2P_1.1(TWIK1)^5,6^ and K_2P_2.1(TREK-1)^7^ exemplars (Fig. S7b, c) and the equivalent SF2 position (Figs. 1c and S7b, d), Tyr111 occupies an ‘up’ conformation in which it interacts with Arg71 and Arg74 from the ear helix base (Fig. 1b–d), similar to the observations from recent structures of TWIK2 in detergent^42,43^. Inspection of the TWIK2:ND density using focused refinement showed evidence for a second Tyr111 conformation corresponding to the “down” state (Fig. 1d, e) in which Tyr111 interacts with a pocket formed by P1 helix residues Phe98, Phe99, and Thr102 and SF1 residue Tyr109 (Fig. 1d). The “up” and ‘down’ conformations are essentially Tyr111 rotamer changes that do not require major changes in the surrounding SF structure. Notably, a similar “up” rotamer of the corresponding residue in K_2P_3.1 (TASK-1), His98, was observed in a pH 7.5 structure (Fig. S7e)^44^, contrasting a “down” conformation seen at higher pH^11^. Together, these observations show that the SF1 “up”/“down” rotamer change can occur in different K_2P_ subfamilies. The additional conformational changes observed for the equivalent SF1 position in K_2P_1.1 (TWIK1)^6^ (Fig. S7f) and K_2P_2.1(TREK-1)^17^ under conditions associated with inactivation of the channel C-type SF gate highlight the importance of structural changes at this site for K_2P_ function.

**Fig. 2:**
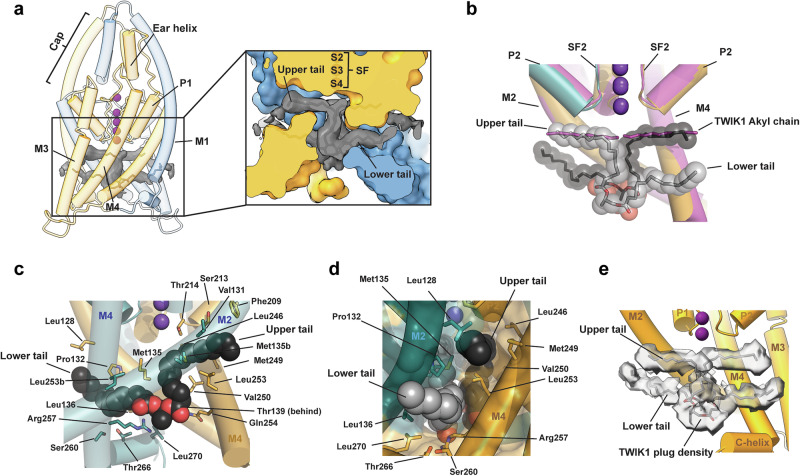
K_2P_6.1(TWIK-2) lipid plug blocks the central cavity. **a** K_2P_6.1(TWIK-2) cartoon diagram (deep teal and bright orange). Cryo-EM density for lipid plug (grey) is shown (σ =3.0). Inset shows slice through space-filling model of the channel, lipid plug cryo-EM density and stick representation of lipid plug. **b** Superposition of K_2P_6.1(TWIK2) (deep teal and bright orange) and K_2P_1.1(TWIK1) (magenta) (PDB:3UKM)^5^. K_2P_6.1(TWIK2) lipid plug (grey and black) is shown as sticks and semi-transparent space filling. Upper and lower tails for one lipid are indicated. TWIK1 Alkyl chain^5^ is shown as sticks (magenta). **c** K_2P_6.1(TWIK2) lipid plug interactions. One lipid plug chain is shown (black, space filling). Contacting residues from the two K_2P_6.1(TWIK2) subunits (deep teal and bright orange) are shown as sticks. **d** View from the center of the bilayer towards the upper and lower tail binding sites. Lipid plug chains are grey and black and shown in space filling. Interacting residues are shown as sticks. **e** Lipid plug density (1.9σ) from K_2P_1.1 (TWIK1) at pH5.5 (EMD-25169)^6^. Model shows a single K_2P_6.1(TWIK2) subunit and lipid plug (grey).

The TWIK2 structure also revealed a second notable feature, a pair of branched densities that correspond to the tails of two lipids stacked face to face that obstruct the intracellular aqueous cavity (Figs. 1a and 2a, and S2a–d). The two tails of each lipid fill the hydrophobic inner cavity above Arg257 and occupy two fenestrations at the interface between P2 and M4 from one subunit and M2 from the other that lead to the membrane bilayer. The upper tail site formed by P2, M2 and M4 is lined by Leu128, Pro132, Met135, Leu246, Met249, Val250, and Leu253. This site corresponds to a site occupied by a single alkyl chain in TWIK1^5^ (Figs. 2b–d and S8a) and in recent TWIK2 structures in detergent^42,43^. The lipid lower tail site is formed by M2 and M4 and is lined by Leu136, Leu253, Arg257, Ser260, Thr266, and Leu270 (Figs. 2b–d and S8a). Although it is not well defined by the density, the overall structure of the channel places Arg257, a residue conserved in vertebrate TWIK2 (Fig. S5), at the level of the lipid glycerol backbone (Figs. 2c and S8a). Except for Arg257, the lipid plug contacting residues are conserved between TWIK1 and TWIK2 (Fig. S8b, c).

The unexpected presence of the lipids in the channel cavity prompted us to ask whether these cavity-filling molecules originated from the nanodisc reconstitution step or were present from an earlier step in channel purification. Hence, we determined the structure of TWIK2 in detergent at 3.7 Å (local resolution to 2.2 Å) (TWIK2:Det) (Figs. S9 and S10, and Table 1). TWIK2:Det was essentially identical to TWIK2:ND (RMSD_Cα_ = 0.44 Å), having the same features, including an “up” Tyr111 (Fig. S10a) and ions at sites S1-S4 of the selectivity filter. Importantly, the structure shows density corresponding to the two lipid molecules forming the lipid plug (Fig. S10a), demonstrating that these entities do not originate from the lipids used for the nanodisc reconstitution but come from the cells used to produce the protein sample.

The TWIK1 structure^5^ also has a fenestration below the upper tail site^21^. Comparison with the lipid plug from our TWIK2 structure shows that this cavity matches the position of the plug lower tail (Fig. S8d), suggesting that TWIK1 could also accommodate lipids using the TWIK2 two-tailed lipid plug binding pose. Prompted by this structural similarly, we inspected previously reported cryo-EM densities for TWIK1^6^ for evidence of the lipid plug. Remarkably, the density map of TWIK1 at pH 5.5, a structure thought to represent an inactivated channel, shows a bifurcated density in the channel central cavity that is matched by the TWIK2 lipid plug (Fig. 2e). The map for the TWIK1 pH 7.4 structure, in which the upper site was proposed to be empty of the alkyl chain shows a weaker version of a similar density (Fig. S8e). Thus, the lipid plug binding mode appears compatible among varied members of the TWIK subfamily. The conservation of the residues lining both lipid-occupied cavities, particularly in M4, does not extend to other K_2P_ family members (Fig. S8c). Hence, the architecture that enables this bifurcated type of lipid binding in the channel central cavity appears to be a special feature of the TWIK subfamily. Together, these results highlight the distinct binding mode of the TWIK2 plug lipid in which each lipid is anchored in the central cavity by occupying the upper and lower tail sites on opposite sides of the channel.

### A trafficking mutation yields unplugged channels

**Fig. 3:**
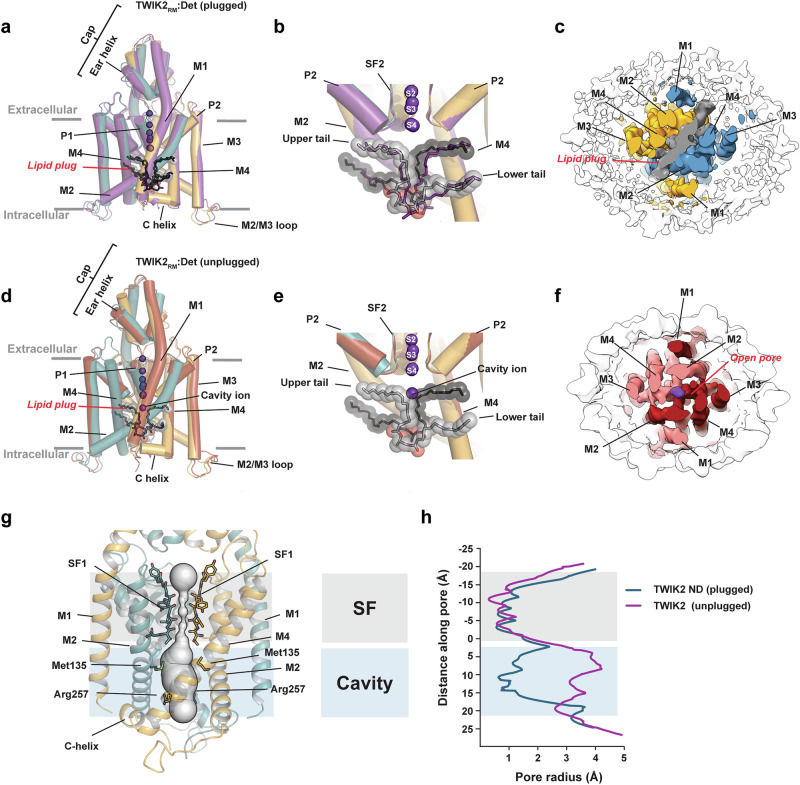
Comparison of plugged and unplugged K_2P_6.1 (TWIK2) structures. **a** Superposition of TWIK2:ND (deep teal and bright orange) and TWIK2_RM_:Det (plugged) (deep purple) structures. **b** Close up view of the central cavity from ‘a’. Lipid plugs from TWIK2:ND and TWIK2_RM_:Det (plugged) are shown as spheres (grey) and sticks (deep purple), respectively. **c** Intracellular view of a slice through the TWIK2_RM_:Det (plugged) density. Subunits are deep teal and bright orange, lipid plug density is grey. **d** Superposition of TWIK2:ND (deep teal and bright orange) and TWIK2_RM_:Det (unplugged) (firebrick) structures. **e** Close up view of the central cavity from ‘d’. Lipid plug from TWIK2:ND is shown as spheres (grey). TWIK2_RM_:Det (unplugged) cavity ion is labeled. **f** Intracellular view of a slice through the TWIK2_RM_:Det (unplugged) density. Subunits are firebrick and light red. **g** Pore profile of K_2P_6.1(TWIK2) (deep teal and bright orange) calculated using HOLE^88^. Selectivity filter and key cavity positions are shown as sticks. **h** K_2P_6.1(TWIK2) pore profiles for plugged (blue) and unplugged (magenta) forms. Selectivity filter (SF) (grey) and cavity (blue) are indicated in ‘**g**’ and ‘**h**’.

TWIK2 is notable for poor functional expression unless it is directed to the plasma membrane by mutations in lysosomal targeting motifs^29^. To test whether this localization change might affect channel structure or the lipid plug, we determined the structure of a previously characterized TWIK2 mutant^29,31,32^, denoted TWIK2_RM_ (for “Retention Mutant”), that is predominantly found at the plasma membrane as a result of a set of C-terminal tail mutations (I289A/L290A/Y308A)^29^. Initial cryo-EM analysis of TWIK2_RM_ showed one form corresponding to the lipid-bound state (Fig. S11). However, based on the functional effects of the TWIK2_RM_ mutations^29^, we decided to pursue further classification using a mask focused on the central cavity to determine if we could identify a form representing a functional channel. This analysis yielded two channel forms at 3.9 Å resolution each (Fig. S11, and Table 1) that we denoted as TWIK2_RM_-plugged and TWIK2_RM_-unplugged due to the presence or absence of the lipid plug. The structure of TWIK2_RM_-plugged was essentially identical to TWIK2:Det (RMSD_Cα_ = 0.7 Å) (Fig. 3a), showed Tyr111 in the up position (Fig. S12a), and two plug lipids blocking the central channel cavity (Figs. 3b, c and S12a). TWIK2_RM_-unplugged was also overall very similar to TWIK2:Det (RMSD_Cα_ = 0.7 Å) (Fig. 3d) and had Tyr111 from SF1 in the up position (Fig. S13a). Notably, the plug lipid density was absent (Figs. 3f and S13b). Instead, we found a spherical density just below the S4 position of the selectivity filter that we assigned as the “cavity ion” (Figs. 3d, e and S13a), reminiscent of results seen for a structure of K_2P_4.1 in the absence of its fenestration density^19^. Following the identification of the two forms, we reprocessed the TWIK2:ND and TWIK2:Det data using the same strategy used to obtain the plugged and unplugged forms of TWIK2_RM_, but failed to find a class corresponding to the unplugged form. These data suggest that the strong intracellular retention of the wild-type channel is associated with the lipid-plugged form, whereas some fraction of the channel lacks this plug when the channel can reach the plasma membrane. The region bearing the trafficking mutations is not seen in any of our structures, suggesting that this region is disordered. The density for the ions in the selectivity filter of both the TWIK2_RM_ plugged (Fig. S12a) and TWIK2_RM_ unplugged (Fig. S13a) structures suggests differences in ion occupancy at the S1 site from the TWIK2 structures. The structural analysis of the trafficking mutant TWIK2_RM_ shows that the channel can exist in two states, one having two lipid plug molecules (TWIK2:ND, TWIK2:Det, and TWIK2_RM_:Det plugged) and one in which access to the central cavity is unhindered (TWIK2_RM_:Det unplugged). Consistent with this change in ligand occupancy, we observed that TWIK2_RM_ was less stable during purification. Comparison of representative structures for the plugged (TWIK2:ND) and unplugged (TWIK2_RM_:Det unplugged) forms shows that the two plug lipids effectively block ion access to the channel by filling the central intracellular cavity below the selectivity filter (Fig. 3g, h).

### Functional studies highlight the importance of lipid-interacting cavity residues and Tyr111

**Fig. 4:**
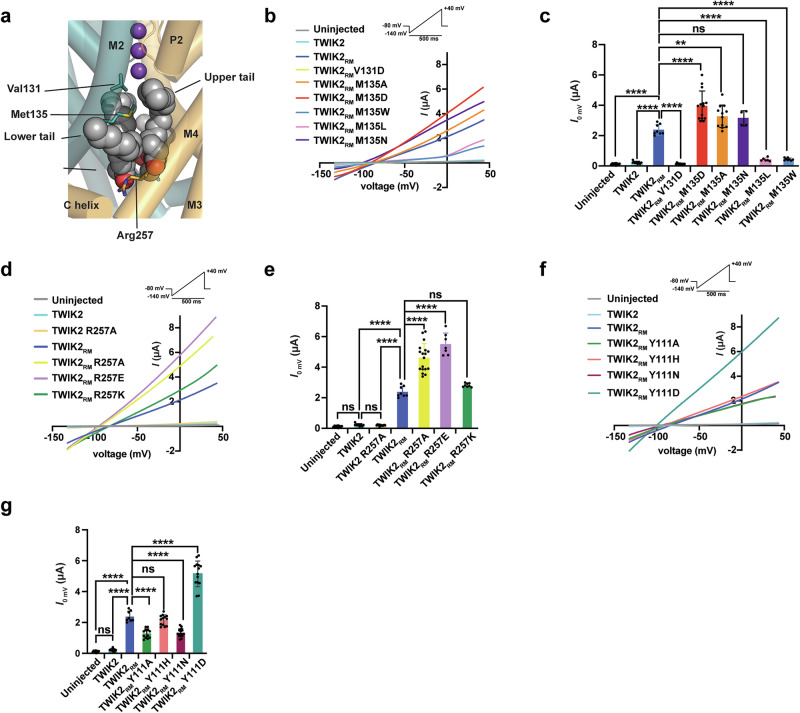
K_2P_6.1(TWIK2) cavity and filter sites affect function. **a** View of the K_2P_6.1(TWIK2) lipid plug from center of the membrane. Lipid plug is shown as grey spheres. Key interacting residues are shown as sticks. Potassium ions are purple spheres. **b** Exemplar current-voltage responses from TEVC *Xenopus* oocytes expressing the indicated channels and cavity mutants. Uninjected (grey), TWIK2 (sky blue), TWIK2_RM_ (blue), TWIK2_RM_ V131D (yellow), TWIK2_RM_ M135A (orange), TWIK2_RM_ M135D (red), TWIK2_RM_ M135W (teal), TWIK2_RM_ M135L (pink), and TWIK2_RM_ M135A (purple). Inset shows protocol. **c** Effects of mutants on K_2P_6.1(TWIK2) basal activity at 0 mV. ^****^p < 0.0001, ^***^p < 0.001, ^**^p < 0.01, ^*^p = 0.01–0.05. Exact *P* values for the comparisons are: Uninjected vs TWIK2_RM_ = < 0.0001, TWIK2 vs TWIK2_RM_ = < 0.0001, TWIK2_RM_ vs TWIK2_RM_ V131D = < 0.0001, TWIK2_RM_ vs TWIK2_RM_ M135D = < 0.0001, TWIK2_RM_ vs TWIK2_RM_ M135A = 0.006, TWIK2_RM_ vs TWIK2_RM_ M135L = < 0.0001, TWIK2_RM_ vs TWIK2_RM_ M135N = 0.3192, TWIK2_RM_ vs TWIK2_RM_ M135W = < 0.0001. Statistical analysis was performed using a One-Way ANOVA and multiple comparisons were performed with Tukey’s test. Error bars are S.E.M. **d** Exemplar current-voltage responses from TEVC *Xenopus* oocytes expressing the indicated channels and cavity mutants. Uninjected (grey), TWIK2 (sky blue), TWIK2_RM_ (blue), TWIK2 R257A (orange), TWIK2_RM_ R257A (yellow), TWIK2_RM_ R257E (lavender), and TWIK2_RM_ R257K (green). **e** Effects of mutants on K_2P_6.1(TWIK2) basal activity at 0 mV. ^****^p < 0.0001, ^***^p < 0.001, ^**^p < 0.01, ^*^p = 0.01 -0.05. Exact *P* values for the comparisons are: Uninjected vs TWIK2 = > 0.9999, TWIK2 vs TWIK2_RM_ = < 0.0001, TWIK2 vs TWIK2 R257A = > 0.9999, TWIK2_RM_ vs TWIK2 R257A = < 0.0001, TWIK2_RM_ vs TWIK2_RM_ R257A = < 0.0001, TWIK2_RM_ vs TWIK2_RM_ R257E = < 0.0001, TWIK2_RM_ vs TWIK2_RM_ R257K = 0.7768. Statistical analysis was performed using One-Way ANOVA and multiple comparisons were performed with Tukey’s test. Error bars are S.E.M**. f** Exemplar current-voltage responses from TEVC *Xenopus* oocytes expressing the indicated channels and mutants. Uninjected (grey), TWIK2 (sky blue), TWIK2_RM_ (blue), TWIK2_RM_ Y111A (green), TWIK2_RM_ Y111H (salmon), TWIK2_RM_ Y111N (maroon), and TWIK2_RM_ Y111D (teal). Inset shows protocol. **g** Effects of mutants on K_2P_6.1(TWIK2) basal activity at 0 mV. ^****^p < 0.0001, ^***^p < 0.001, ^**^p < 0.01, ^*^p = 0.01–0.05. Exact *P* values for the comparisons are: Uninjected vs TWIK2_RM_ = < 0.0001, Uninjected vs TWIK2 = > 0.9999, TWIK2 vs TWIK2_RM_ = < 0.0001, TWIK2_RM_ vs TWIK2_RM_ Y111A = < 0.0001, TWIK2_RM_ vs TWIK2_RM_ Y111H = 0.8821, TWIK2_RM_ vs TWIK2_RM_ Y111N = < 0.0001, TWIK2_RM_ vs TWIK2_RM_ Y111D = < 0.0001. Statistical analysis was performed using One-Way ANOVA and multiple comparisons were performed with Tukey’s test. Error bars are S.E.M. Uninjected *n* = 9, TWIK2 *n* = 9, TWIK2RM *n* = 8, TWIK2 R257A *n* = 12, TWIK2_RM_ R257A *n* = 18, TWIK2_RM_ R257E *n* = 7, TWIK2_RM_ R257K *n* = 8, TWIK2_RM_ V131D *n* = 11, TWIK2_RM_ M135D *n* = 14, TWIK2_RM_ M135A *n* = 12, TWIK2_RM_ M135L *n* = 6, TWIK2_RM_ M135N *n* = 5, TWIK2_RM_ M135W *n* = 8, TWIK2_RM_ Y111A *n* = 12, TWIK2_RM_ Y111H *n* = 13, TWIK2_RM_ Y111N *n* = 17 and TWIK2_RM_ Y111D *n* = 13 where ‘*n*’ represents the number of individual oocytes used.

We set out to test key features identified by the TWIK2 structures. As previously reported^23,29^, TWIK2 did not produce measurable currents in *Xenopus* oocytes using two electrode voltage clamp (TEVC), whereas mutation of the endolysosomal targeting signal (I289A/L290A/Y308A)^29^ (TWIK2_RM_) yielded readily measurable TWIK2 channels (Fig. S14a) that provided a platform for testing the effects of mutants. To test the importance of the lipid plug contacts, we examined the effects of mutations at the lipid plug-channel at three places along the channel central axis (Figs. 4a–c and S14a), with a first focus on mutations to negatively charged residues to destabilize lipid-channel contacts. V131D, located near the base of the selectivity filter, did not make functional channels (Figs. 4b, d and S14a). Introduction of acidic residues at Met135 (M135D) and Arg257 (R257E) increased basal activity with respect to TWIK2_RM_, as did replacement of Arg257 by alanine (R257A) (Figs. 4b, c and S14b). By contrast, conservation of the positive charge in the central cavity (R257K) yielded channels that were not different from TWIK2_RM._ Because the Met135 site corresponds to a hydrophobic barrier site in TWIK1 that contributes to a dewetting mechanism^45^, we investigated this site further. Mutations M135W/L/A/N/D yielded changes in the basal current of TWIK2_RM_ that followed similar trends for both hydrophobicity^46^ and sidechain volume^47^ (Fig. S14c), suggesting that the dewetting mechanism may be important in TWIK2. Comparison of ion selectivity by following the reversal potential as a function of external potassium showed no significant differences in the functional mutants from TWIK2_RM_ (Fig. S14d, e). The increased activity observed for mutation at sites that interact with the blocking lipids is consistent with the idea that removal of the lipid plug is key to channel function, while the trends at Met135 are also consistent with dewetting^45^, suggesting that both processes are important for TWIK2 function. Importantly, the observation that introduction of a negative charge and truncation to alanine at the Arg257 site increases activity points to an important role for this position in TWIK2 function.

The fact that Tyr111 is the largest residue seen at this filter position (Fig. 1c) and was seen in both up and down conformations prompted us to test the effect of replacing this residue with smaller, more commonly observed amino acids or with alanine. Substitution of this position in the TWIK2_RM_ background with asparagine (Y111N), the most common residue found at this site in SF1 of other K_2P_s^4^ (Fig. 1c), caused a reduction in basal current, as did replacement with alanine (Y111A) (Figs. 4f, g and S15a). By contrast, Y111H yielded basal currents similar to wild type (Fig. 4f, g), whereas substitution of Y111D, matching the most common residue at this position in SF2 as well as in most potassium channels^4,40^ yielded strongly activated channels and elevated basal currents (Figs. 4f, g, S14b and S15a). Although the closely related K_2P_1.1 (TWIK1) channel uses a histidine at the corresponding SF1 position for pH gating^6,48,49^, the Y111H change did not change the response of TWIK2 to external pH (Fig. S15b–d). Further, none of these changes affected selectivity as assessed from measuring the reversal potential as a function of external potassium (Fig. S15e). Notably, Y111A/N/H changes resulted in channels that showed inactivation (Fig. S15a) that was suppressed by elevated external potassium (Fig. S15f, g), supporting the importance of Tyr111 in the function of the K_2P_6.1 (TWIK2) selectivity filter gate.

### R257A mutation changes lipid plug position

**Fig. 5:**
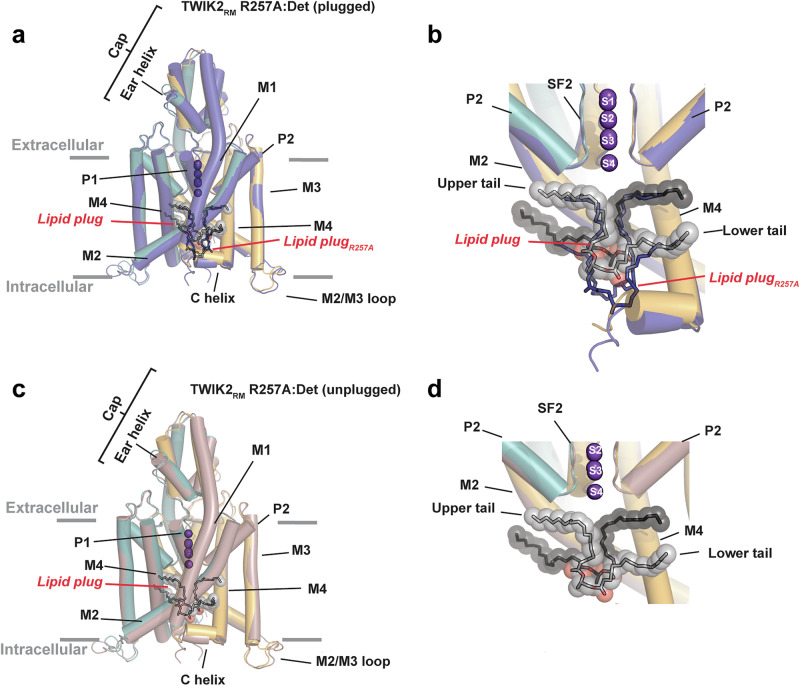
Comparison of plugged and unplugged K_2P_6.1 (TWIK2) R257A structures. **a** Superposition of TWIK2:ND (deep teal and bright orange) and TWIK2_RM_ R257A:Det (plugged) (deep blue) structures. **b** Close up view of the central cavity from ‘a’. Lipid plugs from TWIK2:ND and TWIK2_RM_ R257A:Det are shown as spheres (grey) and sticks (deep blue), respectively. **c** Superposition of TWIK2:ND (deep teal and bright orange) and TWIK2_RM_ R257A:Det (unplugged) (dirtyviolet) structures. **d** Close up view of the central cavity from ‘c’. Lipid plug from TWIK2:ND is shown as spheres (grey).

To investigate the structural consequences of perturbing lipid plug-channel interactions, we determined the structure of TWIK2_RM_ R257A, a mutant that increases TWIK2_RM_ basal currents (Figs. 4d, e and S16). Notably, the introduction of this mutation affected the stability of the channel during purification beyond what was seen with TWIK2_RM_. As with TWIK2_RM_, cryo-EM analysis of single particles of TWIK2_RM_ R257A using focused classification on the central cavity yielded two channel classes, one having the lipid plug, TWIK2_RM_ R257A-plugged (3.9 Å resolution), and one in which the plug was absent, TWIK2_RM_ R257A-unplugged (4.1 Å resolution) (Figs. S16–S18 and Table 1). The overall structures of both TWIK2_RM_ R257A forms were similar to the TWIK2:ND structure (Fig. 5a–d) (RMSD_Cα_ = 0.82 Å). Both had Tyr111 in the up position (Figs. S17a and S18a). TWIK2_RM_ R257A-plugged showed clear ion density at selectivity filter sites S1-S4 (Fig. S17a), whereas there were fewer well-defined ions in the TWIK2_RM_ R257A-unplugged form (Fig. S18a). Strikingly, in the TWIK2_RM_ R257A-plugged form, the lipid plug resides ~5 Å closer to the intracellular opening of the channel than in the other plugged TWIK2 structures (Figs. 5a-b and S19a). This change is consistent with the loss of stabilizing interactions with the Arg257 sidechain. Because of this lower position, ~four upper site methylene groups and ~ten lower site methylene groups are pulled into the central cavity leaving a smaller portion of the upper and lower tails in their respective binding sites (Figs. 5a, b and S19b). The observation that the R257A affects lipid plug position indicates that this residue has a key role in lipid plug binding and suggests that the functional effects from mutations at this site (Fig. 4d, e) are linked to alteration of lipid plug binding. Importantly, the fact that we can observe positional changes in the lipid plug provides corroborating evidence regarding its assignment and role as a crucial component of the TWIK2 structure.

### Arg257 makes dynamic interactions with the lipid plug

**Fig. 6:**
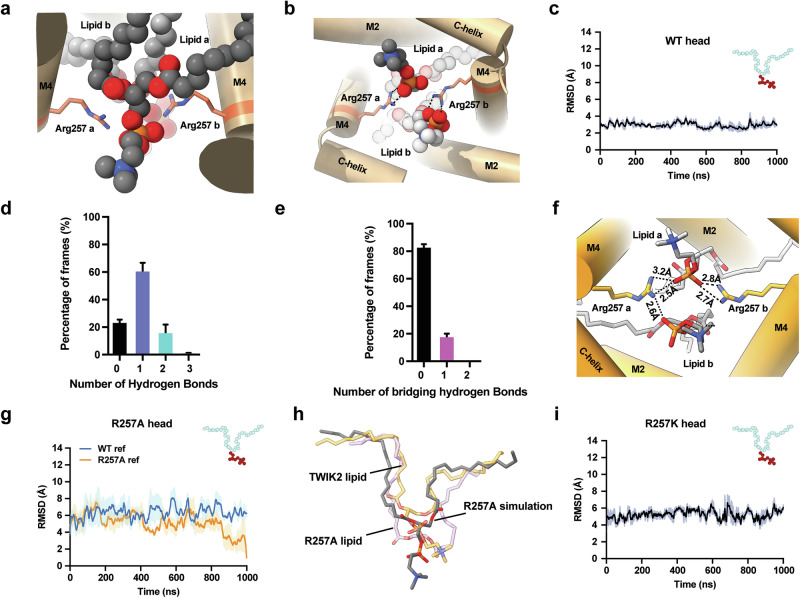
Simulations reveal dynamic interactions with plug lipids. Arg257 lipid interactions at start of simulation from the **a** side and **b** cytoplasmic views. **c** RMSD plot of the lipid head group heavy atoms relative to the starting position of the lipid head group heavy atoms from the TWIK2 simulation (*n* = 3). **d** Plot of Arg257-phosphate hydrogen bonds made by one sidechain (*n* = 3). **e** Plot of Arg257 bridging hydrogen bonds involving one Arg257 and two lipids (*n* = 3). **f** Simulation snapshot showing an exemplar bridging hydrogen bond interaction made by Arg257a. **g** RMSD plots of the lipid head heavy atoms relative to their starting position defined by the TWIK2:ND structure (WT ref) (blue) or the position of the lipid in the TWIK2 R257A structure (R257A ref) (orange) (*n* = 3). These indicate that the lipid moves from the WT position towards the position seen in the R257A structure. **h** Exemplar comparing positions of the R257A lipid from simulation (grey) with the lipid positions observed in the TWIK2 (bright orange) and R257A (magenta) experimental structures. Channel is not shown. and **i** RMSD plot of the heavy atoms of the lipid head group in the R257K simulation relative to the starting position of the heavy atoms of lipid head group defined by the TWIK2:ND structure (*n* = 3). Inset shows atom group. Lipid RMSDs are relative to the indicated reference cryo-EM structures. Shading in **c**, **g**, and **i** shows S.E.M. Error bars in **d** and **e** show S.E.M.

To investigate the nature of channel interactions with the plug further, we conducted 1 µs unrestrained all-atom molecular dynamics (MD) simulations in which the heads of the two lipid plug molecules were modeled as 1-palmitoyl-2-oleoyl-sn-*glycero*-3-phosphocholine (POPC) lipids (Supplementary Tables S1 and S2). Constraints from the overall TWIK2 architecture position the Arg257 sidechains at the level of the phosphoglycerol backbone of the lipid (Fig. 6a, b). The simulations show stable positions for both lipid plug members as monitored at the position of the lipid heads (Fig. 6c) and tails (Fig. S20a–f). Unsurprisingly, the ends of the lipid tails that emerge from the lipid-occupied cavities showed the most mobility (Fig. S20c, f). Strikingly, the simulations revealed that the Arg257 sidechains sample multiple classes of interactions with the lipids involving 1-3 hydrogen bonds and conformations in which the guanido moiety coordinated more than one lipid (Fig. 6d–f, and Supplementary Movie 1). This dynamic nature of the Arg257-lipid interactions is consistent with the absence of well-defined density in the experimental maps for this sidechain.

To probe the importance of the Arg257-lipid plug interaction further, we ran simulations in which the Arg257 sidechain was replaced by alanine. The R257A change results in higher mobility of the lipid in the lipid head moiety (Fig. 6g) and both lipid tails (Fig. S20g–l). Interestingly, we observed that during the simulation lipid plug begins to drop towards the intracellular side of the channel towards the position observed in the R257A structure (Fig. 6g, h). These observations further support the importance of the Arg257-lipid interactions in stabilizing the lipid plug. The movement of the lipid towards the cytoplasmic side of the channel was not completed in the timescale of the 1µs simulations, suggesting that longer times are required to reach the position observed experimentally. Finally, we investigated the consequence of replacing Arg257 with another positively charged sidechain. Simulations of the R257K mutant showed stable positions for all lipid plug elements, similar to those observed for the wild-type channel (Fig. 6i and S20m–r). Moreover, as in the WT simulations, the lower tail appeared more mobile than the upper tail.

The lipid plug density in the TWIK2 Cryo-EM maps has poorer local resolution than the surrounding protein regions. To investigate the link between this property and lipid flexibility, we calculated time-averaged density maps^50^ from the TWIK2, R257A, and R257K 1μs all-atom MD simulations. The resultant maps (Fig. S21d–l) show density that matches our experimental observations. Notably, at stringent density thresholds, the lipid head density is lost, whereas the density in the two lipid tail binding cavities remains. These results qualitatively recapitulate the behavior of the experimental densities, with clear enrichment of the upper and lower lipid tail positions in the respective fenestrations observed in the structures. Taken together, the simulations support both the stability of the lipid plug and emphasize the important role of the Arg257 position in lipid plug stabilization.

## Discussion

A puzzling aspect of many ion channel structures has been the identification of fenestrations from the central cavity that contain lipid or lipid-like densities that could represent elements that interfere with the passage of ions through the channel central cavity^5,19,36,51^. In particular, this phenomenon has been observed for a number of K_2P_s, including K_2P_1.1 (TWIK1)^5,6^, K_2P_4.1 (TRAAK)^19,36^, K_2P_2.1 (TREK1)^37^, and K_2P_10.1 (TREK2)^9^. However, in each case, the reported densities only define a single alkyl chain^5,19^, leaving open the possibility that its origin could be detergents required for channel purification rather than a lipid^5,37^. One interpretation of this density is that it represents a mechanism in which horizontal lipid access from the bilayer acts as a gating mechanism^6,19^; however, this issue remains controversial^20,21,52^, particularly as simulations indicate that even though lipid tails can enter the cavity from the bilayer, they do not block the pore^20,21^.

Here, we provide evidence for TWIK2 block by a pair of two-tailed lipids, the lipid plug, in which each lipid tail fills a separate binding site that extends towards the bilayer, and the lipid headgroup points towards the inner cytoplasmic face in an orientation that matches that of bilayer inner leaflet lipids. The upper tail site overlaps with the position assigned as a single alkyl chain in the initial structure of K_2P_1.1 (TWIK1)^5,6^ framed by P2, M2, and M4 (Fig. 2b). The lower tail passes through a gap on the opposite side of the channel between M2, M4, and the C-helix. In this binding mode, each of the plug lipids spans the inner cavity. We note also that both sites are occupied by alkyl chains in a recent 3.7 Å structure of TWIK2 in detergent^43^. Given the high similarity between TWIK1 and TWIK2 structures (Fig. S3a–c), excellent overlap of the upper tail site (Fig. 3b), and presence of the lower cavity in TWIK1^21^ (Fig. S8d), our data suggest that the lipid block we observe occurs in both channel types. This interpretation is strengthened by the fact that cryo-EM maps from TWIK1 in an inactive (pH 5.5) and active (pH 7.4) form have a bifurcated density in the central cavity that is well matched by the lipid plug structure derived from our TWIK2 studies (Figs. 2e and S8e). Thus, previous observations of density in the upper tail site may represent incomplete density for similar lipid plug structures arising from lipid dynamics, an interpretation that is in line with our density analysis from MD simulations (Fig. S21d–l). Together, these data reveal a lipid binding mode that, to the best of our knowledge, has not been noted previously and offer a reinterpretation of previous findings regarding how lipids bind TWIK channel fenestrations. Structural analysis of the R257A mutant that removes interactions with the blocking lipids leads to a repositioning of the lipid plug and suggests an important role for the conserved Arg257 residue in TWIK2 channels. It is worth noting that TWIK1 and TWIK2 have narrower inner pores than channels from the TREK subfamily, suggesting that the lipid plug structure is a distinct feature of the TWIK subfamily.

**Fig. 7:**
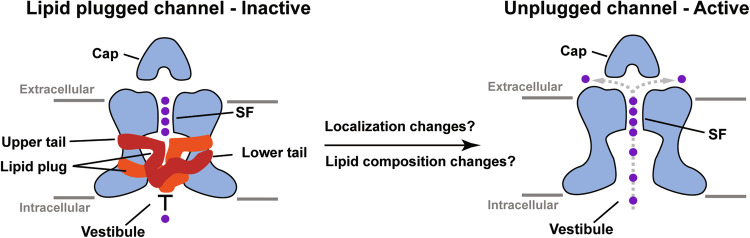
Working model of K_2P_6.1(TWIK2) regulation. Lipid plug prevents ion passage. Removal of plug in response to cellular localization changes, changes in the membrane lipid composition, or other factors yields channels that can conduct ions.

Ion concentrations inside various intracellular organelles vary, and there are a growing number of ion channels whose activity affects diverse intracellular compartments^53^, including TWIK2^54^. Because every ion channel has the potential to perturb ion flux, it is essential that these protein pores open only in the right place at the right time. The majority of channels in the VGIC superfamily that are activated by voltage or ligands have an intracellular gate that blocks ion passage, rendering them inactive in the absence of a stimulus^55^. This structural barrier avoids openings in the wrong subcellular compartment. Although their activity can be tuned by various types of inputs^2,4^, the fundamental property of K_2P_s is that they are leak channels, lacking the strongly closed state of their VGIC superfamily relatives^2,4^. Consequently, their basal activity gives K_2P_s the capacity to influence ion flux across both internal and external membranes as they transit through various cellular compartments during their life cycle. Such basal activity also has the potential to inadvertently lead to aberrant functional effects if it is unregulated. We propose that the propensity of TWIK channels to be plugged by lipids represents a regulatory mechanism that contributes to the ability of a cell to control K_2P_ function (Fig. 7). Such changes in activity may be linked to processes in which channel activation involves redistribution of TWIK2 channels from intracellular organelles to the plasma membranes, as has been suggested during macrophage inflammasome activation^27,30–32^. The membranes of different cellular compartments have diverse lipid compositions^56,57^ and there is a growing appreciation of the importance of lipid nanoenvironments in controlling membrane protein function^56^. These factors raise the possibility that changes in local lipid nanoenvironments have a role in setting the proportion of plugged versus unplugged forms. Together, our observations suggest the hypothesis that TWIK family activation is a “right place, right time” process in which the channels remain plugged until they arrive at the cellular destination where their activity is required.

Although our data show that the channel can exist in both plugged and unplugged forms, the identity of the lipids and how the lipid plug moves in and out is unclear. Corroborating the structural data, MD simulations show that the lipid plug is stable in the channel vestibule. The presence of the plug is incompatible with ion movement through the central cavity (Fig. 3h). Hence, its removal from the cavity is essential to yield a functional channel. How this can happen is not obvious. Changes in the positions of the M4 and the C-helix would offer a potential exit path, as such motions would alter the size of the lateral gap facing the bilayer that forms the most natural way for lipid egress. Whether such conformational transformations constitute an autonomous mechanism driven by changes in lipid bilayer properties or require the intervention of factors that facilitate lipid removal remains an open question in need of investigation. Wetting-dewetting of the narrow hydrophobic inner pore has been proposed as an important regulatory mechanism for TWIK channels that can be influenced by occupation of the lateral fenestration by lipid tails^21,45,58^. As such, there may also be some role for the competition between water and the lipid plug. Importantly, the dewetting and plug mechanisms are not mutually exclusive and may both contribute to TWIK function. Simulations of the channel and Arg257 mutants that enhance function could provide additional insight into the role of dewetting and potential electrostatic barrier resulting from Arg257, as well as possible mechanisms for lipid ingress and egress. Further, a recent structure of TWIK2 that was reported while this work was in preparation suggests that the density previously assigned to a single alkyl chain that we assign to the upper tail of the plug can be displaced by pimozide, a drug that modulates TWIK2, raising the possibility of competition between the lipid plug and small molecule modulators of K_2P_ function^42^. We note that our proposed model (Fig. 7) is not a “gating mechanism” for moment-to-moment control of channel function, but outlines a regulatory pathway that can explain the two states that we observe.

Besides bringing insights into mechanisms of lipid modulation, the TWIK2 structure highlights the unusual divergence from the canonical selectivity filter sequences afforded by the heteromeric nature of the K_2P_ SF^4,39^. The presence of a large, non-canonical residue at Y111, a site usually occupied by smaller residues (Fig. 1c), shows two conformations. The “up” conformation is predominant in our structures but is also accompanied by a “down” conformation in the lipid nanodisc complex that matches the pose found for the equivalent position in other K_2P_s (Fig. S7b–d). These “up”/“down” conformations match those of a regulatory histidine, His98, in K_2P_3.1 (TASK-1)^11,44^ (Fig. S7e), and together with the functional effects of Tyr111 mutations (Figs. 4f, g and S15a, f, and g) highlight the role of this site in K_2P_ function and the importance of the K_2P_ C-type selectivity filter gate^14,15,18,59^ for TWIK2 function. The observation that the “up”/“down” rotamer change can occur in different K_2P_ subfamilies, together with additional conformational changes reported for the equivalent SF1 position in K_2P_1.1 (TWIK1)^6^ (Fig. S7f) and K_2P_2.1(TREK-1)^17^ under conditions associated with inactivation of the channel C-type SF gate, highlights the functional importance of structural changes at this site.

Structures of TWIK2 in lipid and detergent environments show that this channel conforms to the overall architecture found throughout the K_2P_ family and is most similar to the other structurally characterized member of the TWIK subfamily, TWIK1. Nevertheless, the TWIK2 structure revealed three additional elements: an extra helix in the extracellular cap domain, the ear helix, which connects the cap with the loop leading to the first pore helix (P1), conformational variability at a position in SF1 that is linked to C-type gating in other K_2P_s^6,11,17,44^, and the lipid plug. Understanding the roles of these elements in K_2P_6.1 (TWIK2) function will be important for dissecting the biological functions of this channel, including how it affects macrophage NLRP3 inflammasome activation^27,30–32^. Our reinterpretation of how lipids affect TWIK channels by acting as a plug that binds to two lateral gaps in the channel pore sets a framework for addressing how ion channel-lipid interactions control function that may have general relevance for other ion channel classes.

## Methods

### Ethical statement

Oocytes were harvested from female *Xenopus* laevis frogs (Nasco Cat# LM00531) and housed in the UCSF Laboratory Animal Resource Center (LARC) facilities. The use of these *Xenopus* oocytes was approved by IACUC (protocol approval # AN193390 - 01B) and experiments were performed in accordance with University of California guidelines and regulations.

### Expression and purification of K_2P_6.1_WT_ and mutants

The gene for a construct of the human K_2P_6.1 (TWIK-2) (Uniprot ID: Q9Y257), K_2P_6.1_WT_, spanning residues 1-313 followed by a 3C protease cleavage site, monomeric enhanced green fluorescent protein (mEGFP), and a His_8_ tag, was cloned into a modified pFastBac expression vector in which the polyhedrin promotor was replaced by a mammalian cell-active CMV promotor^60^. Expression vectors for TWIK-2_RM_ (I289A, L290A and Y308A) and TWIK-2_RM_ R257A mutants were made on this background. These vectors were used for recombinant bacmid DNA generation using chemically competent DH10EmBacY (Geneva Biotech) cells. These bacmids were then used to transfect *Spodopetera frugipdera* (SF9) cells to make baculoviruses for the constructs of interest^61^.

Expi293F cells (Gibco A14528) were grown to 2.5–3 × 10^6^ cells mL^-1^ at 37 °C supplemented with 8% CO_2_ shaking at 120 RPM, before transduction with 10% (v/v) baculovirus stock of K_2P_6.1_WT_ or mutants. 12–14 h after transduction, 10 mM sodium butyrate was added to the culture to enhance protein expression^62^. Flasks were then moved to 30 °C for 48 h before harvesting the cells using centrifugation at 2300 × *g* for 20 min. The pellet was gently washed with Dulbecco’s phosphate-buffered saline (Gibco 14190144) to remove leftover culture media, centrifuged (3500 × *g* for 20 min) to recover the cell pellet, and then flash frozen with liquid nitrogen for storage at – 80 °C.

The pellet from 1.8 L culture was resuspended in 50 mL hypotonic buffer (20 mM KCl, 10 mM Tris-HCl, pH 8.0, phenylmethylsulfonyl fluoride (PMSF), 0.1 mg mL^−1^ DNase1, supplemented with Pierce protease inhibitor (Thermo Fischer Scientific) and was gently stirred on a Mono Direct Variomag magnetic stirrer (Thermo Fischer Scientific)) at 4 °C for 30 min. The membranes were isolated by ultracentrifugation at 185,500 × *g* for 30 min. Using a 16G Precision Glide needle and 30 mL syringe, the membrane pellet was homogenized in 200 mL solubilization buffer (buffer S) (100 mM KCl, 50 mM Tris-HCl, pH 8.0, supplemented with 1 mM PMSF, 0.1 mg mL^−1^ DNase1, 1% (w/v) n-Dodecyl-β-D-Maltopyranoside (β-DDM), and Pierce protease inhibitor). Membrane solubilization and protein extraction were performed by gently stirring at 4 °C for 3 h followed by ultracentrifugation at 185,500 × *g* for 40 min to remove cell debris. 5 ml of anti-GFP nanobody conjugated resin conjugated CNBr Sepharose beads (GE Healthcare, #17-0430-02)^63^ pre-equilibrated with 200 mM KCl, 0.01% β-DDM, 20 mM Tris-HCl pH 8.0 buffer (buffer C), was added to the supernatant after the ultracentrifugation and incubated at 4 °C with gentle rotation on a Boekel 260200 Orbiton platform rotator. Subsequently, the resin was collected on a gravity flow column and washed with 10 column volumes (CV) of buffer A (200 mM KCl, 20 mM Tris-HCl pH 8.0, 0.1% β-DDM), followed by another wash step with 10 CV of buffer B (200 mM KCl, 20 mM Tris-HCl pH 8.0, 0.05% β-DDM), and then 10 CV of buffer C (200 mM KCl, 20 mM Tris-HCl pH 8.0, 0.01% β-DDM). On column cleavage of the affinity tag was achieved by incubating the resin overnight at 4 °C without any shaking with buffer C supplemented to contain 200 mM KCl, 1 mM EDTA, and 3C protease at a ratio of 50:1 resin volume:protease volume. The cleaved sample was collected in 50 mL Falcon tube, and the resin was subsequently washed with 2 column volumes (CV) of buffer C. The wash was also collected in the same 50 mL Falcon tube. This purified sample was concentrated using an Amicon Ultra-15 centrifugal unit with 100 kDa cutoff and applied to a Superdex 200 increase column pre-equilibrated with buffer C. Peak fractions were analyzed by SDS-PAGE and concentrated to 2 mg mL^−1^ for cryo-EM sample preparation using an Amicon Ultra-0.5 ml centrifugal unit with 100 kDa cutoff.

For nanodisc (ND) reconstitution, Brain Extract Polar lipid (Avanti polar lipids #141101P) was prepared by dissolving 100 mg of lipid in 0.2 mL chloroform, drying under nitrogen gas, and followed by lyophilization. The lyophilized lipid was dissolved by sonication in 1 mL of lipid buffer (200 mM KCl, 50 mM Tris, pH 8.0, 0.5% β-DDM). TWIK2 from the size-exclusion chromatography step, purified MSP1E1^38^, and prepared lipid stock were incubated together at 1:5:250 molar ratio (nanodisc reconstitution mix) for 1 h in a 1.5 mL Eppendorf microcentrifuge tube at 4 °C. Bio-beads SM-2 adsorbents (Bio-rad #1528920) were prepared by washing 200 mg bio-beads with 1 mL methanol, followed by 1 mL water and then with 1 mL buffer N (200 mM KCl, 20 mM HEPES pH 7.4). 100 mg of bio-beads equilibrated with buffer N were added to 1 mL nanodisc reconstitution mix and incubated for 1 h, gently rotating at 4 °C. After 1 h the reconstitution mix was transferred to a clean 1.5 mL Eppendorf tube and incubated with another 100 mg of equilibrated bio-beads and was rotated gently at 4 °C overnight. The supernatant from the reaction mixture was injected onto Superose 6 increase 10/300 column pre-equilibrated with buffer N. Peaks were analyzed by SDS-PAGE and concentrated to 3 mg mL^−1^ for cryo-EM sample preparation using an Amicon ultra-0.5 mL centrifugal unit with 100 kDa cutoff.

Twik-2_RM_ and Twik-2_RM_R257A were expressed and purified by following identical methods as used for Twik-2_WT_. Following the final Superose 6 Increase 10/300 GL purification step, purified samples were concentrated using Amicon ultra-0.5 mL centrifugal units to 3 mg mL^−1^ for cryo-EM sample preparation.

### Cryo-EM sample preparation and data collection

3.5 μL of concentrated protein sample was applied to a glow-discharged (30 s at 15 mA) grid (Quantifoil Au R1.2/1.3 300 mesh) and after a wait time of 5 s, grids were blotted once for 2–6 s (4 °C and 100% humidity) using a FEI Vitrobot Mark IV (Thermo Fisher Scientific) and plunge-frozen in liquid ethane. The grids were screened using Serial EM^64^ on 200 kV Talos Arctica cryo-TEM (Thermo Fisher Scientific) equipped with K3 direct detector camera (Gatan) at the University of California, San Francisco (UCSF) EM facility. Movies were collected using Serial EM^64^ and EPU (Thermo Fisher Scientific, https://www.thermofisher.com/us/en/home/electron-microscopy/products/software-em-3d-vis/epu-software.html) data acquisition software on 300 keV FEI Titan Krios (Thermo Fisher Scientific) at UCSF and SLAC National Accelerator Laboratory, respectively. Both microscopes were equipped with a K3 direct-electron detector and post-BioQuantum GIF energy filter (Gatan), set to a slit width of 20 eV. Super-resolution counting mode was used at a nominal magnification of ×105,000 with a pixel size of 0.43 Å (SLAC) and 0.42 Å (UCSF). A nominal defocus range between −1.1 and −2.5 μm was used with total dose of 60 e^-^ / Å^2^, having a total exposure time of 2.8 s at SLAC and −1.1 to -−2.2 μm defocus range was used with total dose of 47.7 e^-^ / Å^2^, having total exposure time 2 s at UCSF EM facility.

### Structure determination (image processing, model building and refinement)

A total of 10,315, 13,561, 11,690 and 24,106 movies were collected for TWIK2:ND, TWIK2:Det, TWIK2_RM_:Det and TWIK2_RM_R257A:Det samples, respectively. Cryo-EM data processing was performed using cryoSPARC (version 4.5)^65^ and Relion (v5)^66^. Patch motion correction (2x binned) and patch CTF estimation were performed before manually curating the exposures by ice-thickness, CTF-fit resolution. Selected micrographs were used for reference-free circular blob picker (diameter 60–120 Å) for particle picking, followed by extraction at a box size of 260 Å. Several rounds of 2D classification were performed and further cleaning was done by categorizing particles by 3D Ab initio reconstruction using C1 symmetry. Non-uniform refinement was performed on the particle set belonging from best ab-initio map using C1 symmetry. Finally, non-uniform and local refinement were performed using C2 symmetry to improve resolution and map quality. Datasets corresponding to TWIK2_RM_ and TWIK2_RM_R257A yielded plugged conformations after non-uniform refinement. The refined particle sets for each of these datasets were subjected to 3D classification using a focused mask corresponding to the lipid-plug density obtained in non-uniform refinement. The 3D classification segregated the total particle stacks into two major groups (plugged and unplugged). These two groups were further subjected to local refinement to obtain the final maps corresponding to Twik-2_RM_ (plugged and unplugged) and Twik-2_RM_R257A (plugged and unplugged). A similar approach, generating a lipid plug mask using the density observed in the vestibule region and using it for focused 3D classification, was attempted to find un-plugged conformations in the TWIK2:ND and TWIK2:Det datasets. However, this approach did not generate any 3D class without a lipid plug for these two datasets, indicating an absence of the unplugged class. All 3D classifications used C1 symmetry.

A preliminary model based on K_2P_2.1 (TREK-1)(PDB ID: 8UE2)^67^ was converted to poly-alanine and used to dock as a backbone in the density using phenix.dock_in_map (v1.21.2)^68^. Multiple rounds of iterative model building and refinement were performed using Coot (v0.9.8.95)^69^ and phenix.real_space_refine^70^, respectively. Final geometry validation statistics were calculated by MolProbity^71^. Figures were prepared, and model comparisons were performed using UCSF ChimeraX (version 1.15)^72^ and the Pymol package (http://www.pymol.org/pymol). Contact analysis was performed using LIGPLOT^73,74^.

### Two-electrode voltage-clamp (TEVC) electrophysiology

*Xenopus laevis* oocytes were harvested according to UCSF IACUC Protocol AN193390 and digested using collagenase (Worthington Biochemical Corporation, #LS004183, 0.8–1.0 mg mL^−1^) in Ca^2+^-free ND96 (96 mM NaCl, 2 mM KCl, 3.8 mM MgCl_2_, 5 mM HEPES pH 7.4). Prior to RNA injection, oocytes were maintained at 4 °C in ND96 (96 mM NaCl, 2 mM KCl, 1.8 mM CaCl_2_, 2 mM MgCl_2_, 5 mM HEPES pH 7.4) supplemented with antibiotics (100 units ml^−1^ penicillin, 100 µg ml^−1^ streptomycin, 50 µg ml^−1^ gentamicin) and used for experiments within 1 week of harvest.

Human K_2P_6.1 (TWIK-2) (Uniprot ID: Q9Y257) was subcloned into a previously reported pGEMHE/pMO vector^14^. Site-directed mutations were made using Inverse PCR ^75^ with fully overlapping primers. mRNA for oocyte injections was prepared from linearized plasmid DNA (linearized with AflII) using the mMessage Machine T7 Transcription Kit (Thermo Fisher Scientific). RNA was purified using RNEasy kit (Qiagen) and stored as aliquoted stocks at −80 °C. Defolliculated stage V-VI oocytes were microinjected with 11–13 ng mRNA and incubated at 18 °C for expression. Currents were recorded within 48–54 h after microinjection. For amplitude comparisons, currents were compared at the same time of expression for sample injected with equivalent amounts of mRNA.

For recordings, oocytes were impaled with borosilicate recording microelectrodes (0.2–2.0 MΩ resistance) backfilled with 3 M KCl and were subjected to constant perfusion of ND96 at a rate of 3–5 ml min^−1^. Except where otherwise indicated, the recording solution was 96 mM NaCl, 2 mM KCl, 1.8 mM CaCl_2_, and 1.0 mM MgCl_2_, buffered with 5 mM HEPES, pH 7.4. For each recording, control solution (ND96) was perfused over a single oocyte until the current stabilized. Currents were evoked from a −80 mV holding potential, followed by a 500 ms ramp from −140 to +50 mV. Data were recorded using an Axoclamp 900 A amplifier (Molecular Devices) controlled by pCLAMP10.7 software (Molecular Devices), and digitized at 1 kHz using a Digidata 1550B digitizer (Molecular Devices). Representative traces and dose response plots were generated in GraphPad Prism 10 (GraphPad Software, Boston).

### Data quantification and statistical analysis

The results are from 3 to 5 independent oocyte batches. *P* values for Fig. 4c are as follows: *P* = 0.9991 (K_2P_6.1(TWIK2) vs uninjected oocytes), *P* < 0.0001 (TWIK-2 vs TWIK-2_RM_), *P* < 0.0001 (TWIK-2_RM_ vs TWIK-2_RM_ V131D), *P* = 0.0060 (TWIK-2_RM_ vs TWIK-2_RM_ M135A), *P* < 0.0001 (TWIK-2_RM_ vs TWIK-2_RM_ M135D), *P* = 0.3193 (TWIK2_RM_ vs TWIK2_RM_ M135N), (TWIK2_RM_ vs TWIK2_RM_ M135L), and *P* < 0.0001 (TWIK2_RM_ vs TWIK2_RM_ M135W). Statistical analysis was performed using One-Way ANOVA, and multiple comparisons were performed with Tukey’s test. Error bars are S.E.M. Full detailed statistical information is available in the source data spreadsheet.

*P* values for Fig. 4e are as follows: *P* = 0.9991 (K_2P_6.1(TWIK2) vs uninjected oocytes), *P* < 0.0001 (TWIK2 vs TWIK2_RM_), *P* > 0.9999 (TWIK2 vs TWIK2 R257A), *P* < 0.0001 (TWIK2_RM_ vs TWIK2 R257A), *P* < 0.0001 (TWIK2_RM_ vs TWIK2 _RM_ R257A), *P* < 0.0001 (TWIK2_RM_ vs TWIK2 _RM_ R257E), and *P* = 0.7768 (TWIK2_RM_ vs TWIK2 _RM_ R257K). Statistical analysis was performed using One-Way ANOVA, and multiple comparisons were performed with Tukey’s test. Error bars are S.E.M. Full detailed statistical information is available in the source data spreadsheet.

*P* values for Fig. 4g are as follows: *P* = 0. 9991 (TWIK2 vs uninjected oocytes), *P* < 0.0001 (TWIK2 vs TWIK2_RM_), *P* < 0.0001 (TWIK2_RM_ vs TWIK2_RM_ Y111A), *P* = 0. 8821 (TWIK2_RM_ vs TWIK2_RM_ Y111H), *P* < 0.0001 (TWIK2_RM_ vs TWIK2_RM_ Y111N) and *P* < 0.0001 (TWIK2_RM_ vs TWIK2_RM_ Y111D). Statistical analysis was performed using One-Way ANOVA, and multiple comparisons were performed with Tukey’s test. Error bars are S.E.M. Full detailed statistical information is available in the source data spreadsheet.

### Molecular dynamics simulations

The TWIK-2:ND cryo-EM structure, modeled with complete POPCs in the positions of the plug lipids, was used as the starting model for molecular dynamics (MD) simulations and for generating in silico mutants. All residues were assigned standard protonation states at pH 7.0, and the N- and C-termini were capped with acetyl and methyl amide groups, respectively, using CHARMM-GUI^76^. Each system was embedded in a pure 1-palmitoyl-2-oleoyl-sn-glycero-3-phosphocholine (POPC) bilayer and solvated with 150 mM [K^+^] with neutralizing [Cl^-^]. The CHARMM36m^76^ and CHARMM36^77^ force fields were used for the protein and lipids, respectively, while water molecules were represented with the TIP3P^78^ model and POPC parameters were taken from CGenFF v3.0.1^79–81^. Standard CHARMM ion parameters were used for K⁺ and Cl⁻^82^.

All systems were energy-minimized for 8000 steps with 5 kcal·mol⁻¹·Å⁻² harmonic restraints applied to protein heavy atoms, followed by a multistage equilibration during which positional restraints were gradually reduced over 12 ns. Production simulations were performed in GROMACS-2025.2^83^ using a 2-fs integration time step. Temperature was maintained at 303 K using a velocity-rescaling (v-rescale) thermostat^84^ applied independently to the protein–membrane complex and solvent. Semi-isotropic pressure coupling at 1 atm was maintained with a C-rescale barostat^85^ (τ_p_ = 5 ps). Nonbonded interactions were computed with a 12 Å cutoff using a force-switching function between 10 and 12 Å. Long-range electrostatics were treated with the particle-mesh Ewald (PME) method^86^, and all covalent bonds involving hydrogen atoms were constrained using the LINCS algorithm^87^.

The root-mean-square deviation (RMSD) and root-mean-square fluctuation (RMSF) of individual lipid plug segments were calculated from three independent 1μs MD simulation replicas of TWIK2_WT_, TWIK2_R257K_ and TWIK2_R257A_ using the GROMACS-2025.2 (10.5281/zenodo.15387070) tools gmx rms and gmx rmsf, respectively. For the analysis, the trajectories were aligned on the Cα atoms of the protein using the TWIK2:ND Cryo-EM structure as the reference conformation, and RMSD/RMSF values were computed for the heavy atoms of the lipid segments. RMSD raw traces were smoothed over a 1 ns (10 frames) window. Error bars represent the standard deviation across the three independent replicas. The percentage of hydrogen bonds formed between the lipid plug headgroups and the guanidinium group of Arg257 was calculated using the gmx hbond tool in GROMACS-2025.2. The time-averaged lipid density map^50^ was calculated from the 1μs MD trajectories using the gmx spatial tool in GROMACS-2025.2.

### Reporting summary

Further information on research design is available in the Nature Portfolio Reporting Summary linked to this article.

## Supplementary information

Supplementary Information

Description of Additional Supplementary Files

Supplementary Movie 1

Reporting Summary

Transparent Peer Review file

## Source data

Source Data

## Data Availability

Coordinates and maps of the following have been deposited in Protein Data Bank (PDB) and Electron Microscopy Data Bank (EMDB): 9OTA, EMD-70828, EMD-75026 (K_2P_6.1 (TWIK2) nanodisc) and 9OTK, EMD-70837 (K_2P_6.1 (TWIK2) detergent). 9OV0, EMD-70885 (K_2P_6.1 (TWIK2_RM_) plugged) and 9OV9, EMD-70894 (K_2P_6.1 (TWIK2_RM_) unplugged). 9OVD, EMD-70898 (K_2P_6.1 (TWIK2_RM_) R257A plugged) and 9OVE, EMD-70899 (K_2P_6.1 (TWIK2_RM_) R257A unplugged). Prior structures and maps for analysis include 3UKM (K_2P_1.1 (TWIK-1)), 7SK0 (K_2P_1.1 (TWIK-1) pH 7.4), 7SK1 (K_2P_1.1 (TWIK-1) pH 5.5), 6CQ6 (K_2P_2.1 (TREK-1)), 4XDK (K_2P_10.1(TREK-2):norfluoxetine (Nfx) complex), 9BSN (K_2P_13.1(THIK-1)), 6RV2 (K_2P_3.1(TASK-1)), 6WLV (K_2P_5.1(TASK-2) pH 6.5), 6WM0 (K_2P_5.1(TASK-2) pH 8.5), EMD-25169 (K_2P_1.1 (TWIK1) at pH5.5). Human K_2P_6.1 (TWIK2) sequence is Uniprot Q9Y257. The coordinates for the first and last frames from MD simulations have been deposited in Zenodo (https://zenodo.org/records/20435921). Source Data are provided with this paper. Data or materials will be provided on request from the corresponding author. Source data are provided with this paper.
