# Supplementary material for: Structural evidence that a lipid plug controls K2P6.1(TWIK-2) function
Source: Nat Commun. 2026 Jun 16;17:7959. doi: 10.1038/s41467-026-74535-6 (PMC13448071; doi:10.1038/s41467-026-74535-6)
Supplement: Supplementary file 1 [file 41467_2026_74535_MOESM1_ESM.pdf]

8 June 2026

Supplementary Material for:

Structural evidence that a lipid plug controls K_{2P}6.1(TWIK-2) function

Abhisek Mondal[1], Sangeeta Niranjan[1], and Daniel L. Minor Jr.[1-6]*

[1]Cardiovascular Research Institute, University of California, San Francisco, CA 93858-2330 USA

[2]Department of Biochemistry and Biophysics, University of California, San Francisco, CA 93858-2330 USA

[3]Department of Cellular and Molecular Pharmacology, University of California, San Francisco, CA 93858-2330 USA

[4]California Institute for Quantitative Biomedical Research, University of California, San Francisco, CA 93858-2330 USA

[5]Kavli Institute for Fundamental Neuroscience, University of California, San Francisco, CA 93858-2330 USA

[6]Molecular Biophysics and Integrated Bio-imaging Division
Lawrence Berkeley National Laboratory, Berkeley, CA 94720 USA

*Correspondence to: daniel.minor@ucsf.edu

Figure S1 Mondal, et al.

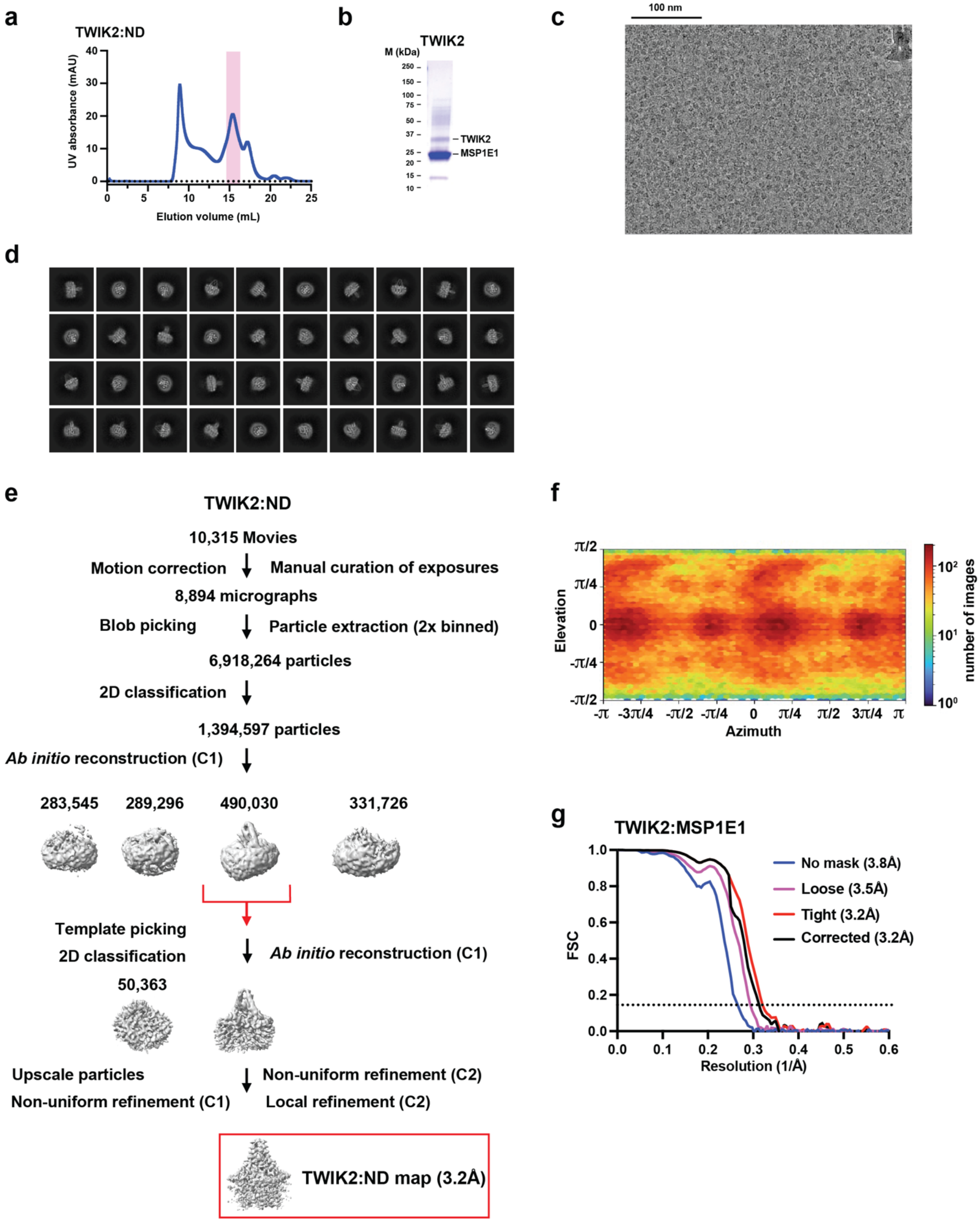

Figure S1 Cryo-EM analysis of TWIK2 in MSP1E1 nanodiscs Exemplar **a,** SEC (Superose 6 Increase 10/300 GL) for TWIK2:MSP1E1 nanodiscs (TWIK2:ND), **b,** peak fraction SDS-PAGE. **c,** electromicrograph (~105,000x magnification), and **d,** 2D class averages. **e,** Workflow for electron microscopy data processing for the TWIK2:MSP1E1 nanodisc complex in cryoSPARC-3.2[1]. Red arrow indicates the class of particles extracted without Fourier cropping after the initial cleanup. These were further subjected to heterogeneous, non-uniform, and local refinement jobs that resulted in the final map at 3.2Å (red box). **f,** Particle distribution plot and **g,** gold-standard Fourier Shell Correlation (FSC) curves for the TWIK2:MSP1E1 nanodisc complex.

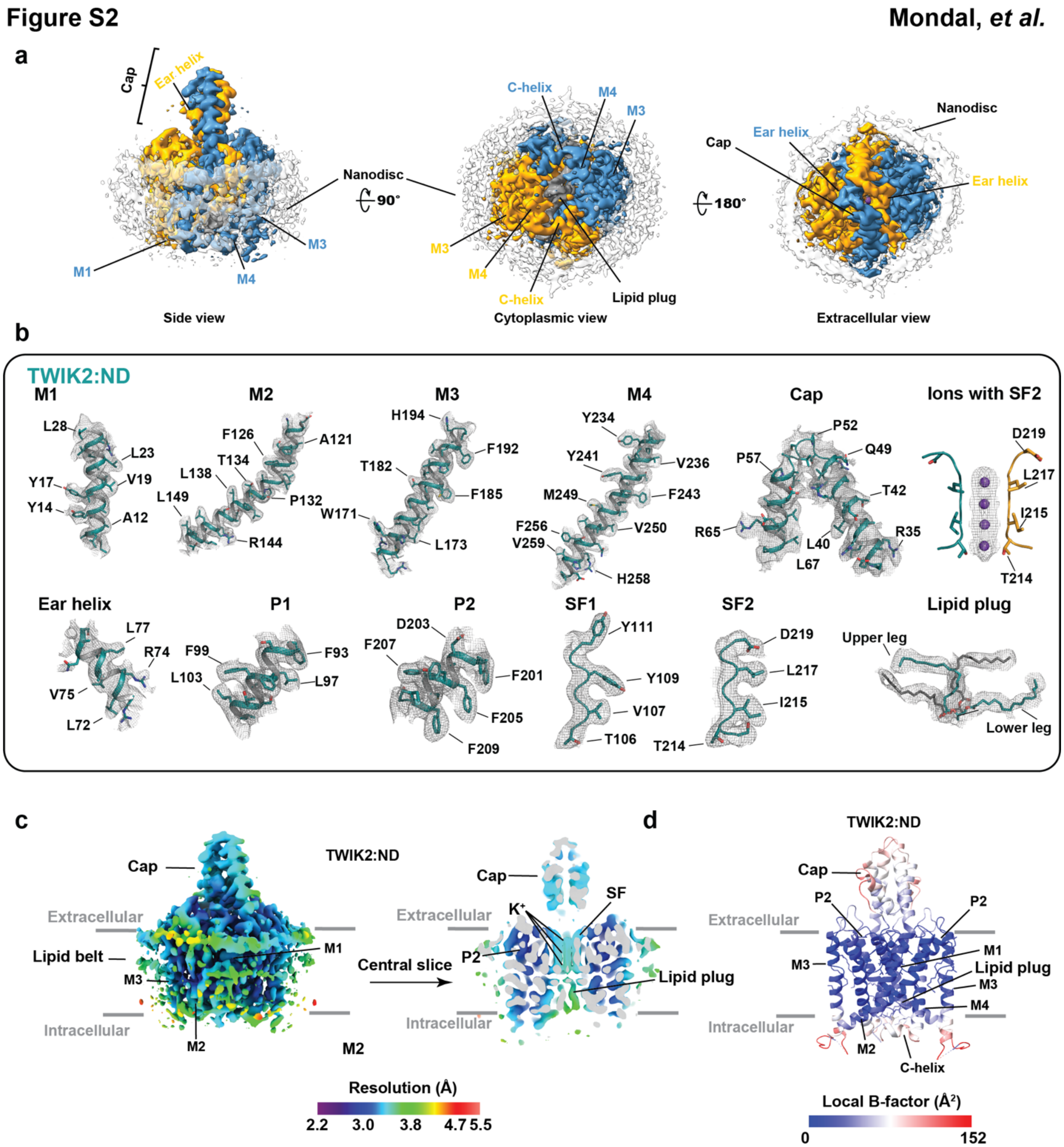

Figure S2 TWIK2:ND cryo-EM map and model quality a, CryoEM map of the TWIK2:MSP1E1 complex. TWIK2 subunits are colored blue and yellow. Lipid plug is colored grey. Nanodisc is clear. Locations of select channel elements are indicated. **b,** Electron microscopy maps for indicated TWIK2 elements. Select residues are indicated. Channel elements are deep teal and bright orange. Potassium ions are purple. Lipid plug is deep teal and grey. Maps are rendered at 2-4σ. **c,** TWIK2:ND local resolution showing a central slice through the channel. Select channel elements and lipid belts are labeled. **d,** TWIK2:ND local B-factor.

Figure S3 **Mondal, *et al.***

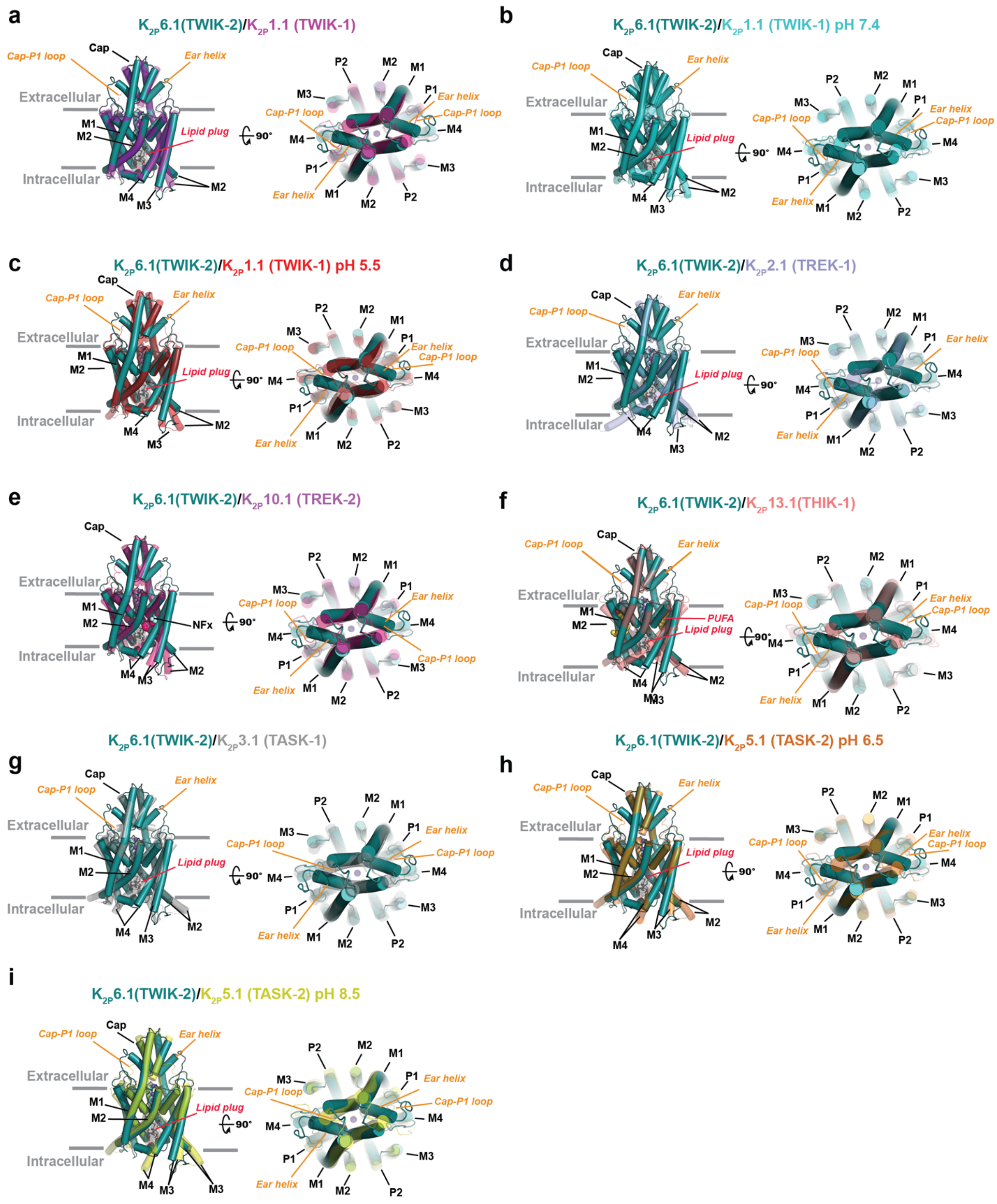

Figure S3 K2P6.1 (TWIK-2) structure comparisons. Superpositions of K2P6.1 (TWIK-2) (deep teal) with: **a,** K2P1.1 (TWIK-1) (magenta) (PDB:3UKM)[2] $RMSD_{C\alpha}$ = 0.817 Å); **b,** K2P1.1

(TWIK-1) pH 7.4 (cyan) (PDB:7SK0) [3](RMSD$_{C\alpha}$ = 1.016 Å) **c,** K$_{2P}$1.1 (TWIK-1) pH 5.5 (red) (PDB:7SK1) [3] (RMSD$_{C\alpha}$ = 1.089 Å); **d,** K$_{2P}$2.1 (TREK-1) (light blue) (PDB: 6CQ6) [4](RMSD$_{C\alpha}$ = 1.021 Å); **e,** K$_{2P}$10.1(TREK-2):norfluoxetine (Nfx) complex (hot pink) (PDB:4XDK) (RMSD$_{C\alpha}$ = 1.05 Å) [5]; **f,** K$_{2P}$13.1(THIK-1) (deep salmon) (PDB:9BSN) [6](RMSD$_{C\alpha}$ = 0.996); **g,** K$_{2P}$3.1(TASK-1) (grey) (PDB:6RV2) (RMSD$_{C\alpha}$ = 1.051 Å); **h,** K$_{2P}$5.1(TASK-2) pH 6.5 (orange) (PDB:6WLV) [7] (RMSD$_{C\alpha}$ = 1.071); and **i,** K$_{2P}$5.1(TASK-2) pH 8.5 (yellow) (PDB:6WM0) [7] (RMSD$_{C\alpha}$ = 0.978). Each panel shows side (left) and extracellular (right) views. Grey bars denote the membrane.

Figure S4 **Mondal, *et al.***

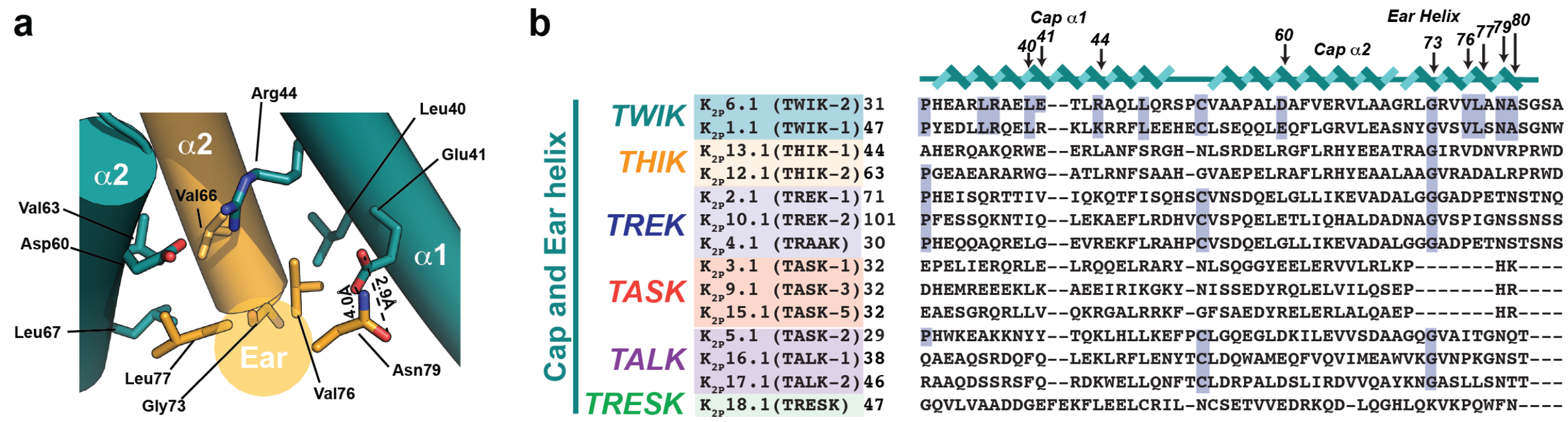

Figure S4 K_{2P}6.1 (TWIK-2) Ear helix interactions and sequence comparison. a, Local environment showing K_{2P}6.1 (TWIK-2) Ear helix-Cap interactions. K_{2P}6.1 (TWIK-2) subunits (deep teal and bright orange) and select residues are indicated. Dashes lines indicate hydrogen bond interactions. **b,** Sequence comparison of Cap and Ear helix from K_{2P}6.1 (TWIK-2) with other K_{2P}s. Numbers indicate residues shown in 'a'. Conservation is shown in blue. Sequences in 'b' are for human: K_{2P}6.1(TWIK-2) (GENBANK 4758624); K_{2P} 1.1(TWIK-1) (GENBANK 4504847); K_{2P}13.1(THIK-1) (GENBANK 16306555); K_{2P}12.1(THIK-2) (GENBANK 11545761); K_{2P}2.1(TREK-1) (GENBANK 14589851); K_{2P}10.1(TREK-2) (GENBANK 20143944); K_{2P}4.1(TRAAK) (GENBANK 15718767); K_{2P}3.1(TASK-1) (GENBANK 4504849; K_{2P}9.1 (TASK-3) (GENBANK 542133161); K_{2P}15.1(TASK-5) (GENBANK 333440483); K_{2P} 5.1(TASK-2) (GENBANK 4504851); K_{2P}16.1(TALK-1) (GENBANK 14149764); K_{2P}17.1(TALK-2) (GENBANK 17025230); and K_{2P}18.1(TRESK) (GENBANK 32469495).

Figure S5 **Mondal, *et al.***

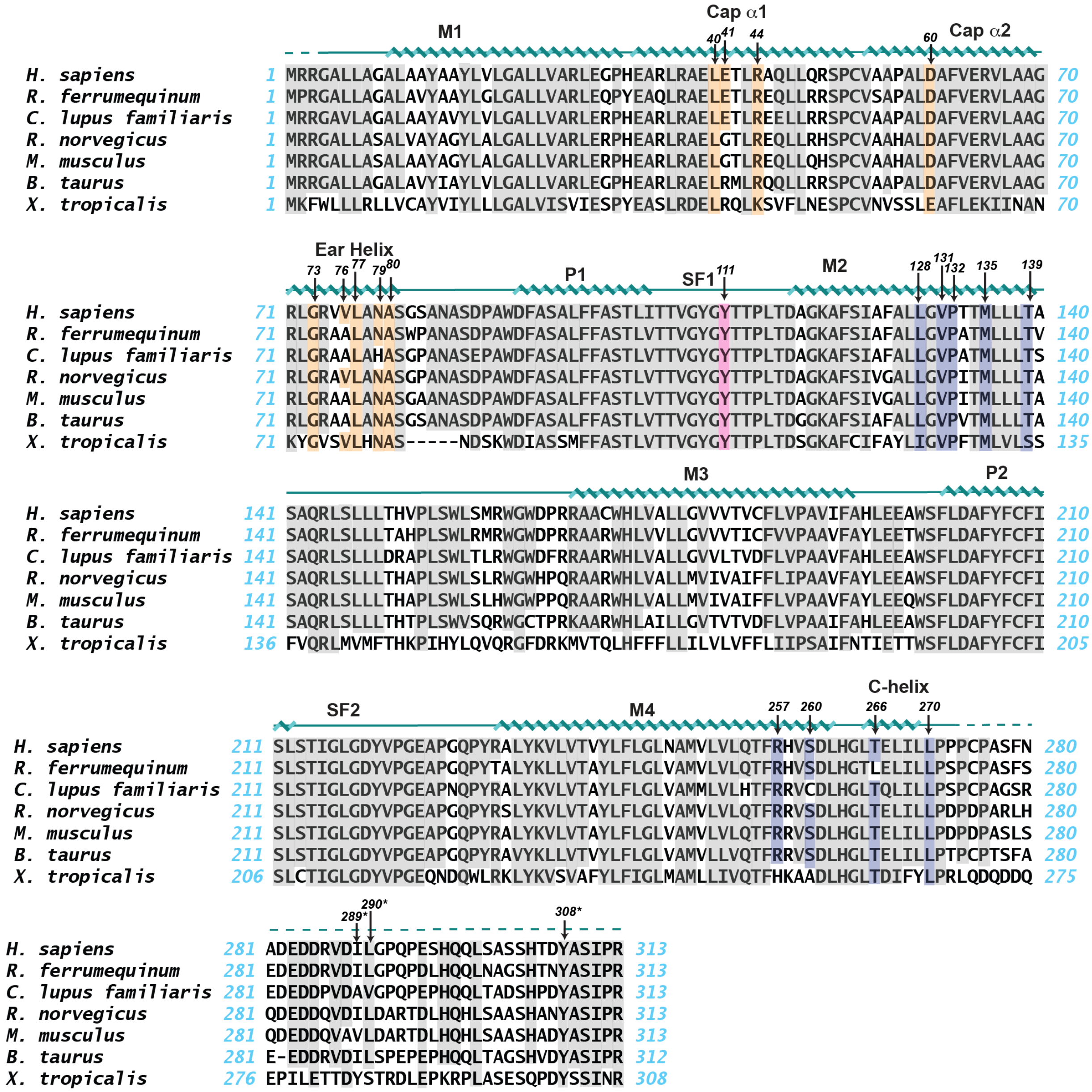

Figure S5 $K_{2P}6.1$ (TWIK-2) sequence conservation. Sequence alignment of *H. sapiens* $K_{2P}6.1$(TWIK-2) (GENBANK 4758624**)** with homologs from the greater horseshoe bat (*R. ferrumequinum*) (GENBANK 117034693), domestic dog (*C. lupus familiaris*) (GENBANK 484531), rat (*R. norvegicus*) (GENBANK 116491), mouse (*M. musculus*) (GENBANK 75766694), cattle (B. taurus) (GENBANK 785418), and Western clawed frog (*X. tropicalis*) (GENBANK 100145504). Cap and Ear helix interaction sites (orange), Tyr111 (magenta), and plug lipid contact sites (blue) are indicated. Asterisk indicates sites of I289A/L290A/Y308 retention mutations. Dashed lines indicate residues lacking defined density in the TWIK2:ND structure.

Figure S6 **Mondal, et al.**

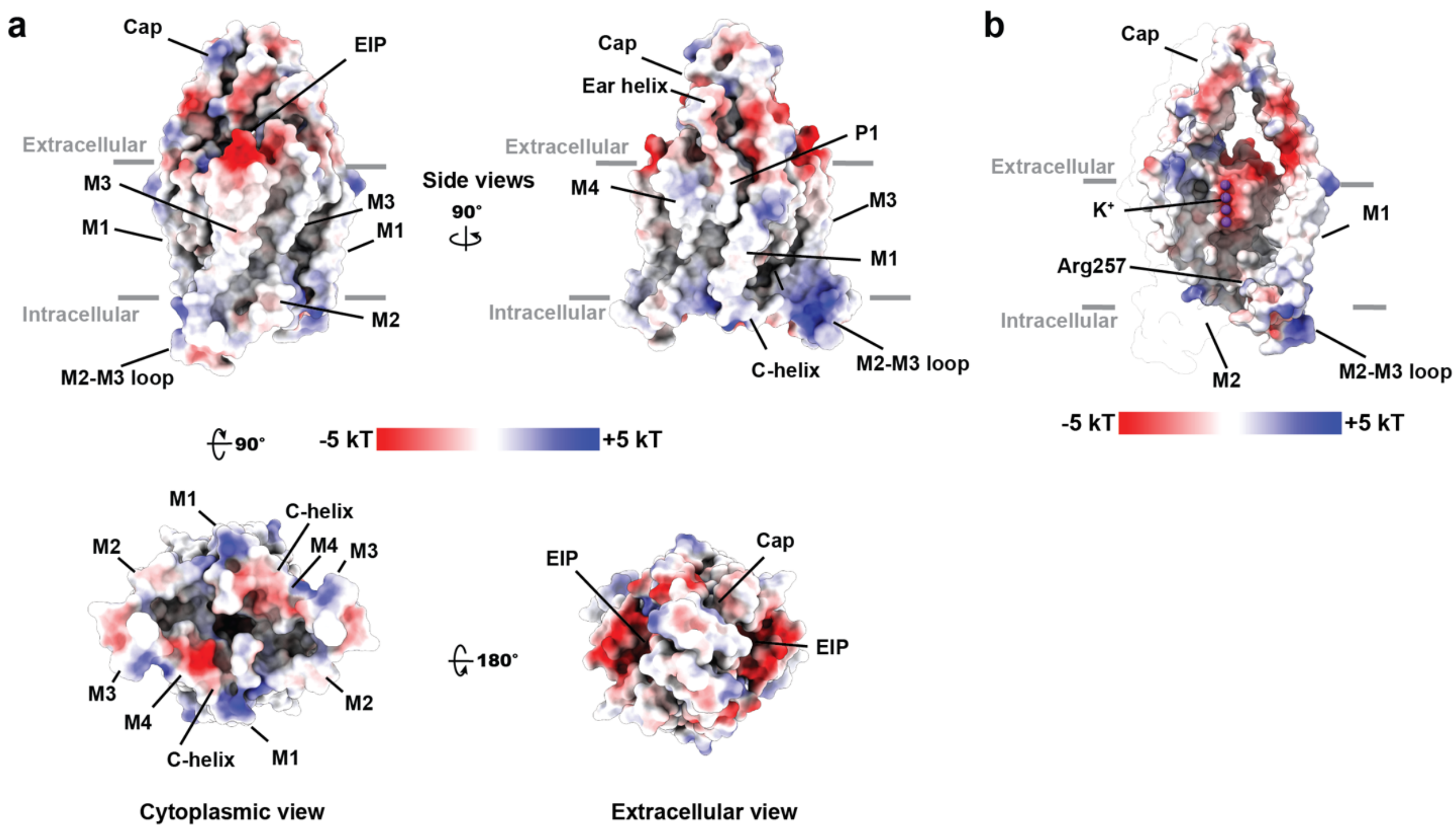

Figure S6 K2P6.1 (TWIK-2) electrostatic surface potentials. a, Electrostatic surface potentials calculated using APBS [8]. **b,** Slice through the center of K2P6.1 (TWIK-2) showing the inner cavity. Selectivity filter ions are shown as purple spheres. Select channel elements are labeled. Grey bars denote the membrane.

8 June 2026

Figure S7 **Mondal, et al.**

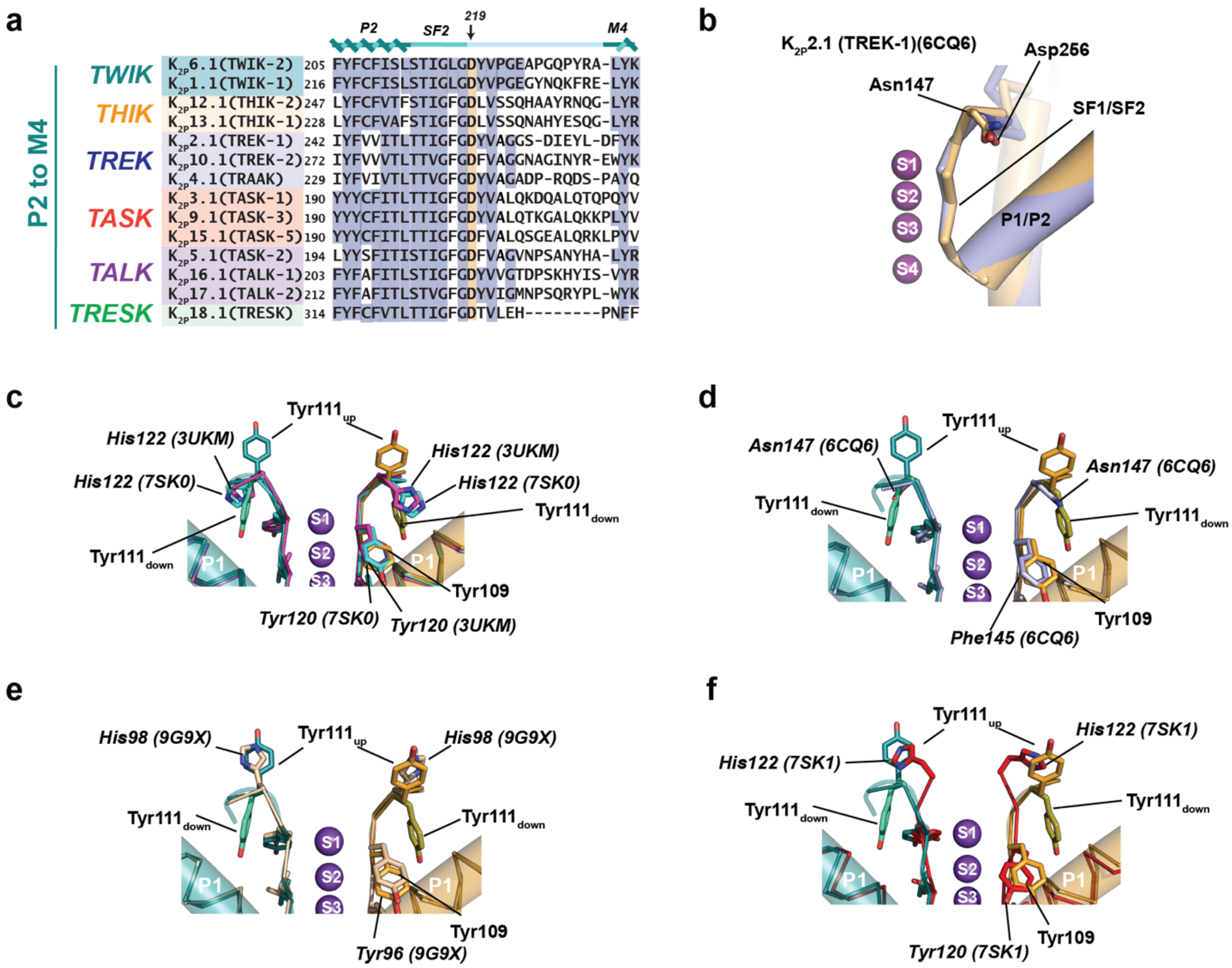

Figure S7 K_{2P}6.1 (TWIK-2) selectivity filter comparisons. a, Sequence comparison of P2 through initial segment of M4 from K_{2P}6.1 (TWIK-2) with other K_{2P}s. Site corresponding to Y111 in SF1 is labeled and indicated in orange. Conservation is shown in blue. SF2-M4 linker is denoted in light blue. **b,** Superposition of SF1 (light orange) and SF2 (slate) regions from K_{2P}2.1(TREK-1) (PDB: 6CQ6) [4] showing the shared conformation of the Asn147 and Asp226 sites corresponding to K_{2P}6.1 (TWIK-2) Tyr111. **c-f,** Superposition of P1-SF1 from K_{2P}6.1 (TWIK-2) with **c,** K_{2P}1.1(TWIK-1) (magenta) (PDB:3UKM)[2] and K_{2P}1.1 (TWIK-1) pH 7.4 (cyan) (PDB:7SK0) [3]; **d,** K_{2P}2.1(TREK-1) (PDB: 6CQ6) [4]; **e,** K_{2P}3.1 (TASK-1) (wheat) pH 7.5 (PDB:9G9X) [9]; **f,** K_{2P}1.1(TWIK-1) pH 5.5 (red) (PDB:7SK1) [3]. In 'c-f' the Tyr111 'up' and 'down' conformations are indicated. Sequences in 'a' are for human: K_{2P}6.1(TWIK-2) (GENBANK 4758624); K_{2P} 1.1(TWIK-1) (GENBANK 4504847); K_{2P}13.1(THIK-1) (GENBANK 16306555); K_{2P}12.1(THIK-2) (GENBANK 11545761); K_{2P}2.1(TREK-1) (GENBANK 14589851); K_{2P}10.1(TREK-2) (GENBANK 20143944); K_{2P}4.1(TRAAK) (GENBANK 15718767); K_{2P}3.1(TASK-1) (GENBANK 4504849); K_{2P}9.1 (TASK-3) (GENBANK 542133161);

K_{2P}15.1(TASK-5) (GENBANK 333440483); K_{2P} 5.1(TASK-2) (GENBANK 4504851); K_{2P}16.1(TALK-1) (GENBANK 14149764); K_{2P}17.1(TALK-2) (GENBANK 17025230); and K_{2P}18.1(TRESK) (GENBANK 32469495).

Figure S8 **Mondal, *et al.***

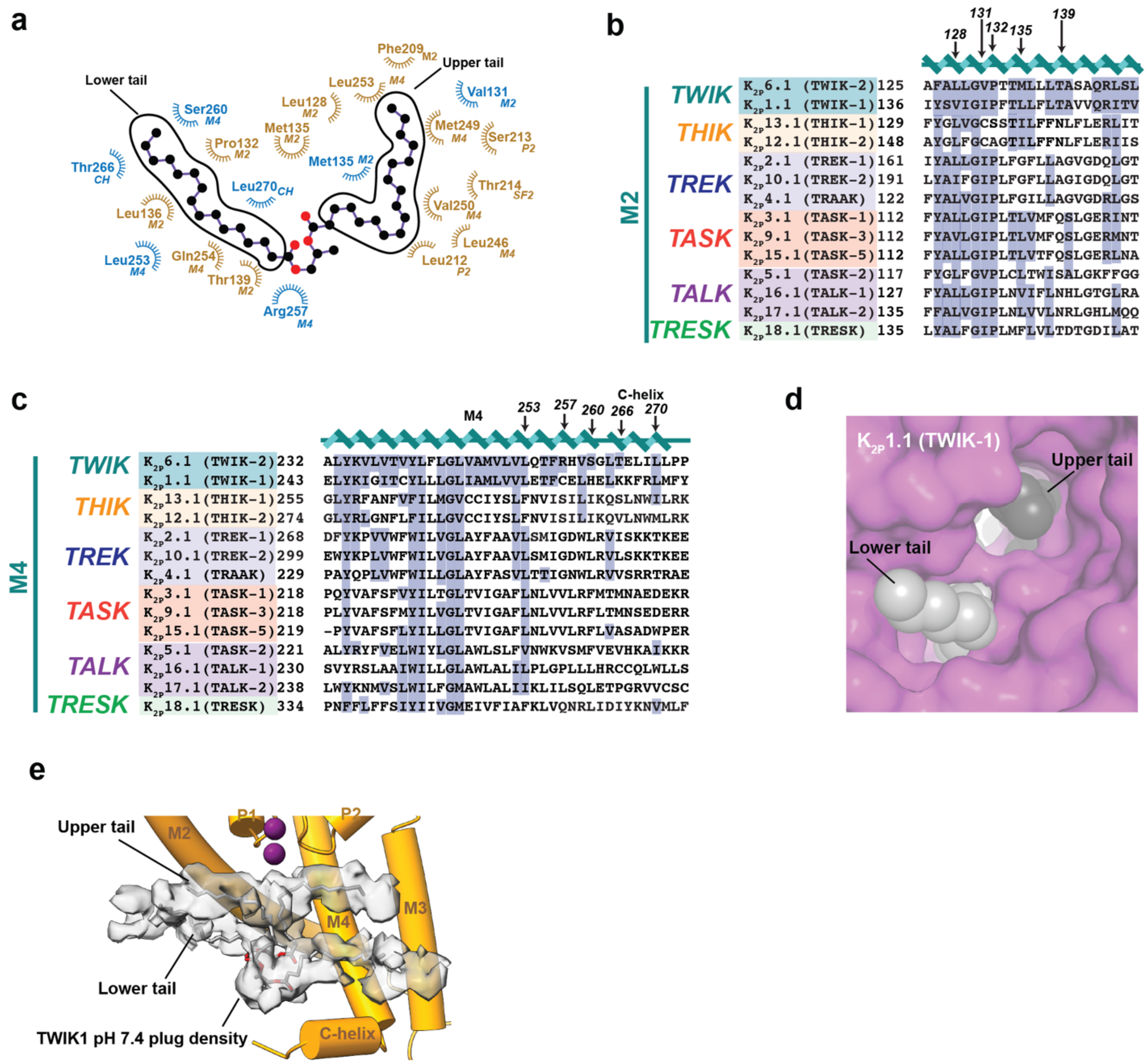

Figure S8 K2P6.1 (TWIK-2):plug lipid interactions. a, Ligplot[10] diagram showing plug lipid interactions (≤ 5Å). K2P6.1 (TWIK-2) chains are indicated in blue and olive. Lipid upper and lower tail elements are indicated. Channel elements M2, M4, selectivity filter 2 (SF2), and C-helix (CH) are indicated. **b,** and **c,** Sequence comparison of K2P6.1 (TWIK-2) **b,** M2 and **c,** M4 and C-helix regions with other with other K2Ps. Labels indicated residues that interact with the plug lipid. Conservation is indicated in blue. **d,** View of the K2P1.1(TWIK-1) (PDB:3UKM) [2] surface from the perspective of the bilayer. Lipids (grey and black) are from the TWIK2 structure. View is similar to Fig. 2d. Sequences in 'b' and 'c' are for human: K2P6.1(TWIK-2) (GENBANK 4758624); K2P 1.1(TWIK-1) (GENBANK 4504847); K2P13.1(THIK-1) (GENBANK 16306555); K2P12.1(THIK-2) (GENBANK 11545761); K2P2.1(TREK-1) (GENBANK 14589851); K2P10.1(TREK-2) (GENBANK 20143944); K2P4.1(TRAAK) (GENBANK 15718767);

$K_{2P}3.1$(TASK-1) (GENBANK 4504849); $K_{2P}9.1$ (TASK-3) (GENBANK 542133161); $K_{2P}15.1$(TASK-5) (GENBANK 333440483); $K_{2P}5.1$(TASK-2) (GENBANK 333440483); $K_{2P}16.1$(TALK-1) (GENBANK 14149764); $K_{2P}17.1$(TALK-2) (GENBANK 17025230); and $K_{2P}18.1$(TRESK) (GENBANK 32469495). **e,** Lipid plug density (1.8σ) from $K_{2P}1.1$ (TWIK-1) at pH 7.4 (EMDB 25168)[3]. Model shows a single $K_{2P}6.1$(TWIK-2) subunit and lipid plug (grey)

Figure S9 **Mondal, et al.**

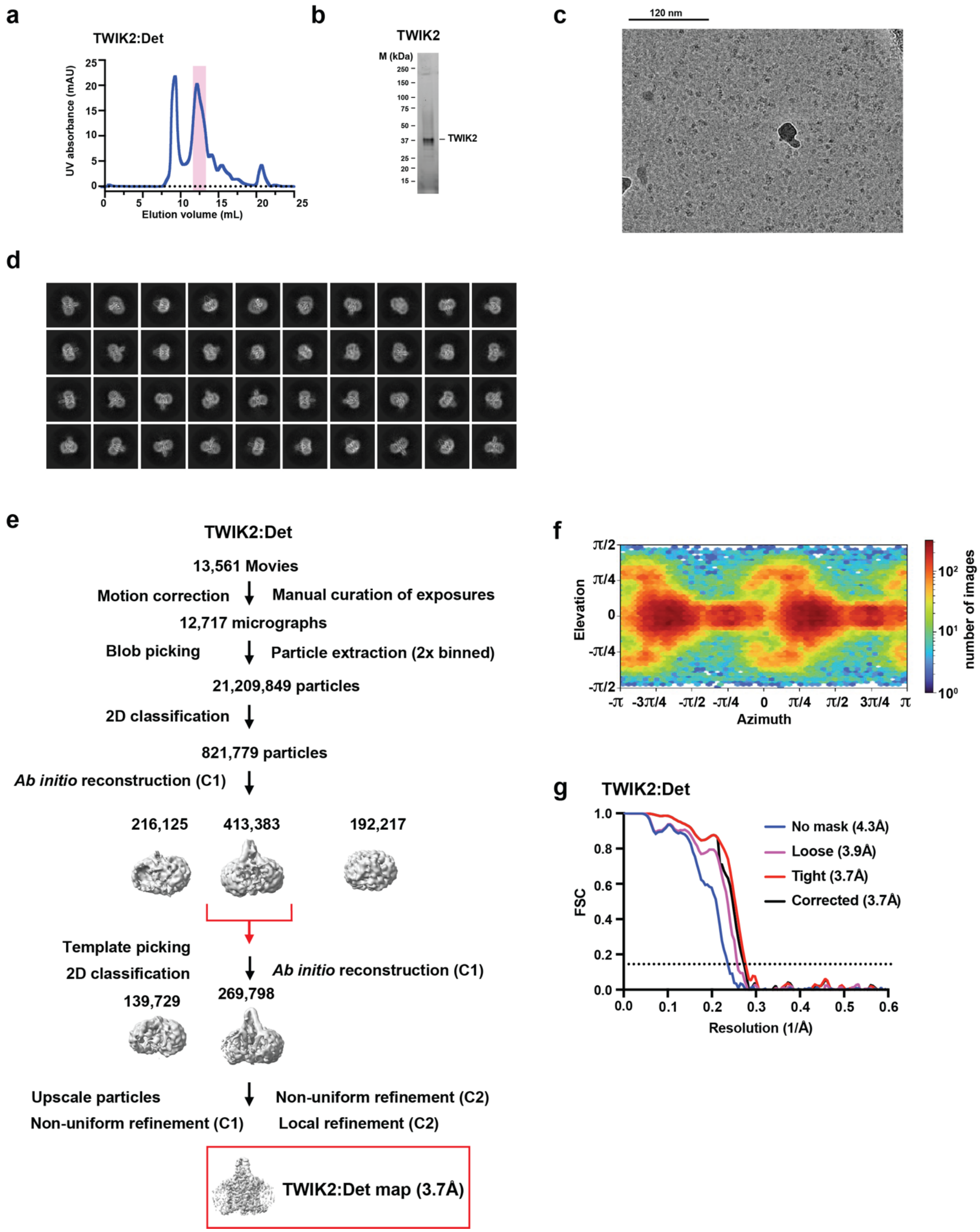

Figure S9 Cryo-EM analysis of TWIK2 in detergent. Exemplar **a,** SEC (Superose 6 Increase 10/300 GL) for TWIK2 in β-docecylmaltoside (TWIK2:Det), **b,** peak fraction SDS-PAGE. **c,** electromicrograph (~105,000x magnification), and **d**, 2D class averages. **e,** Workflow for electron microscopy data processing for TWIK2:Det in cryoSPARC-3.2[1]. Red arrow indicates the class of particles extracted without Fourier cropping after the initial cleanup. These were further subjected to heterogeneous, non-uniform, and local refinement jobs that resulted in the final map at 3.7Å (red box). **f,** Particle distribution plot and **g,** gold-standard Fourier Shell Correlation (FSC) curves for TWIK2:Det.

Figure S10 **Mondal, *et al.***

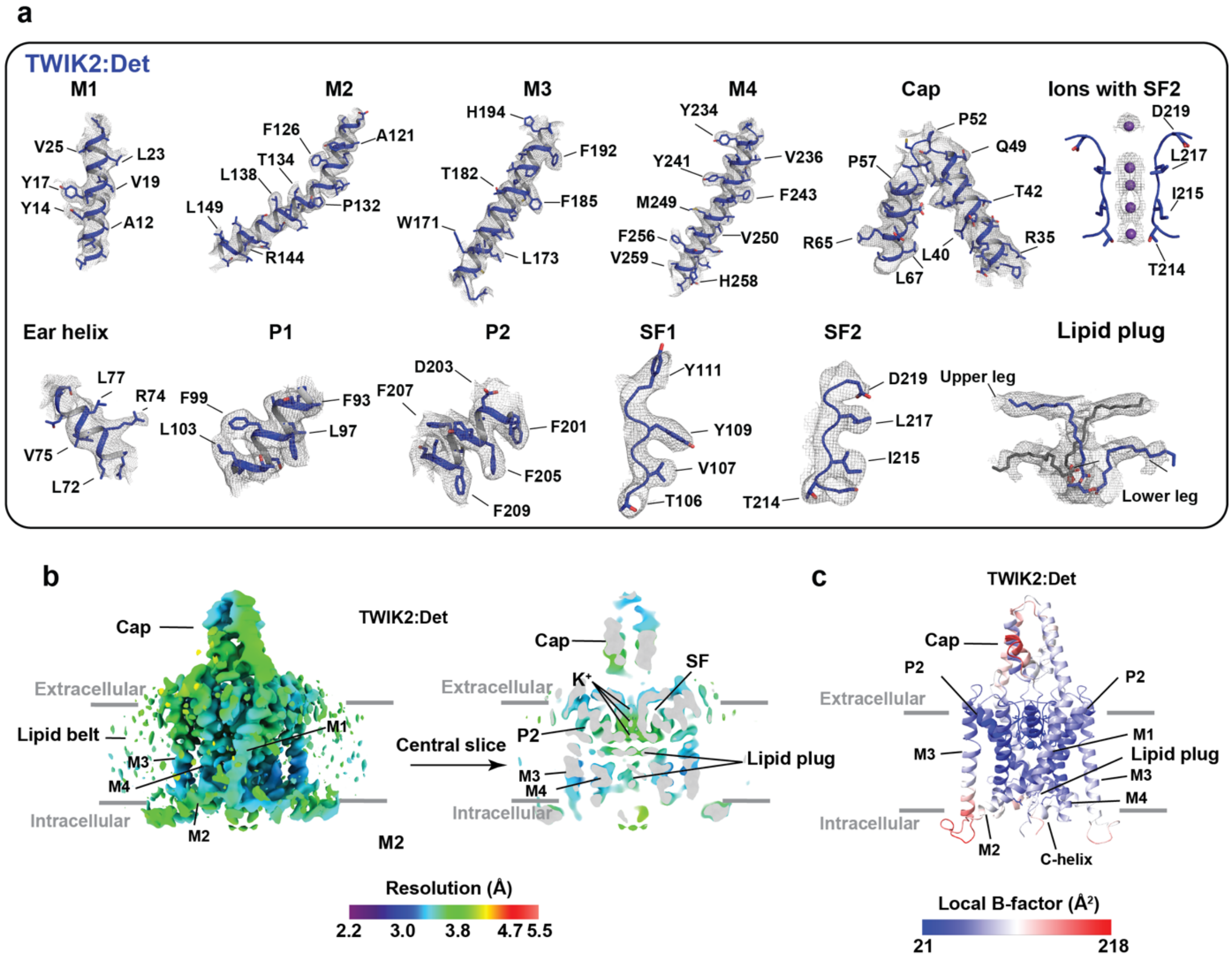

Figure S10 TWIK2:Det cryo-EM map and model quality a, Electron microscopy maps for indicated TWIK2 elements. Select residues are indicated. Channel elements are blue. Potassium ions are purple. Lipid plug is blue and grey. Maps are rendered at 3-4σ. **b,** TWIK2:Det local resolution showing a central slice through the channel. Select channel elements and lipid belt s are labeled. **c,** TWIK2:Det local B-factor.

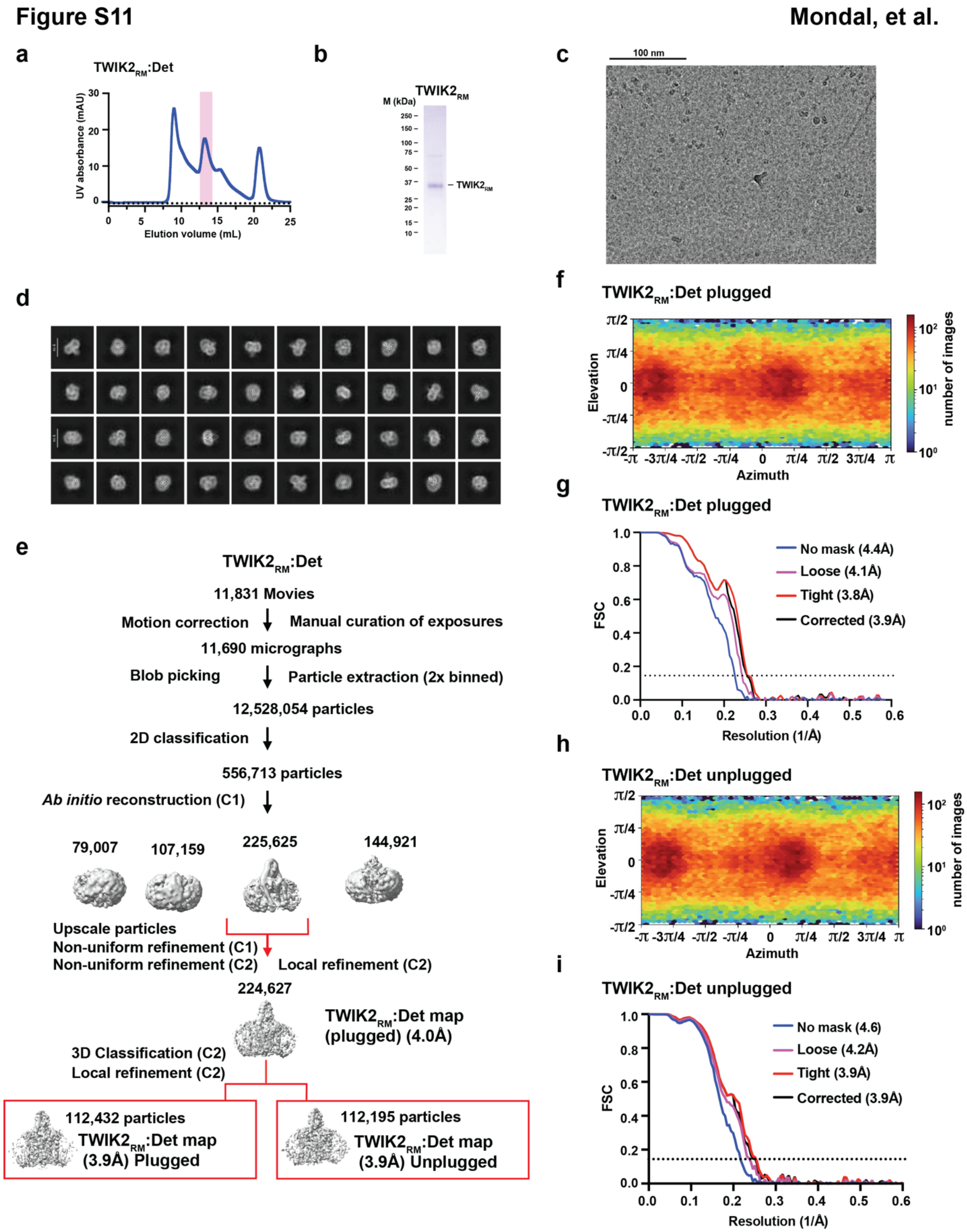

Figure S11 Cryo-EM analysis of TWIK2$_{RM}$ in detergent. Exemplar **a,** SEC (Superose 6 Increase 10/300 GL) for TWIK2$_{RM}$ in N-dodecyl-β–D-maltoside (TWIK2:Det), **b,** peak fraction

SDS-PAGE. **c,** electromicrograph (~105,000x magnification), and **d**, 2D class averages. **e,** Workflow for electron microscopy data processing for TWIK2$_{RM}$:Det in cryoSPARC-3.2[1]. Red arrow indicates the class of particles extracted without Fourier cropping after the initial cleanup. Non-uniform refinement, followed by 3D classification was performed using the extracted particles yielding 6 different classes that were then combined, following manual inspection, into two classes representing the plugged and unplugged channel. These particle-sets were further subjected to Local Refinement to obtain the final maps. **f,** Particle distribution plot and **g,** gold-standard Fourier Shell Correlation (FSC) curves for TWIK2$_{RM}$ plugged. **h,** Particle distribution plot and **i,** gold-standard Fourier Shell Correlation (FSC) curves for TWIK2$_{RM}$ unplugged.

Figure S12 **Mondal, *et al.***

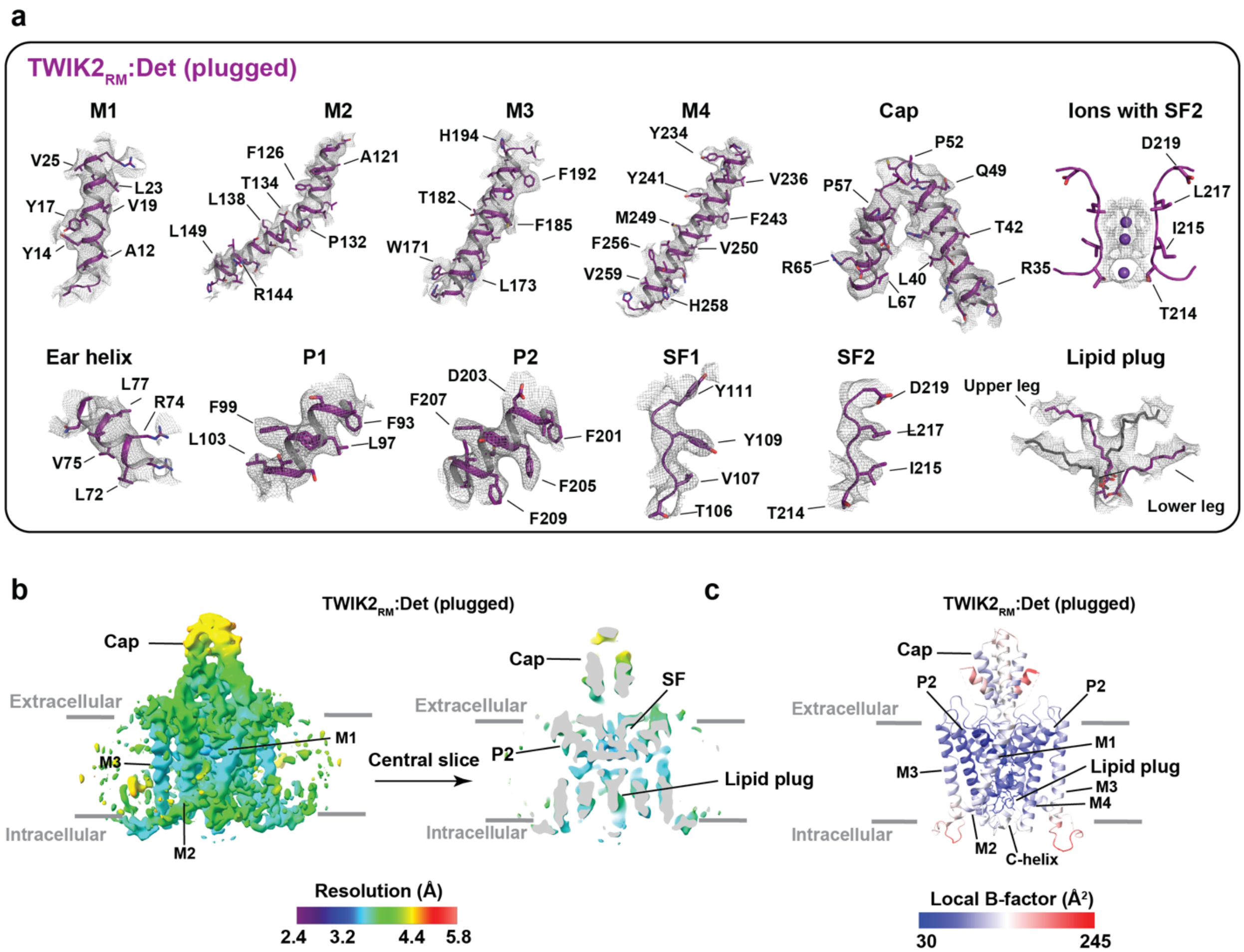

Figure S12 TWIK2$_{RM}$:Det (plugged) cryo-EM map and model quality a, Electron microscopy maps for indicated TWIK2$_{RM}$ (plugged) elements. Select residues are indicated. Channel elements are dark purple. Potassium ions are purple. Lipid plug is dark purple and grey. Maps are rendered at 3-4σ. **b,** TWIK2:Det$_{RM}$ (plugged) local resolution showing a central slice through the channel. Select channel elements and lipid belt s are labeled. **c,** TWIK2:Det$_{RM}$ (plugged) local B-factor.

Figure S13 **Mondal, *et al.***

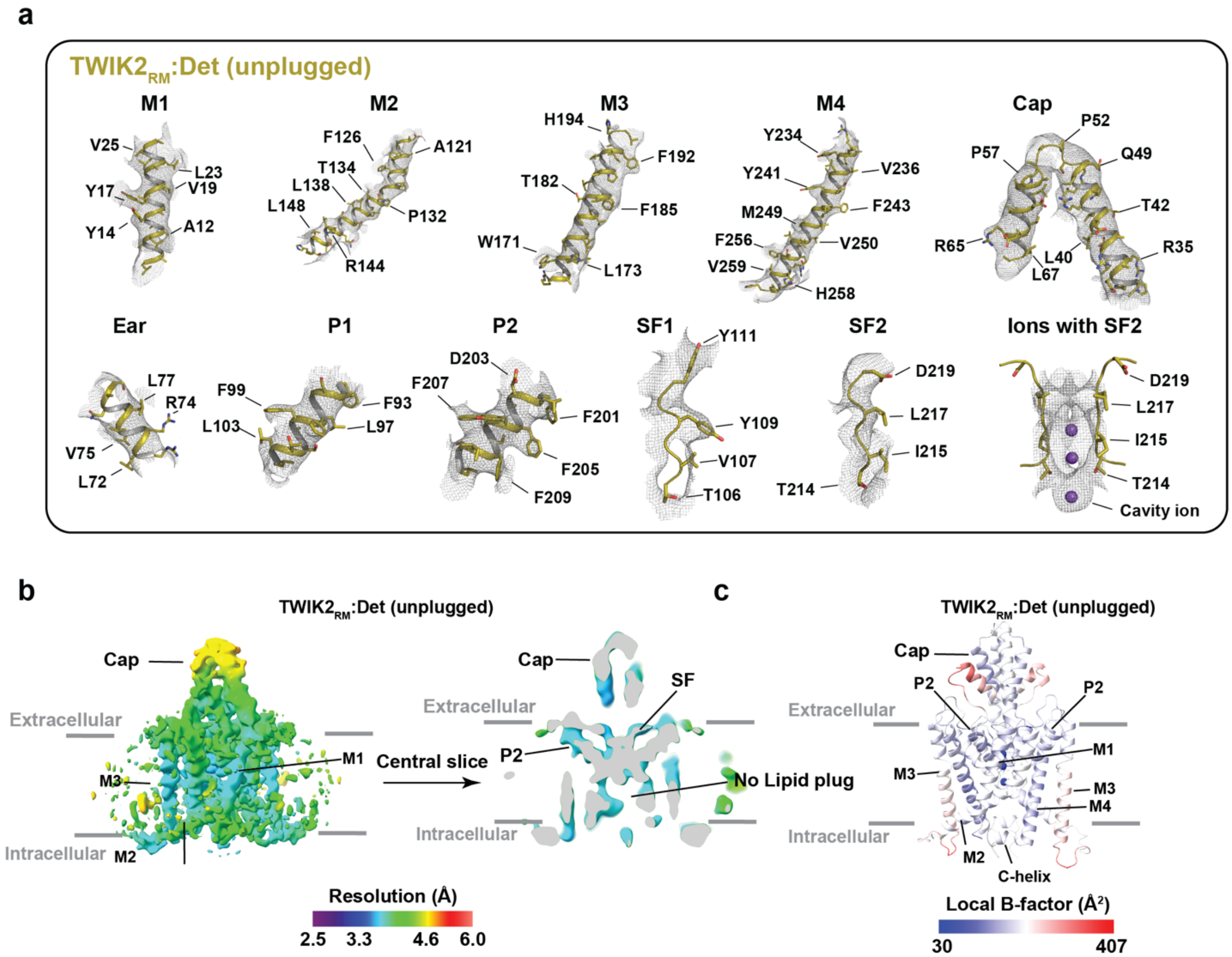

Figure S13 TWIK2RM:Det (unplugged) cryo-EM map and model quality a, Electron microscopy maps for indicated TWIK2RM (unplugged)elements. Select residues are indicated. Channel elements are olive. Potassium ions are purple. Lipid plug is dark purple and grey. Maps are rendered at 3-4σ. **b,** TWIK2RM:Det (unplugged) local resolution showing a central slice through the channel. Select channel elements are labeled. **c,** TWIK2:DetRM (unplugged) local B-factor.

Figure S14 **Mondal, *et al.***

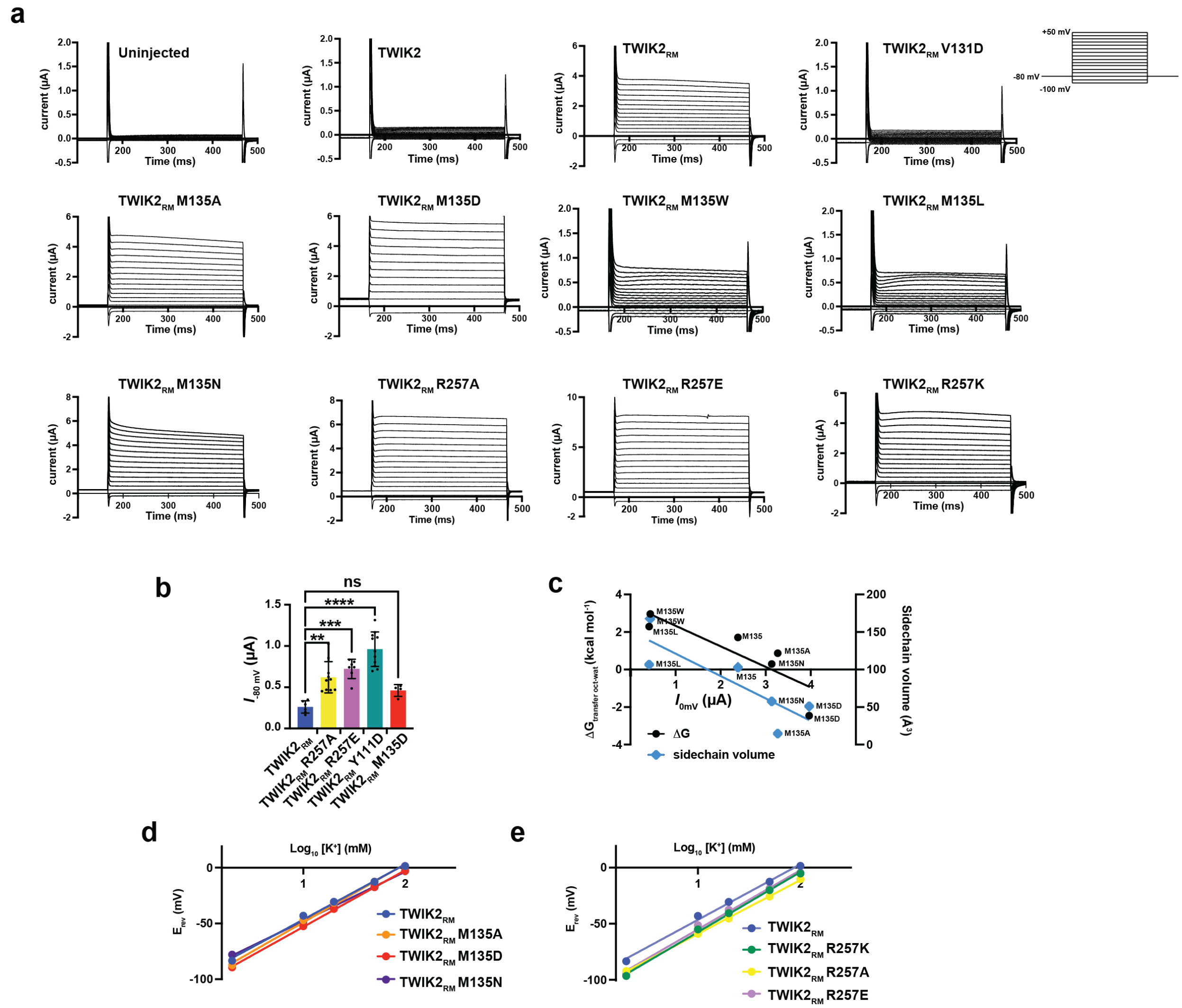

Figure S14 Functional studies of TWIK2 central cavity mutants. a, Exemplar TEVC recordings from *Xenopus* oocytes. Currents for uninjected (control), TWIK2, $TWIK2_{RM}$ and $TWIK2_{RM}$ mutants V131D, M135A, M135D, M135W, M135L, M135N, R257A, R257E, and R257K. Inset shows protocol. **b,** Comparison of basal currents at -80 mV for the indicated channels. **** $p < 0.0001$, *** $p < 0.001$, ** $p < 0.01$, * p = 0.01 -0.05, p>0.05 (ns). Statistical analysis was performed using a One-Way ANOVA and multiple comparisons were performed with Tukey's test. Error bars are S.E.M.. **c,** Comparison of water-octanol transfer free energy[12] (black circles) and sidechain volume (blue diamonds)[13] with basal current at 0 mV. **d,** and **e,** Potassium selectivity for **d,** $TWIK2_{RM}$ (blue), $TWIK2_{RM}$ M135A (orange), $TWIK2_{RM}$ M135D (red), and $TWIK2_{RM}$ M135N (dark purple) and **e,** Potassium selectivity for $TWIK2_{RM}$ (blue), $TWIK2_{RM}$ R257K (green), $TWIK2_{RM}$ R257A (yellow), and $TWIK2_{RM}$ R257E (purple), In 'd' and 'e' lines represent fits to the Nernst equation $E_K = RT/zF \ln[K^+]_{out}/[K^+]_{in}$ assuming /$[K^+]_{in} = 108.6$ mM [11].

Figure S15 **Mondal, *et al.***

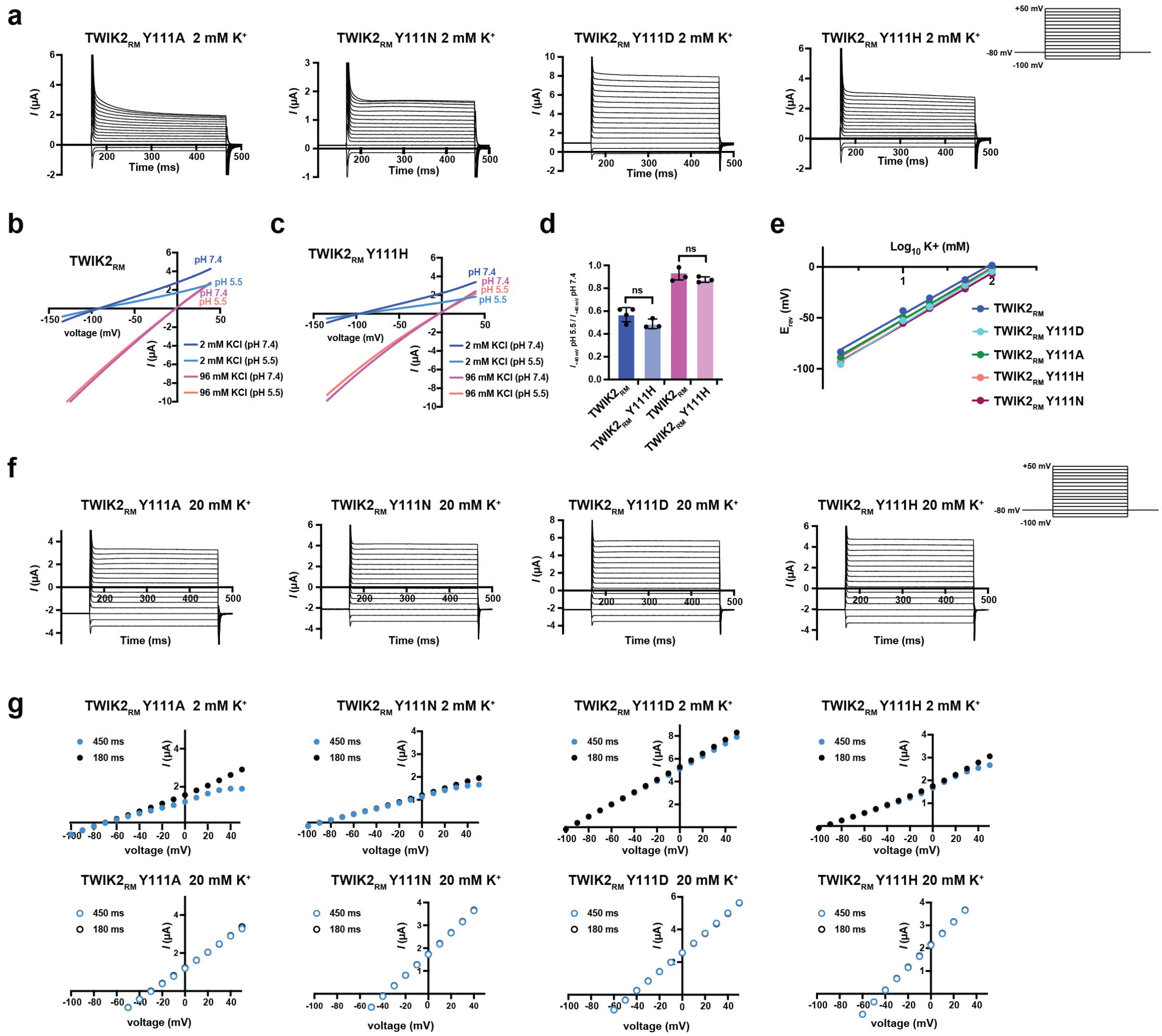

Figure S15 Functional studies of the TWIK2 Tyr111 site. a, Exemplar TEVC recordings from *Xenopus* oocytes in 2 mM $[K^+]_{ext}$ showing currents for $TWIK2_{RM}$ mutants Y111A, Y111N, Y111D, and Y111H. Inset shows protocol. **b,** and **c,** pH effects (pH 7.4 vs. 5.5) on **b,** $TWIK2_{RM}$ and **c,** $TWIK2_{RM}$ Y111H in low external (2 mM) and high external (96 mM) potassium. **d,** ratio of pH responses for $TWIK2_{RM}$ and $TWIK2_{RM}$ Y111H at pH5.5 vs. pH 7.4 in low external (2 mM) (blue) and high external (96 mM) (magenta) potassium. **e,** Potassium selectivity for $TWIK2_{RM}$ (blue) $TWIK2_{RM}$ Y111A (green), $TWIK2_{RM}$ Y111D (light blue), $TWIK2_{RM}$ Y111H (orange), $TWIK2_{RM}$ Y111N (maroon). Lines represent fits to the Nernst equation $E_K = RT/zF \ln[K^+]_{out}/[K^+]_{in}$ assuming $[K^+]_{in}$ = 108.6 mM[11]. **f,** Exemplar TEVC recordings from *Xenopus* oocytes in 20 mM $[K^+]_{ext}$ showing currents for $TWIK2_{RM}$ mutants Y111A, Y111N, Y111D, and Y111H. Inset shows protocol. **g,** I-V relation for $TWIK2_{RM}$ mutants Y111A, Y111N, Y111D, and Y111H at 180 ms and 480 ms in the exemplar protocols for 2 mM $[K^+]_{ext}$ (top row) and 20 mM $[K^+]_{ext}$ (bottom row).

Figure S16 **Mondal, et al.**

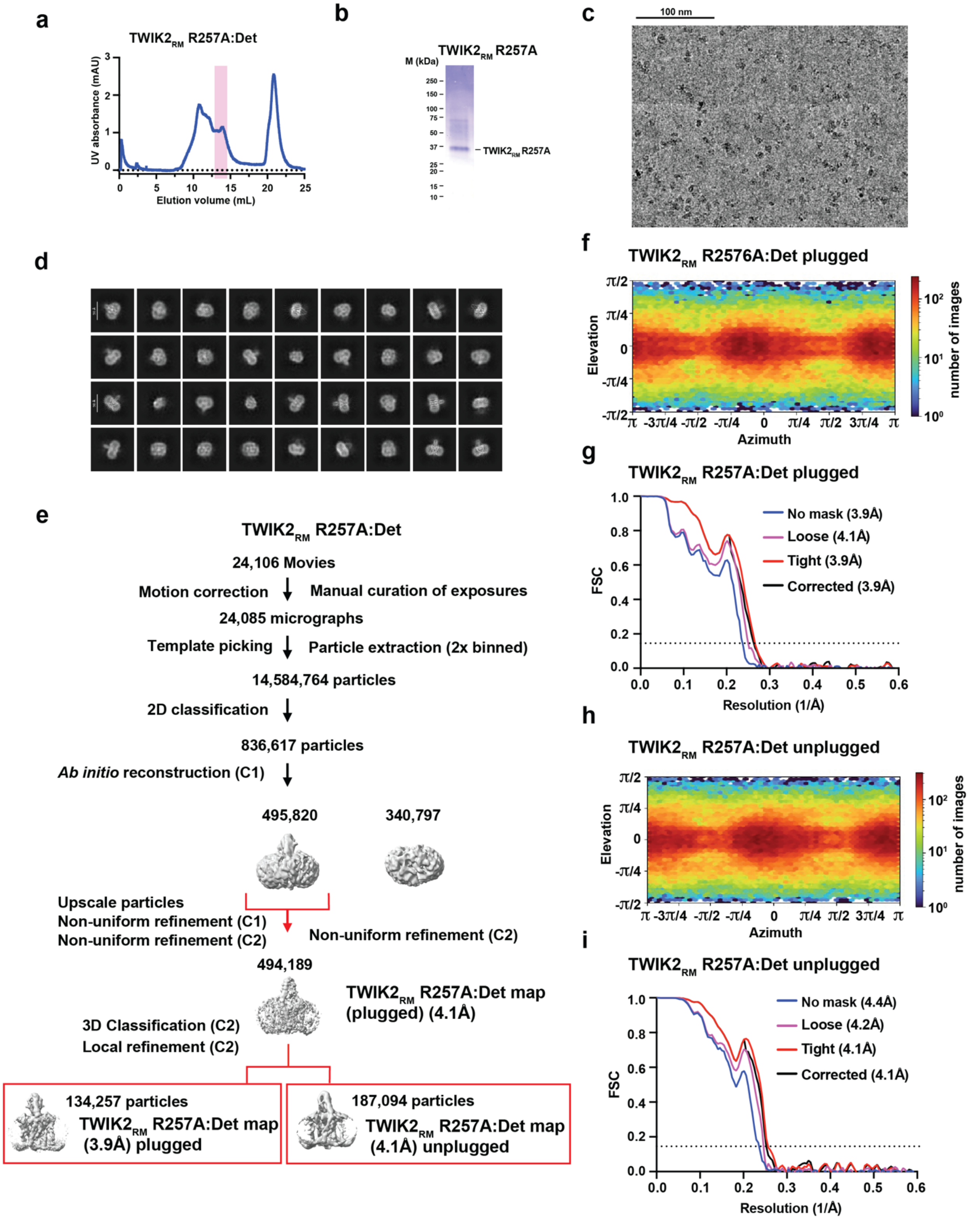

Figure S16 Cryo-EM analysis of TWIK2$_{RM}$ R257A in detergent. Exemplar **a,** SEC (Superose 6 Increase 10/300 GL) for TWIK2$_{RM}$ R257A in N-dodecyl-β−D-maltoside (TWIK2:Det), **b,** peak fraction SDS-PAGE. **c,** electron micrograph (~105,000x magnification), and **d**, 2D class averages. **e,** Workflow for electron microscopy data processing for TWIK2$_{RM}$ R257A:Det in cryoSPARC-3.2[1]. Red arrow indicates the class of particles re-extracted without Fourier cropping after the initial cleanup. Extracted particles were further subjected to non-uniform refinement to generate a consensus map. This refined particle set was used for 3D classification having lipid plug density as focused mask and generated 6 classes, which later combined into two major groups having either the presence or absence of the lipid plug. These particle-sets were further subjected to Local Refinement to obtain the final maps. **f,** Particle distribution plot and **g,** gold-standard Fourier Shell Correlation (FSC) curves for TWIK2$_{RM}$ R257A plugged. **h,** Particle distribution plot and **i,** gold-standard Fourier Shell Correlation (FSC) curves for TWIK2$_{RM}$ R257A unplugged.

Figure S17 **Mondal, *et al.***

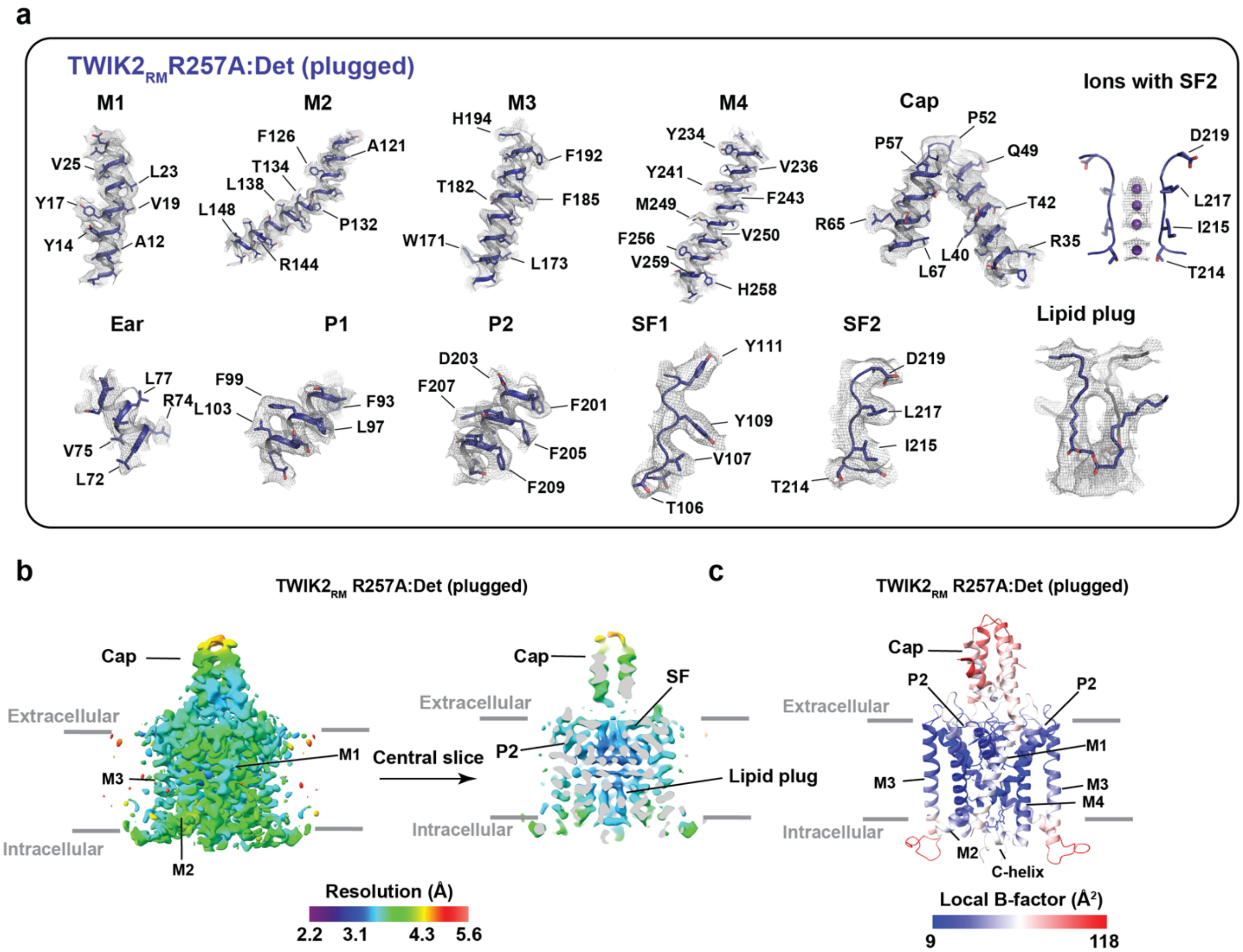

Figure S17 TWIK2$_{RM}$ R257A:Det (plugged) cryo-EM map and model quality a, Electron microscopy maps for indicated TWIK2$_{RM}$ R257A:Det (plugged) elements. Select residues are indicated. Channel elements are dark purple. Potassium ions are purple. Lipid plug is dark purple and grey. Maps are rendered at 3-4σ. **b,** TWIK2$_{RM}$ R257A:Det (plugged) local resolution showing a central slice through the channel. Select channel elements are labeled. **c,** TWIK2$_{RM}$ R257A:Det (plugged) local B-factor.

Figure S18 **Mondal, *et al.***

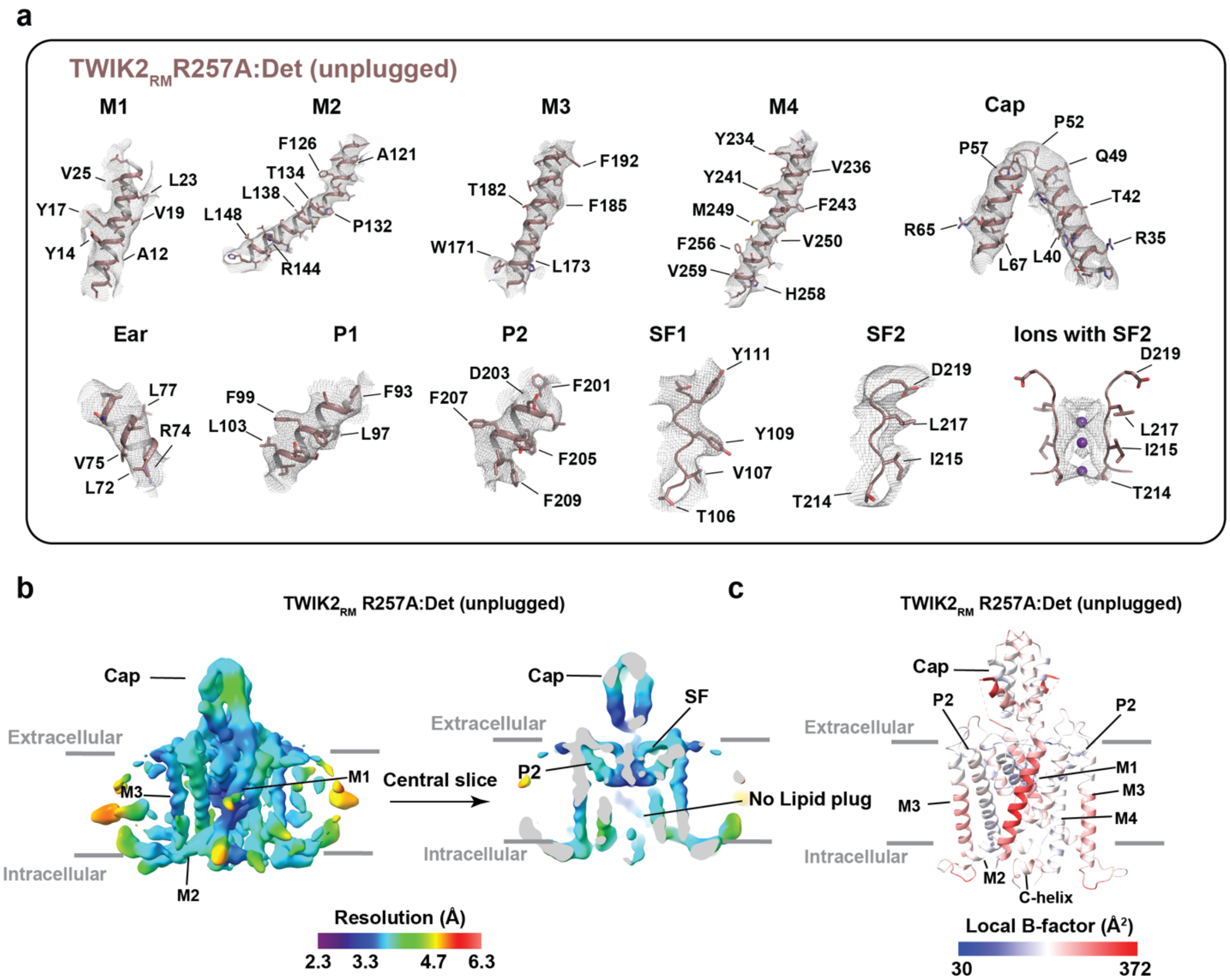

Figure S18 TWIK2RM R257A:Det (unplugged) cryo-EM map and model quality a, Electron microscopy maps for indicated TWIK2RM R257A:Det (unplugged) elements. Select residues are indicated. Channel elements are brown. Potassium ions are purple. Maps are rendered at 3-4σ. **b,** TWIK2RM R257A:Det (unplugged) local resolution showing a central slice through the channel. Select channel elements are labeled. **c,** TWIK2RM R257A:Det (unplugged) local B-factor.

Figure S19 **Mondal, *et al.***

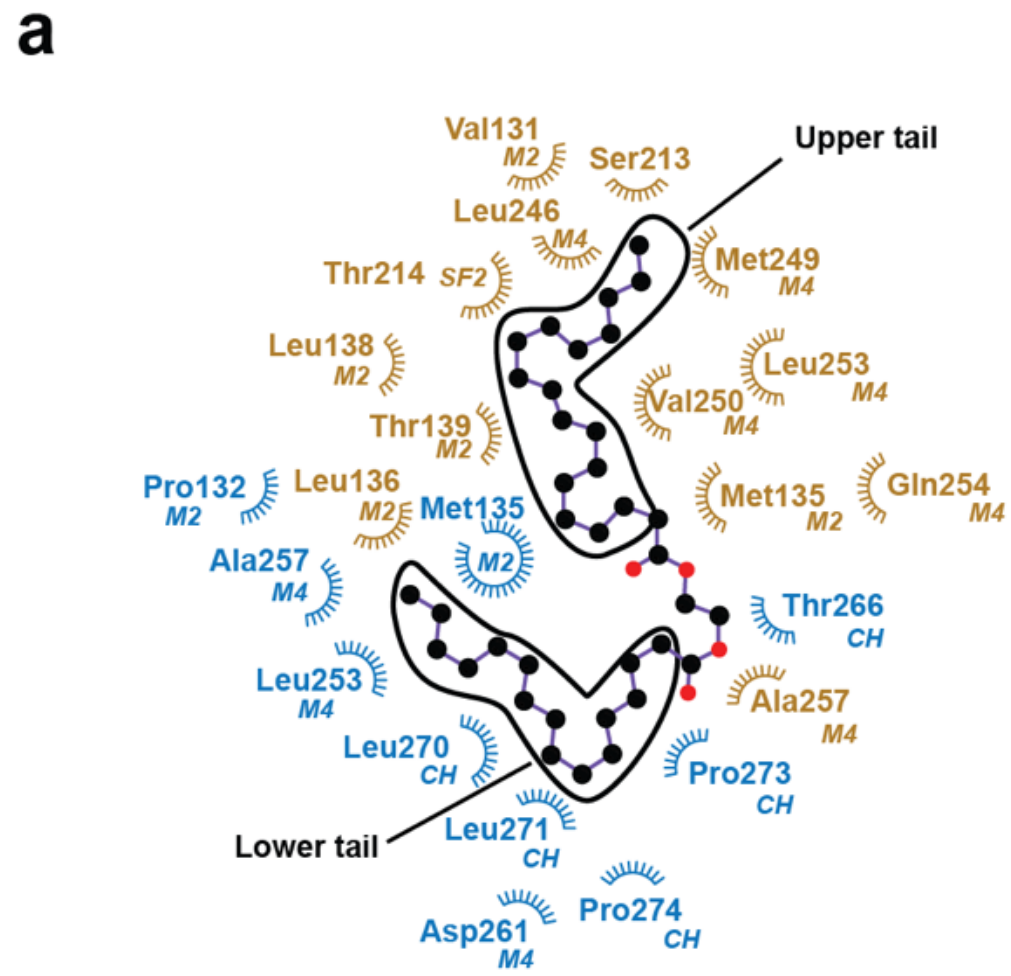

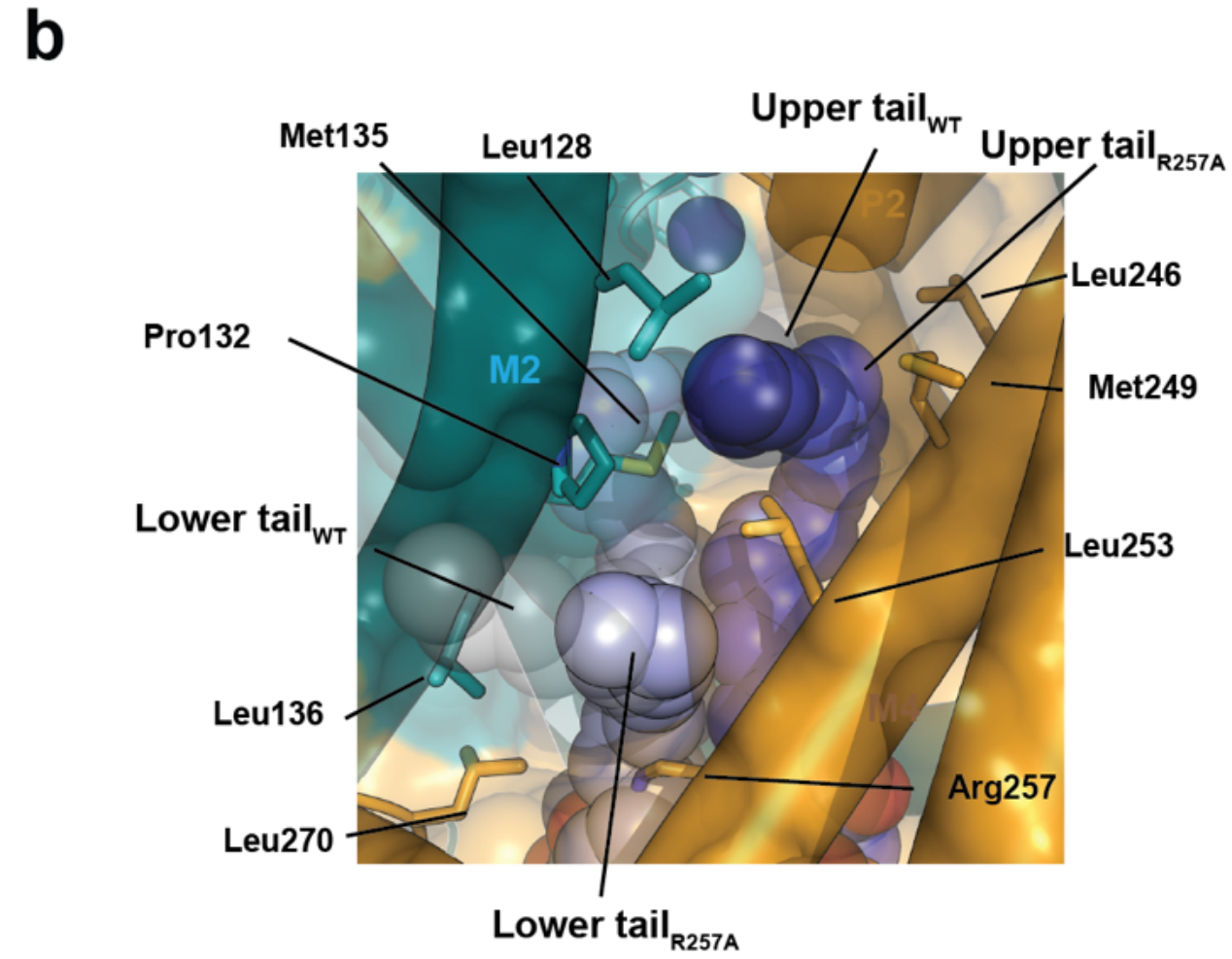

Figure S19 TWIK2$_{RM}$ R257A:plug lipid interactions. a, Ligplot[10] diagram showing plug lipid interactions (≤5Å). TWIK2$_{RM}$ R257A chains are indicated in blue and olive. Lipid upper and lower tail elements are indicated. Channel elements M2, M4, selectivity filter 2 (SF2), and C-helix (CH) are indicated. **b,** View from the center of the bilayer towards the upper and lower tail binding sites. Comparison of the TWIK2$_{RM}$ R257A plug lipids (slate and light blue) with the TWIK2:ND structure. Lipid plug chains are shown in space filling representation. Interacting residues are shown as sticks.

Figure S20 Mondal, *et al.*

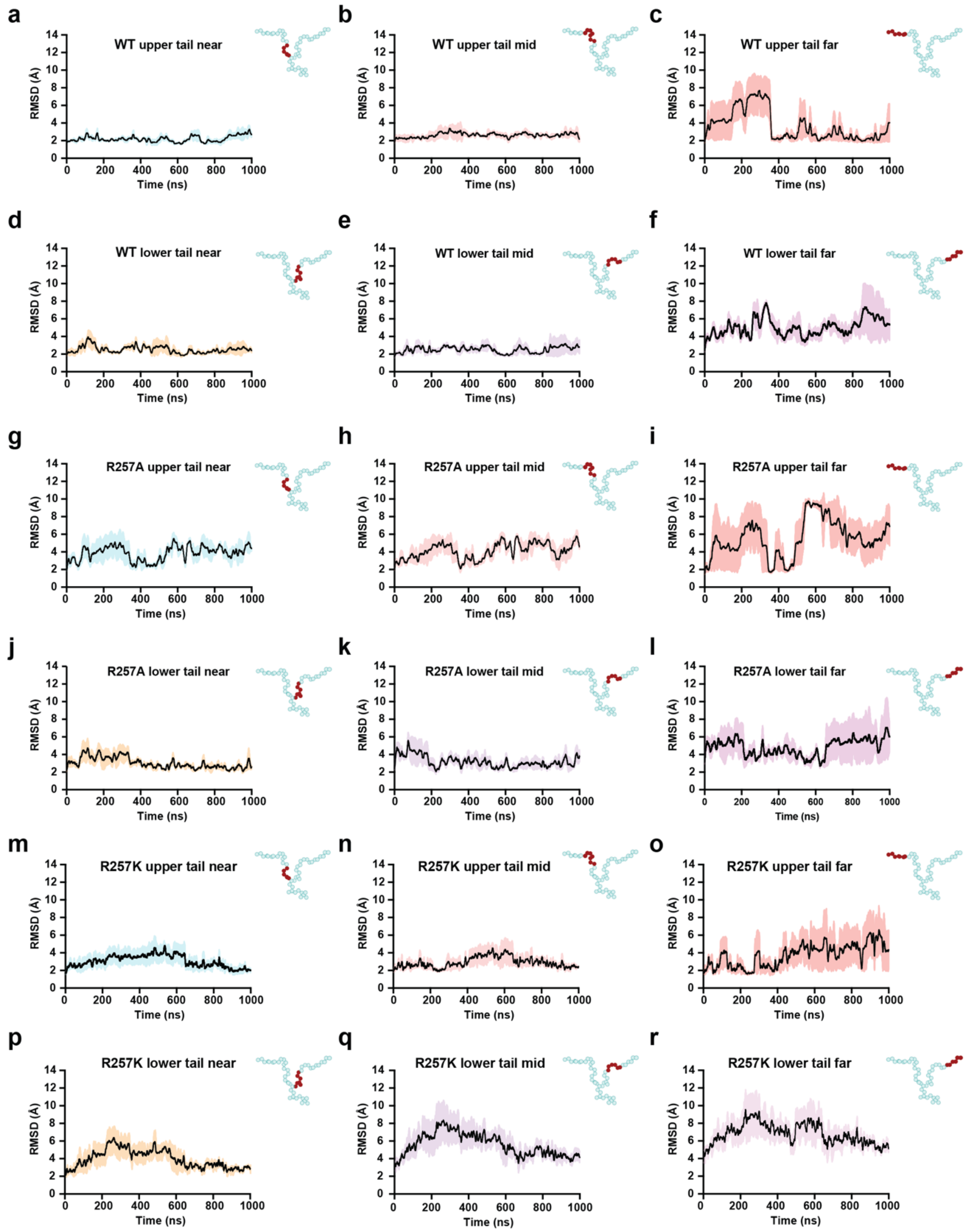

Figure S20 Lipid behavior during simulations highlights the role of Arg257. Time series of RMSD values (smoothed over a 1 ns window) calculated for non-hydrogen atoms of the lipid plug (POPC) over 1 µs molecular dynamics simulations (n=3) for **a–f** TWIK2, **g–l,** TWIK2 R257A, and **m–r,** TWIK2 R257K. Reference is the position of the lipid plug in the TWIK2:ND structure.

Icons depict the corresponding POPC atom clusters used for each RMSD calculation. Shading shows SEM.

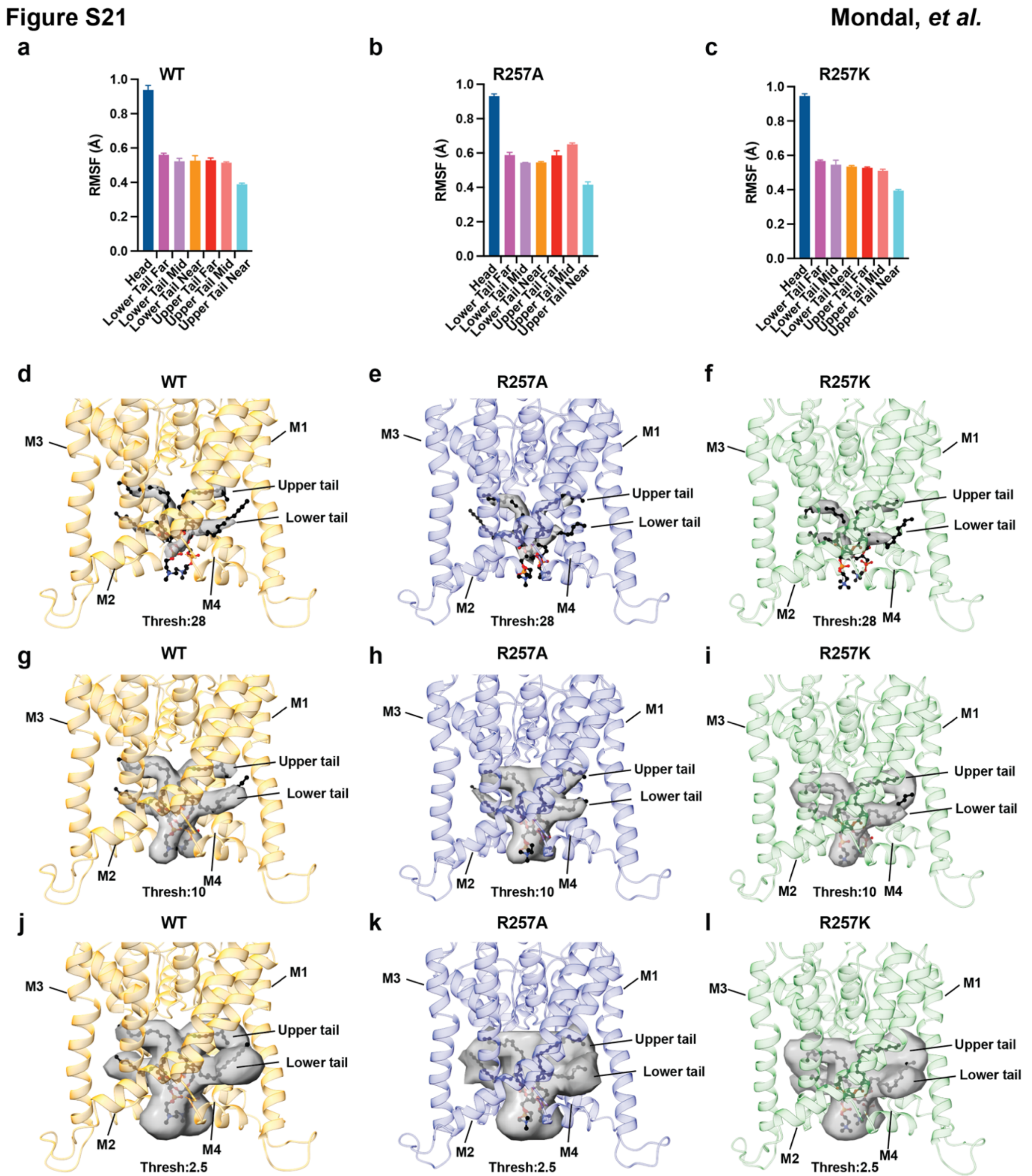

Figure S21 Dynamic behavior of lipid plug during MD simulations. a-c, Root mean square fluctuation (RMSF) of different segments (as shown in Figure S20 inset) of the bound representative POPC plug lipid in TWIK2 (WT), R257K, and R257A (mean ± SEM, n=3 independent 1 µs replicas). **d-l**, Comparison of time-averaged lipid density maps (contoured at different density thresholds in ChimeraX denoted 'Thresh') obtained from 1 µs all-atom MD simulations of TWIK2 (WT), R257K and R257A mutants.

Figure S22 **Mondal, et al.**

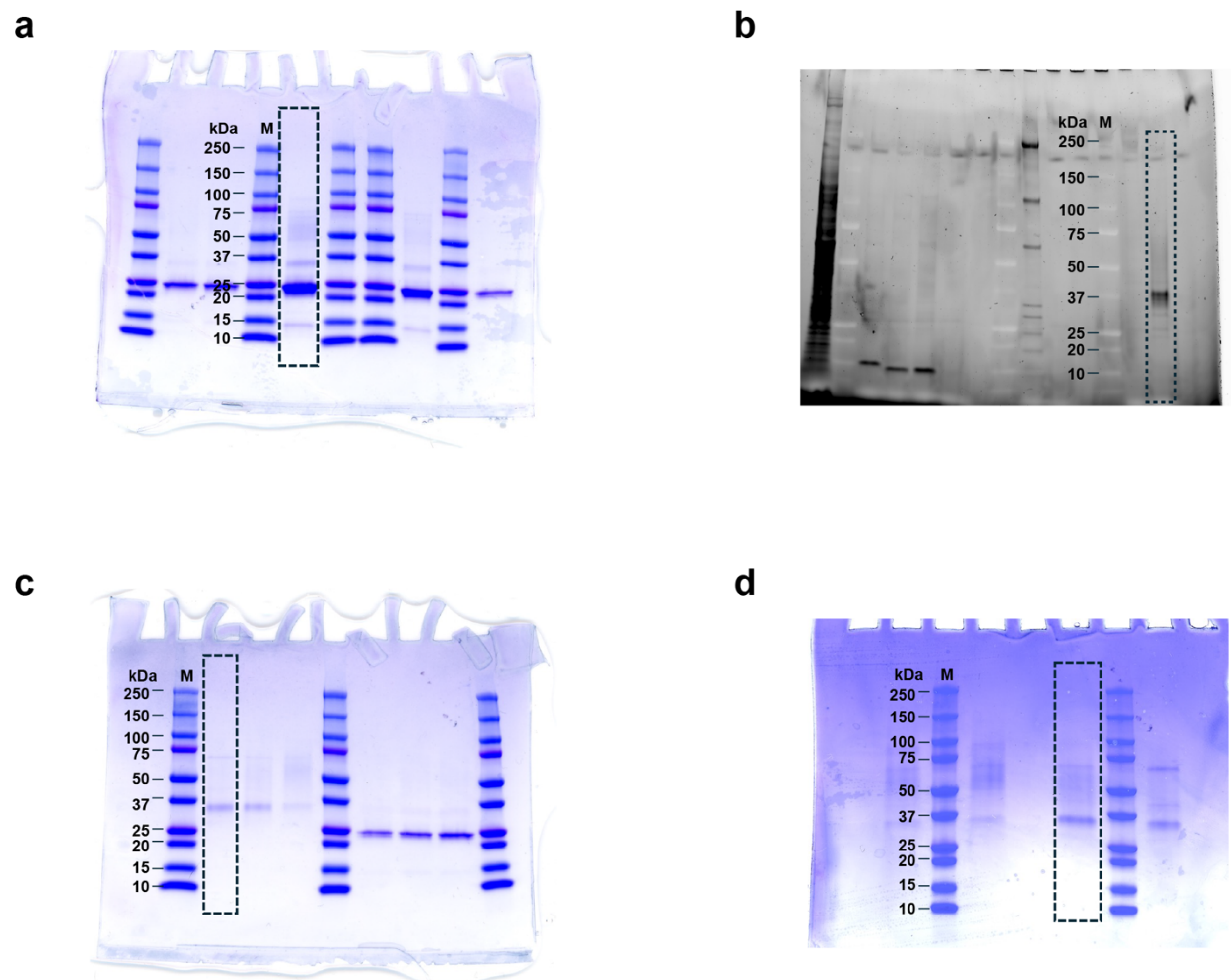

Figure S22 Uncropped gels for Figures a, S1b, **b,** S9b, **c,** S11b, and **d,** S16b.

Supplementary Table S1 Molecular dynamics

	TWIK2	TWIK2 R257A	TWIK2 R257K
Box dimensions (Å)	116.8x116.8x121.6	116.7x116.7x122.2	116.1x116.1x123.5
Total number of atoms	170,225	170,228	170,239
Total number of waters	3,7423	3,7434	3,7429
Salt concentration	150 mM KCl	150 mM KCl	150 mM KCl
Lipid composition	1-palmitoyl-2-oleoyl-sn-glycero-3-phosphocholine (POPC)	1-palmitoyl-2-oleoyl-sn-glycero-3-phosphocholine (POPC)	1-palmitoyl-2-oleoyl-sn-glycero-3-phosphocholine (POPC)
Total number of lipids	370	370	370

Supplementary Table S2 Molecular Dynamic Simulation Checklist

**Reliability and reproducibility checklist for molecular dynamics simulations**<br>***All boxes must be marked YES by acceptance unless "Response not needed if No".**	**Yes**	**No**	**Response (Please state where this information can be found in the text)**
**1. Convergence of simulations and analysis**			
1a. Is an evaluation presented in the text to show that the property being measured has equilibrated in the simulations (*e.g.* time-course analysis)?	☒	☐	Molecular dynamics simulations paragraph, under Materials and Methods section.
1b. Then, is it described in the text how simulations are split into equilibration and production runs and how much data were analyzed from production runs?	☒	☐	Molecular dynamics simulations paragraph, under Materials and Methods section.
1c. Are there at least 3 simulations per simulation condition with statistical analysis?	☒	☐	Molecular dynamics simulations paragraph, under Materials and Methods section.
1d. Is evidence provided in the text that the simulation results presented are independent of initial configuration?	☒	☐	Molecular dynamics simulations paragraph, under Materials and Methods section.
**2. Connection to experiments**			
2a. Are calculations provided that can connect to experiments (*e.g.* loss or gain in function from mutagenesis, binding assays, NMR chemical shifts, J-couplings, SAXS curves, interaction distances or FRET distances, structure factors, diffusion coefficients, bulk modulus and other mechanical properties, *etc*.)?	☒	☐	Results and discussion section.
**3. Method choice**			
3a. Do simulations contain membranes, membrane proteins, intrinsically disordered proteins, glycans, nucleic acids, polymers, or cryptic ligand binding?	☒	☐	Simulations contain membrane proteins and membranes.
3b. Is it described in the text whether the accuracy of the chosen model(s) is sufficient to address the question(s) under investigation (e.g. all-atom vs. coarse-grained models, fixed charge vs. polarizable force fields, implicit vs. explicit solvent or membrane, force field and water model, etc.)?	☒	☐	Results and discussion section
3c. Is the timescale of the event(s) under investigation beyond the brute-force MD simulation timescale in this study that enhanced sampling methods are needed?	☐	☒	

	If **YES**, are the parameters and convergence criteria for the enhanced sampling method clearly stated?	☐	☐	
	If **NO,** is the evidence provided in the text?	☒	☐	Results and discussion section
**4. Code and reproducibility**				
4a. Is a table provided describing the system setup that includes simulation box dimensions, total number of atoms, total number of water molecules, salt concentration, lipid composition (number of molecules and type)?		☒	☐	Molecular dynamics simulations paragraph, under Materials and Methods section.<br>Also provided as a table in supplementary document
4b. Is it described in the text what simulation and analysis software and which versions are used?		☒	☐	Molecular dynamics simulations paragraph, under Materials and Methods section.
4c. Are other parameters for the system setup described in the text, such as protonation state, type of structural restraints if applied, nonbonded cutoff, thermostat and barostat, etc.?		☒	☐	Molecular dynamics simulations paragraph, under Materials and Methods section.
4d. Are initial coordinate and simulation input files and a coordinate file of the final output provided as supplementary files or in a public repository?		☒	☐	Available in public repository
4e. Is there custom code or custom force field parameters?		☐	☒	Response not needed if **No**
	If **YES**, are they provided as supplementary files or in a public repository?	☐	☐	

References

1. Punjani, A., Rubinstein, J.L., Fleet, D.J., and Brubaker, M.A. (2017). cryoSPARC: algorithms for rapid unsupervised cryo-EM structure determination. Nature methods *14*, 290-296. 10.1038/nmeth.4169.
2. Miller, A.N., and Long, S.B. (2012). Crystal structure of the human two-pore domain potassium channel K2P1. Science *335*, 432-436. 335/6067/432 [pii] 10.1126/science.1213274.
3. Turney, T.S., Li, V., and Brohawn, S.G. (2022). Structural Basis for pH-gating of the K(+) channel TWIK1 at the selectivity filter. Nat Commun *13*, 3232. 10.1038/s41467-022-30853-z.
4. Lolicato, M., Arrigoni, C., Mori, T., Sekioka, Y., Bryant, C., Clark, K.A., and Minor, D.L., Jr. (2017). K2P2.1 (TREK-1)-activator complexes reveal a cryptic selectivity filter binding site. Nature *547*, 364-368. 10.1038/nature22988.
5. Dong, Y.Y., Pike, A.C., Mackenzie, A., McClenaghan, C., Aryal, P., Dong, L., Quigley, A., Grieben, M., Goubin, S., Mukhopadhyay, S., et al. (2015). K2P channel gating mechanisms revealed by structures of TREK-2 and a complex with Prozac. Science *347*, 1256-1259. 10.1126/science.1261512.
6. Roy-Chowdhury, S., Jang, S., Abderemane-Ali, F., Naughton, F., Grabe, M., and Minor, D.L., Jr. (2025). Structure of the human K(2P)13.1 channel reveals a hydrophilic pore restriction and lipid cofactor site. Nat Struct Mol Biol. 10.1038/s41594-024-01476-3.
7. Li, B.B., Rietmeijer, R.A., and Brohawn, S.G. (2020). Structural basis for pH gating of the two-pore domain K(+)channel TASK2. Nature *586*, 457-462. 10.1038/s41586-020-2770-2.
8. Baker, N.A., Sept, D., Joseph, S., Holst, M.J., and McCammon, J.A. (2001). Electrostatics of nanosystems: application to microtubules and the ribosome. Proc Natl Acad Sci U S A *98*, 10037-10041. 10.1073/pnas.181342398.
9. Hall, P.R., Jouen-Tachoire, T., Schewe, M., Proks, P., Baukrowitz, T., Carpenter, E.P., Newstead, S., Rodstrom, K.E.J., and Tucker, S.J. (2025). Structures of TASK-1 and TASK-3 K2P channels provide insight into their gating and dysfunction in disease. Structure *33*, 115-122 e114. 10.1016/j.str.2024.11.005.
10. Laskowski, R.A., and Swindells, M.B. (2011). LigPlot+: Multiple Ligand-Protein Interaction Diagrams for Drug Discovery. Journal of Chemical Information and Modeling *51*, 2778-2786. 10.1021/ci200227u.
11. Guizouarn, H., Gabillat, N., Motais, R., and Borgese, F. (2001). Multiple transport functions of a red blood cell anion exchanger, tAE1: its role in cell volume regulation. J Physiol *535*, 497-506. 10.1111/j.1469-7793.2001.t01-1-00497.x.
12. Wimley, W.C., Creamer, T.P., and White, S.H. (1996). Solvation energies of amino acid side chains and backbone in a family of host-guest pentapeptides. Biochemistry *35*, 5109-5124. 10.1021/bi9600153.
13. Creighton, T.E. (1993). Proteins : structures and molecular properties, 2nd Edition (W.H. Freeman).
