# Supplementary material for: Structural evidence that a lipid plug controls K2P6.1(TWIK-2) function
Source: Nat Commun. 2026 Jun 16;17:7959. doi: 10.1038/s41467-026-74535-6 (PMC13448071; doi:10.1038/s41467-026-74535-6)
Supplement: Supplementary file 2 [file 41467_2026_74535_MOESM2_ESM.pdf]

Description of Additional Supplementary Files

File Name: Supplementary Movie 1

Description: Arg257 and lipid plug headgroup interaction dynamics. Molecular dynamics trajectory showing the motion of residue Arg257 (gold ball and stick) and lipid plug POPC (grey) in TWIK2 structure during 1 µs unrestrained simulation. For reference, the TWIK2:ND cryo-EM structure is shown as a transparent magenta with Arg257 shown as sticks.
