# Supplementary material for: Structural evidence that a lipid plug controls K2P6.1(TWIK-2) function
Source: Nat Commun. 2026 Jun 16;17:7959. doi: 10.1038/s41467-026-74535-6 (PMC13448071; doi:10.1038/s41467-026-74535-6)
Supplement: Supplementary file 3 [file 41467_2026_74535_MOESM4_ESM.pdf]

nature portfolio

Corresponding author(s): Daniel L. Minor, PhD

Last updated by author(s): Apr 28, 2026

Reporting Summary

Nature Portfolio wishes to improve the reproducibility of the work that we publish. This form provides structure for consistency and transparency in reporting. For further information on Nature Portfolio policies, see our Editorial Policies and the Editorial Policy Checklist.

Statistics

For all statistical analyses, confirm that the following items are present in the figure legend, table legend, main text, or Methods section.

n/a	Confirmed	
☐	☒	The exact sample size (*n*) for each experimental group/condition, given as a discrete number and unit of measurement
☐	☒	A statement on whether measurements were taken from distinct samples or whether the same sample was measured repeatedly
☐	☒	The statistical test(s) used AND whether they are one- or two-sided<br>*Only common tests should be described solely by name; describe more complex techniques in the Methods section.*
☐	☒	A description of all covariates tested
☒	☐	A description of any assumptions or corrections, such as tests of normality and adjustment for multiple comparisons
☐	☒	A full description of the statistical parameters including central tendency (e.g. means) or other basic estimates (e.g. regression coefficient) AND variation (e.g. standard deviation) or associated estimates of uncertainty (e.g. confidence intervals)
☐	☒	For null hypothesis testing, the test statistic (e.g. *F*, *t*, *r*) with confidence intervals, effect sizes, degrees of freedom and *P* value noted<br>*Give P values as exact values whenever suitable.*
☒	☐	For Bayesian analysis, information on the choice of priors and Markov chain Monte Carlo settings
☒	☐	For hierarchical and complex designs, identification of the appropriate level for tests and full reporting of outcomes
☒	☐	Estimates of effect sizes (e.g. Cohen's *d*, Pearson's *r*), indicating how they were calculated

Our web collection on statistics for biologists contains articles on many of the points above.

Software and code

Policy information about availability of computer code

Data collection

UCSF and Stanford-SLAC Cryo-EM Center (S2C2)
SerialEM, Mastronarde, D. N. Automated electron microscope tomography using robust prediction of specimen movements. J Struct Biol 152, 36-51, doi:10.1016/j.jsb.2005.07.007 (2005).

Data analysis

cryoSPARC-3.2, Punjani, A., Rubinstein, J. L., Fleet, D. J. & Brubaker, M. A. cryoSPARC: algorithms for rapid unsupervised cryo-EM structure determination. Nat Methods 14, 290-296, doi:10.1038/nmeth.4169 (2017).

RELION-3.1 Zivanov, J. et al. New tools for automated high-resolution cryo-EM structure determination in RELION-3. Elife 7, doi:10.7554/eLife.42166 (2018).

Phyre2 server, Kelley, L. A., Mezulis, S., Yates, C. M., Wass, M. N. & Sternberg, M. J. The Phyre2 web portal for protein modeling, prediction and analysis. Nat Protoc 10, 845-858, doi:10.1038/nprot.2015.053 (2015).

PHENIX, Liebschner, D. et al. Macromolecular structure determination using X-rays, neutrons and electrons: recent developments in Phenix. Acta Crystallogr D Struct Biol 75, 861-877, doi:10.1107/S2059798319011471 (2019).

COOT, Emsley, P., Lohkamp, B., Scott, W. G. & Cowtan, K. Features and development of Coot. Acta Crystallogr D Biol Crystallogr 66, 486-501, doi:10.1107/S0907444910007493 (2010).

ChimeraX, Pettersen, E. F. et al. UCSF ChimeraX: Structure visualization for researchers, educators, and developers. Protein Sci 30, 70-82,

doi:10.1002/pro.3943 (2021).

MolProbity, Williams, C. J. et al. MolProbity: More and better reference data for improved all-atom structure validation. Protein Sci 27, 293-315, doi:10.1002/pro.3330 (2018).

CHARMM-GUI Jo, S., Kim, T., Iyer, V.G. & Im, W. CHARMM-GUI: a web-based graphical user interface for CHARMM. J Comput Chem 29, 1859-65 (2008)

GROMACS 2024.2 Abraham, M.J. et al. GROMACS: High performance molecular simulations through multi-level parallelism from laptops to supercomputers. SoftwareX 1-2, 19-25 (2015)

EWALD Darden, T., York, D. & Pedersen, L. Particle Mesh Ewald - an N.Log(N) Method for Ewald Sums in Large Systems. Journal of Chemical Physics 98, 10089-10092 (1993)

Electrophysiology data were acquired using Clampex 11.2 (Molecular Devices) and analyzed with Clampfit 10.6 (Molecular Devices).

For manuscripts utilizing custom algorithms or software that are central to the research but not yet described in published literature, software must be made available to editors and reviewers. We strongly encourage code deposition in a community repository (e.g. GitHub). See the Nature Portfolio guidelines for submitting code & software for further information.

Data

Policy information about availability of data

All manuscripts must include a data availability statement. This statement should provide the following information, where applicable:

- Accession codes, unique identifiers, or web links for publicly available datasets
- A description of any restrictions on data availability
- For clinical datasets or third party data, please ensure that the statement adheres to our policy

Coordinates and maps of the following have been deposited in Protein Data Bank (PDB) and Electron Microscopy Data Bank (EMDB): 9OTA, EMD-70828, EMD-75026 (K2P6.1 (TWIK2) nanodisc) and 9OTK, EMD 70837 (K2P6.1 (TWIK2) detergent). 9OV0, EMD-70885 (K2P6.1 (TWIK2RM) plugged) and 9OV9, EMD-70894 (K2P6.1 (TWIK2RM) unplugged). 9OVD, EMD-70898 (K2P6.1 (TWIK2RM) R257A plugged) and 9OVE, EMD-70899 (K2P6.1 (TWIK2RM) R257A unplugged). Prior structures and maps for analysis include 3UKM (K2P1.1 (TWIK-1)), 7SK0 (K2P1.1 (TWIK-1) pH 7.4), 7SK1 (K2P1.1 (TWIK-1) pH 5.5), 6CQ6 (K2P2.1 (TREK-1)), 4XDK (K2P10.1(TREK 2):norfluoxetine (Nfx) complex), 9BSN (K2P13.1(THIK-1)), 6RV2 (K2P3.1(TASK-1)), 6WLV (K2P5.1(TASK-2) pH 6.5), 6WM0 (K2P5.1(TASK-2) pH 8.5), EMD-25169 (K2P1.1 (TWIK1) at pH5.5). Human K2P6.1 (TWIK2) sequence is Uniprot Q9Y257. The coordinates for the first and last frames from MD simulations have been deposited in Zenodo (https://zenodo.org/records/20435921).
Requests for material should be sent to D.L.M.

Research involving human participants, their data, or biological material

Policy information about studies with human participants or human data. See also policy information about sex, gender (identity/presentation), and sexual orientation and race, ethnicity and racism.

Reporting on sex and gender	N/A
Reporting on race, ethnicity, or other socially relevant groupings	N/A
Population characteristics	N/A
Recruitment	N/A
Ethics oversight	N/A

Note that full information on the approval of the study protocol must also be provided in the manuscript.

Field-specific reporting

Please select the one below that is the best fit for your research. If you are not sure, read the appropriate sections before making your selection.

☒ Life sciences ☐ Behavioural & social sciences ☐ Ecological, evolutionary & environmental sciences

For a reference copy of the document with all sections, see nature.com/documents/nr-reporting-summary-flat.pdf

Life sciences study design

All studies must disclose on these points even when the disclosure is negative.

Sample size	Sample sizes were determined according to field standards.

Data exclusions	For cryo-EM , data were excluded using standard classification methods in CryoSPARC to remove bad particle images and reconstructions. No data were excluded from electrophysiological analysis.
Replication	For two electrode voltage-clamp studies, the results are from at least two independent oocyte batches. n = 7-8 oocytes ('n' value is found in figure legend). All replication attempts were successful.
Randomization	Randomization is not relevant. Single particle cryo-EM is based on randomly distributed particles. Electrophysiological analysis are inherently randomized as there is no bias in selecting cells for recording.
Blinding	Blinding is not relevant to this study as no subjective allocation was involved.

Reporting for specific materials, systems and methods

We require information from authors about some types of materials, experimental systems and methods used in many studies. Here, indicate whether each material, system or method listed is relevant to your study. If you are not sure if a list item applies to your research, read the appropriate section before selecting a response.

Materials & experimental systems

n/a	Involved in the study	
x		Antibodies
	x	Eukaryotic cell lines
x		Palaeontology and archaeology
	x	Animals and other organisms
x		Clinical data
x		Dual use research of concern
x		Plants

Methods

n/a	Involved in the study	
x		ChIP-seq
x		Flow cytometry
x		MRI-based neuroimaging

Eukaryotic cell lines

Policy information about cell lines and Sex and Gender in Research

Cell line source(s)	Expi293F
Authentication	ThermoFisher
Mycoplasma contamination	negative
Commonly misidentified lines (See ICLAC register)	n/a

Animals and other research organisms

Policy information about studies involving animals; ARRIVE guidelines recommended for reporting animal research, and Sex and Gender in Research

Laboratory animals	Xenopus laevis (+9 cm, unknown age)
Wild animals	No wild animals were used in this study.
Reporting on sex	Oocytes were collected from female frogs.
Field-collected samples	No field collected samples were used in this study
Ethics oversight	Oocytes were harvested from female Xenopus laevis frogs (Nasco Cat# LM00531) and housed in the UCSF Laboratory Animal Resource Center (LARC) facilities. The use of these Xenopus oocytes was approved by IACUC (protocol approval # AN193390 - 01B) and experiments were performed in accordance with University of California guidelines and regulations.

Note that full information on the approval of the study protocol must also be provided in the manuscript.

Plants

Seed stocks	n/a
Novel plant genotypes	n/a
Authentication	n/a
