# Supplementary material for: Structural evidence that a lipid plug controls K2P6.1(TWIK-2) function
Source: Nat Commun. 2026 Jun 16;17:7959. doi: 10.1038/s41467-026-74535-6 (PMC13448071; doi:10.1038/s41467-026-74535-6)
Supplement: Supplementary file 4 [file 41467_2026_74535_MOESM5_ESM.pdf]

nature portfolio

Peer Review File

Structural evidence that a lipid plug affects K_{2P}6.1(TWIK-2) function

Corresponding Author: Professor Daniel Minor

This file contains all reviewer reports in order by version, followed by all author rebuttals in order by version.

Attachments originally included by the reviewers as part of their assessment can be found at the end of this file.

Version 0:

Reviewer comments:

Reviewer #1

(Remarks to the Author)
In this manuscript, Abhisek and colleagues report cryoEM structures of TWIK2. They observe additional density within the channel cavity, which they attribute to bound lipids. These putative lipids are proposed to occlude the pore, thereby inhibiting potassium ion transport. Structures of mutant forms appear to have a subset of particles that do not contain the additional density in the channel cavity. The authors also present electrophysiological data.

The central focus of this manuscript is the additional density observed within the channel cavity of TWIK2, which the authors interpret as two lipid molecules occluding the pore. However, I have several major concerns regarding this interpretation. Except for the structure obtained in nanodiscs, the overall quality and resolution of the cryoEM maps are relatively low. Whilst I agree that there is extra density in the central cavity, I am not convinced that it corresponds to two bound lipids. At a contour level where side chains and lipid-like density within the transmembrane domain (near F201 and L202) are clearly resolved, the density in the central cavity is discontinuous and largely absent for most of the putative lipid molecules. Although the density appears more continuous at lower contour levels. Moreover, according to Table 1, the reported B-factors for the ligands are lower than those of the protein in all cases. This is unexpected (perhaps mistakenly swapped). If correct, it would imply that the ligand density should be even more prominent at higher contour levels, which is not observed. In addition, the authors should perform refinement in C1 symmetry to demonstrate that the density observed in the central cavity is consistent with that obtained using C2 symmetry. This comparison would provide justification for the imposition of C2 symmetry and help rule out potential artifacts introduced by symmetry enforcement.

Another point that needs to be addressed is the molecular interactions of the lipid within the cavity. In most cases, no headgroup is modeled for these lipids, consistent with the absence of corresponding density. This is not surprising, as density for either the headgroup or the acyl chains is often only partially resolved for bound lipids in cryoEM structure. In the R257A plugged structure, a choline headgroup from phosphatidylcholine is modeled. In this model, the two positively charged headgroups are oriented directly toward each other, which is electrostatically unfavorable. More generally, while the modeled acyl chains appear to interact with surrounding hydrophobic residues as expected, there are no clear molecular contacts involving the glycerol backbone (and headgroup) of the phospholipid. This absence of interaction raises additional questions about the modeled lipids and interpretation.

The authors attempt to probe the proposed central cavity lipid-binding site using point mutations. Electrophysiology studies indicate enhanced channel activity for some mutants. However, the functional and structural data is not entirely consistent with the proposed lipid plug model. For example, R257A should not perturb lipid binding but activity is enhanced. Moreover, cryoEM data for RM-R257A shows a higher percentage of particles for plugged vs unplugged. Compare this to RM that has near equal number of particles for plugged and unplugged. If R257A were disrupting lipid binding, one would expect a reduction in the plugged particle population (not an increase). This discrepancy undermines the central hypothesis that lipids bind in the central cavity.

Building on the points above, I also have several concerns regarding the structural models. The density for portions of the "ear helix" is weak or absent in the structures, yet these regions are still modeled. Similarly, potassium ions are placed in sites where the density is either weak or entirely missing. For example, in the nanodisc structure, the S0 site lacks any

discernible density, yet a potassium ion is modeled there. More generally, there are multiple regions of the protein that have been modeled despite the absence of density. These regions should be excluded from the final models to avoid overinterpretation. Additionally, in Figure 1e, Tyr111 is shown adopting two distinct conformations in the nanodisc structure. However, based on the map provided, only one conformation is supported by the density.

As noted above, these structures, including those from others in this family, show additional density in the central cavity. However, what is severely lacking from this paper is the identity of the lipid(s). While cryoEM maps can suggest the presence of bound lipid-like density, they rarely provide sufficient resolution to determine lipid identity with confidence. In this case, the assignment remains speculative. Additional biochemical or mass spectrometry-based experiments are necessary to establish whether specific lipids co-purify with the channel and to confirm their identities. In the absence of such supporting evidence, the current interpretation remains too circumstantial to support the central claim of a lipid plug.

Some other comments:

Non-standard or imprecise terms are used throughout. For examples, the acyl chains of the lipid are described as "legs" or "bifurcated". A more accurate description would refer to the sn-1 and sn-2 positions of the acyl chains. The term "bidentate" is also misused, which refers to ligand coordination with metals.

The lipid is often shown in sphere representation. In other cases, cryoEM density is shown for the lipid but, in some cases, it appears that a low contour map has been selectively carved around the modeled lipid, which may be misleading.

The manuscript in some parts is rough, and difficult to read.

Reviewer #2

(Remarks to the Author)
Please see attached pdb which contains the comments for authors, as they include figures.

[Editorial Note: See attachment at end of file]

Reviewer #3

(Remarks to the Author)
I co-reviewed this manuscript with one of the reviewers who provided the listed reports. This is part of the Nature Communications initiative to facilitate training in peer review and to provide appropriate recognition for Early Career Researchers who co-review manuscripts.

Reviewer #4

(Remarks to the Author)
In this study Mondal and colleagues report a series of cryo-EM structures of the TWIK-2 (K2P6.1) channel, including wild-type and trafficking mutant channels in nano-discs and detergent, and an additional novel R257A mutant channel in detergent. These structures confirm some characteristic features of TWIK-2 first shown recently (and at higher overall resolution) in another study, as referenced [51], but also suggest a novel 'lipid plug' interaction, which is proposed as 'a mechanism to render the TWIK channels inactive'.

As the authors discuss, lipid-gating of (other) K2P channels has been proposed but not conclusively demonstrated, in part due to the challenging nature of testing these hypotheses. These new structures, and the re-evaluation of older TWIK-1 structures which suggest similar occupancy of both upper and lower fenestrations, raises an interesting potential new mechanism of channel regulation. However, ultimately, I feel that this study doesn't quite provide the killer experimental demonstration of this mechanism, unfortunately. I feel that this also means that the manuscript title is too assertive.

I suggest that the new data and potential mechanism are sufficiently interesting to warrant publication as a working model (with some concerns, as listed below) but this appears to be an opportunity to fully dissect what would be a novel K+ channel gating behavior. I hope the authors are allowed time to test proposed findings more thoroughly.

Major Concerns:
• The density attributed to the lipid plug is novel in showing occupancy of the fenestrations consistent with a two-tailed native cell membrane phospholipid (and not a detergent molecule) which can block the permeation pathway. The identity of this lipid is not well determined, however. Lines 221-222 suggest contacts with the C-helix are 'consistent with phosphatidylethanolamine head groups" but it is not clear how this is substantiated. Figures 5A,B, S18A, do not convincingly show this.
o Are there mutations in the C-helix which would be predicted to disrupt headgroup interactions? What are the effects of such mutations?
• The mutagenesis experiments can be interpreted as consistent with the lipid-plug model but alone fail to convince that this exists as a functional mechanism. Previously, mutation of the equivalent of M135 in TWIK-1 (L146) has previously been implicated in altered hydrophobic gating. The M>D mutation which increased TWIK-2 currents could also impart this effect

via reduced de-wetting. If a greater range of M135 substitutions fails to follow the relationship between hydrophobicity and TEVC currents shown for TWIK-1 in ref 49, this could disfavor a hydrophobic gating mechanism and support an alternative, such as the lipid plug mechanism.
• Given the resolution of the plug density, the precise location of the atoms of any lipid appears difficult to discern. Thus, the suggestion that "The Arg257 sidechain, a residue conserved in vertebrate TWIK2 (Fig. S5), directly contacts the lipid glycerol backbone" is not well established. It's also not made clear why the R257A substitution would lead to the lower lipid binding pose.
• Strengthening this proposed model would require significant additional work, which could include MD simulations, mass spec analysis of bound lipids, or determining reconstituted channel activity in liposomes of defined composition (including altered PE levels) via the ACMA assay. Clearly, each are specialist methods, but data from any could elevate confidence in the proposed model.
• The lantibiotic, duramycin (Sigma #D3168) is reported to selectively bind to PE (PMID: 21573677). Is it possible to record TWIK2 currents in giant patches and apply this?
• The lipid plug conferring subcellular regulation is a neat idea, but what are the relative lipid compositions in the lysosome and plasma membrane? Are these compositions consistent with the RM mutation (and consequently increased plasma membrane trafficking) being likely to reduce PE binding as suggested from observation of unplugged RM mutant channels? Or binding by another possible candidate lipid?

Other comments:
• The flipped-out conformation of Y111 is consistent with the recent structures from the Long group. The inactivation shown in S14 in these mutations Is interesting. Is this stabilized by elevated K+ (it seems the authors should have this data as high K+ recordings were made as shown in S14d)?
• As noted, the orientation of this residue has been associated with pH gating in TWIK1 and in TASK channels. Does the Y>H mutation impart pH gating to TWIK2?
• The step-traces for M135D, R257A, and R257E mutations in Fig S14 appear to show that these mutations affect the voltage-activation of the channels, in that there is a significant increase in outwards currents at the -80 mV holding potential from these mutations compared with TWIK2RM. Was this consistent across recordings and can averaged data from these step protocols be included in this figure? This would be an interesting biophysical consequence, which is not discussed.
• The use of 'leg' for the lipid acyl chains was a little confusing, is 'tail' not more standard?
• The x axis on S14b,d is presumably off by a value of 1. Where log10 [K+, mM] = 3 should be 2, consistent with a reversal potential of ~0mV. Can the authors confirm?
• Suggest to remove 'a' in: Structures of a previously characterized K2P6.1 (TWIK2), a mutant that increases the plasma membrane localization and activity, yield plugged and unplugged forms that highlight the importance of lipid removal for channel function.
• Suggest to add 'at' in: under conditions associated with inactivation of the channel C-type SF gate highlight the importance of structural changes [AT] this site for K2P function.
• Figure 3 legend includes 'd' twice, second one should be 'e'
• Figure 5 legend includes 'd' twice, first one should be 'c'

Version 1:

Reviewer comments:

Reviewer #1

(Remarks to the Author)
The revised manuscript appropriately moderates several of its original claims in response to reviewer critiques.

Previous structural studies have reported density within the TWIK channel cavity, and the present study similarly identifies comparable density that the authors interpret by modeling two lipid molecules. While this density should not be ignored, as noted by myself and Reviewer 3 in the initial review, additional experimental validation is required to substantiate both lipid occupancy and identity. At present, I remain unconvinced that cryo-EM data alone provide sufficient evidence to confidently assign these features as bound lipids, or indeed to definitively distinguish them from other possible molecular occupants. Considerable interpretive weight is placed on the density maps, and alternative explanations cannot be excluded. While I recognize the technical challenges associated with obtaining orthogonal validation, the current interpretation risks overstating what can be concluded from structural data alone. The Discussion should more explicitly acknowledge this limitation and emphasize that the presence, identity, and functional relevance of lipids within the channel cavity remain open questions in the field.

The molecular dynamics (MD) simulation section would benefit from further clarification. As currently described, simulations were initiated using structures containing the two modeled lipids within the cavity, leading to the conclusion that the simulations show stable lipid plug positions. This outcome is somewhat expected given the initial conditions and raises additional questions regarding how such lipids would exit the pore. Simulations initiated from lipid-free "unplugged" structures could provide complementary insight into whether lipids can spontaneously access the cavity. While such analyses may reasonably fall beyond the scope of the current manuscript, acknowledging this limitation would strengthen the interpretation.

The authors also report reduced biochemical stability for several channel mutants. However, decreased stability may complicate interpretation of the functional and structural results. In their response, the authors indicate that altered

biochemical stability precluded additional experiments on these variants. This limitation should be more explicitly discussed, as instability of the mutant channels may introduce ambiguity in attributing observed effects specifically to lipid interactions.

Reviewer #2

(Remarks to the Author)
We appreciate the authors' efforts to further support their structural interpretation of lipid molecules occluding the pore and thank them for clarifying the density in their response to the reviewers. As the authors note, limitations in resolution and conformational heterogeneity are common for lipids in cryo-EM maps, with clear differences in density, for example, between their TWIK2 WT nanodisc and detergent maps. Although the modeled orientation of the lipid(s) is therefore uncertain, and we would still urge caution in model building, it is true that this does not alter the overall interpretation of the authors' proposed pore-plugging model. We also agree that the observation that the mutations destabilize the protein, potentially due to loss of the lipid ligand(s), is qualitative but a reasonable and helpful addition to the manuscript.

The addition of all-atom MD simulations further strengthens the study, demonstrating that a modeled phospholipid can remain stably positioned within the cavity and that Arg257 contributes to its stabilization. However, in our original critique, we stated that mechanistic support would require, "MD simulations to assess lipid ingress/egress through fenestrations and correlate with plug stability and M4/C-helix motion and site-directed mutagenesis of M4/C-helix residues flanking the plug site to smaller (e.g., A) or bulkier (e.g., W) side chains and test for effects on plug stability and gating via cryo-EM or functional assays." While we appreciate the effort to perform MD simulations in support of plug stability, the other requested analyses were not addressed. It would be helpful if the authors could clarify why these were not pursued, as they are central to developing a mechanistic interpretation of lipid plug gating.

Overall, the current manuscript establishes a correlation between lipid density, residue interactions, and functional effects, but does not yet define the molecular pathway governing plug insertion, removal, or exchange. Thus, while the structural observations are intriguing and supported by MD simulations and structure–function analysis, the proposed gating model requires additional mechanistic support. In principle, such evidence would come from further computational sampling of lipid transit pathways and/or targeted mutagenesis of the proposed ingress/egress interface, as suggested previously.

If the authors are unable to perform these additional experiments, then the manuscript will need to be reorganized to deemphasize the lipid plug gating mechanism, as this remains a working model. Although the authors have already taken steps in this direction, the current phrasing still reads stronger than the available evidence supports.

Reviewer #3

(Remarks to the Author)
I co-reviewed this manuscript with one of the reviewers who provided the listed reports. This is part of the Nature Communications initiative to facilitate training in peer review and to provide appropriate recognition for Early Career Researchers who co-review manuscripts.

Reviewer #4

(Remarks to the Author)
The authors have adequately addressed my comments and with the additional data from the MD simulations, in my opinion, this work is sufficiently interesting to warrant publication as a working model. Notably, however, I am not a cryo-EM expert and defer to the apparent greater expertise from the other reviewers on these details.

Reviewer #5

(Remarks to the Author)
Please note that the reviewer's expertise is in MD simulations. Thus, this review will focus on the rigour within MD simulation methodology and the interpretation of the results. The MD simulations section of this manuscript seems to have been added in a very rushed manner, with one major flaw. This needs serious consideration before moving forward to acceptance.

Major point

1. Figure 6g. It is stated that "The R257A change results in higher mobility of the lipid in the lipid head moiety (Fig. 6g)" under "Arg257 makes dynamic interactions with the lipid plug". The data, however, IS THE OTHER WAY ROUND. The RMSD of the head is LOWERED, suggesting a more stable binding. Unless the author has mislabelled the data, the conclusion does not hold together. The author also didn't state how the RMSD is calculated. The caption of the figure stated "RMSD plot of the lipid head relative to the starting position defined by the TWIK2:ND structure". Starting position of WHAT? The CA protein, the lipid headgroup - every atom? heavy atoms?. These matter massively in the value of the RMSD.

2. The authors stated that the MD simulations were conducted for 1 us, in 3 independent conditions. WT, R257A and R257K. If I understood that the figure is labelled wrongly, the data is indeed convincing on the importance of R257 as a key contacting residue. However, the conclusion is drawn from just 1 repeat of the MD simulations. I would request that the author perform at least 3 replicas for each condition to ensure that the POPC is indeed stable in the binding pocket.

3. If the label were right, then please explain why having an alanine in the binding site is more stable than the arginine or lysine.

Minor points

1. The figure legend shows a running average and a shaded average with no caption to explain what each of them means. Is it a running average? Average from 3 repeats? This needs contextualisation.

2. The H-bonding plot could be collapsed as a histogram, with the x-axis being the number of H-bonds, and the y-axis being the fractions of frames with H-bond form. With this, together with 3 repeats, you can statistically compare the tendency of H-bond formation between WT and the mutant.

3. Caption Figure 6e, "Exemplar plot of Arg257 bridging involving coordination of phosphates from two lipids," makes no sense when the graph is plotting the number of bridging interactions. You mean the number of salt bridges between the phosphate and the guanidinium group? Then why two lipids? This is very unclear.

4. Again, the caption "h, Comparison of R257A lipid from simulation with the positions observed in TWIK2 and R257A.and h, TWIK2 R257K. Inset shows atom group." Why are there two h. You mean h and i? And you meant the same thing as g? And if you are going to say the same thing as g - why bother having 2 separate plots? You can have a single plot with 3 lines. If you have 3 repeats each, you can collapse all the data into a single histogram.

5. Figure 6h is just lipids floating in vacuo with no context from the protein structure. Why? It is bizarre to communicate those points without the side chain around it to bring the context together.

Reviewer #6

(Remarks to the Author)
The manuscript is largely convincing and uncovers an important regulation mechanism of TWIK channels. We mostly comment on MD simulations.

The lipid plug hypothesis is plausible. However, one cannot rule out that the channel might not conduct due to dehydration of the cavity or due to a reduction of an electrostatic energy barrier, without the need for the lipid to penetrate. In that case, lipid entry could be a consequence rather than the cause. For example, the R257A mutation removes a positive electrostatic barrier along the permeation pathway, which could explain the larger currents in these mutants without invoking lipid blockage, since potassium ions are no longer repelled by the arginine. Ideally, to bring this point home, one could try simulations of the channel in the absence of the lipid, and report metrics such as water and ion density in the cavity in both cases. Given that this is not critical I won't push for these simulations in the current manuscript, but these points should be mentioned in the discussion.

RMSD can be a degenerate metric. For the purpose of showing binding poses, it is better to perform clustering using RMSD as the metric and show the most populated cluster poses in the simulation, which in principle should correspond to the poses seen in the density, since both should, in principle, represent an energy minimum. Right now, the comparison shown in Fig6h seems arbitrary in terms of the lipid pose picked up from MD.

In the same spirit, we would recommend to calculate the average lipid density from the simulation and compare it with the cryo-EM density. This would be a qualitative way to check that the lipid seen in the structure is actually plausible under simulation conditions, and vice versa, as shown by Aponte-Santamaria et al., PNAS 2012.

To better assess the dynamic nature of the bound lipid, RMSF analysis would be better suited (or could complement) the segment-based RMSD - the fact that RMSD is large with respect to the starting position does not mean that the lipid shows a dynamical behaviour throughout the simulation.

Residue M135 is important for the current response of this channel and also seems to interact with the lipid, but no structural analysis is carried out of how this residue interacts with the lipid. Some more discussion could be provided on this.

Version 2:

Reviewer comments:

Reviewer #1

(Remarks to the Author)
The authors have addressed my concerns and recommend for publication.

Reviewer #2

(Remarks to the Author)
I have no additional comments. We appreciate the authors' efforts to address our concerns and revise the wording.

Reviewer #5

(Remarks to the Author)
We thank the author for the revision of the manuscript. I am happy with the EPhys and the structural work which is clearly the expertise of the authors. However, there are still some error on MD simulation to my concern as follow.

1. In all of your RMSD calculation, it is said that it is fitted to the initial structure. If that so, t = 0 should have 0 RMSD. Why is it not beginning with 0? At the end of your simulation (1000 ns), the RMSD decreases - which is rather odd unless it goes back to the fitted structure. What you plot are more likely corresponded to the RMSD fitted to the end state?

2. This figure legend

d, Exemplar plot Arg257-phosphate hydrogen bonds made by
one sidechain.

e, Exemplar plot of Arg257 bridging involving coordination of phosphates from
two lipids by forming bidentate hydrogen bonds.

This makes no sense. You are trying to say that R257 forms less hydrogen bond with 2 lipids comparing to 1 lipid? or e only counts the case when the R257 forms two hydrogen bond simultaneously with 1 H-bond to two lipids? Can you please rephrase your figure legend, or show clearly in your figure what are you actually calculating?

3. Again - the mutation to A makes RMSD value lowered (orange). This suggests stabilisation of the lipid, not that the lipid is more dynamics. If this is calculated with respect to the starting structure, then clearly R to A mutation stabilised the lipid. Is this another mislabelling? If your observation is indeed true, you need to change your metric of evaluating headgroup dynamics within the binding pocket.

Reviewer #6

(Remarks to the Author)
All my points were sufficiently addressed

Version 3:

Reviewer comments:

Reviewer #5

(Remarks to the Author)
The authors have addressed my concerns and recommend for publication.

Most issues in the past came from the figure legend and I appreciate the reviewer for the corrections.

Open Access This Peer Review File is licensed under a Creative Commons Attribution 4.0 International License, which permits use, sharing, adaptation, distribution and reproduction in any medium or format, as long as you give appropriate credit to the original author(s) and the source, provide a link to the Creative Commons license, and indicate if changes were made.
In cases where reviewers are anonymous, credit should be given to 'Anonymous Referee' and the source.
The images or other third party material in this Peer Review File are included in the article's Creative Commons license, unless indicated otherwise in a credit line to the material. If material is not included in the article's Creative Commons license and your intended use is not permitted by statutory regulation or exceeds the permitted use, you will need to obtain permission directly from the copyright holder.
To view a copy of this license, visit https://creativecommons.org/licenses/by/4.0/

We thank the reviewers for their constructive criticisms of our work. We outline below our responses to their concerns. Our revised manuscript contains changes, new functional data, and new molecular dynamics simulations that address the concerns and provide further support for our proposed mechanism of TWIK2-lipid interactions. Our responses are in blue text.

Reviewer #1

In this manuscript, Abhisek and colleagues report cryoEM structures of TWIK2. They observe additional density within the channel cavity, which they attribute to bound lipids. These putative lipids are proposed to occlude the pore, thereby inhibiting potassium ion transport. Structures of mutant forms appear to have a subset of particles that do not contain the additional density in the channel cavity. The authors also present electrophysiological data.

The central focus of this manuscript is the additional density observed within the channel cavity of TWIK2, which the authors interpret as two lipid molecules occluding the pore. However, I have several major concerns regarding this interpretation. Except for the structure obtained in nanodiscs, the overall quality and resolution of the cryoEM maps are relatively low. Whilst I agree that there is extra density in the central cavity, I am not convinced that it corresponds to two bound lipids. At a contour level where side chains and lipid-like density within the transmembrane domain (near F201 and L202) are clearly resolved, the density in the central cavity is discontinuous and largely absent for most of the putative lipid molecules.

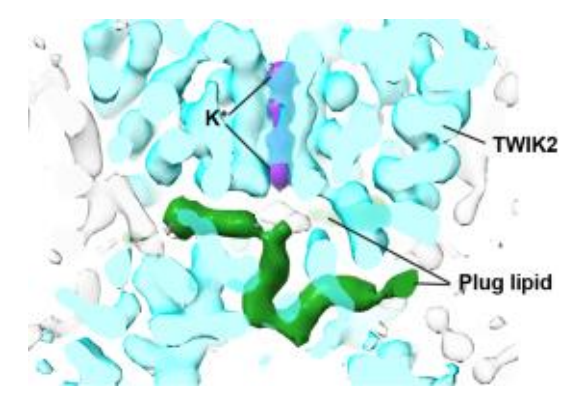

Fig. R1 TWIK2:ND map. Protein (blue) and lipid plug (green) are indicated.

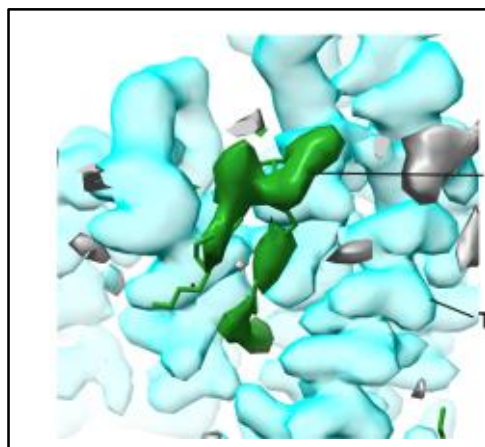

Fig. R2 TREK-1 POPA site (EMD-27387) Protein (blue) and POPA (green) are indicated.

The density that we see in the central cavity is impossible to ignore (Fig. R1). The shape, even when viewed at contours that show breaks, is consistent with a pair of two-tailed lipids. Because one cannot rationalize how such a state can represent a functional channel, given that the central ion pathway is blocked, we pursued extensive structural studies to understand if there are conditions in which the channel is not plugged. We agree that at high contour thresholds, the cavity densities are weaker than that surrounding protein density and show breaks. This behavior often seen for lipids in cryo-EM studies of membrane proteins. For instance, examination of the deposited map (EMD-27387, 2.8Å) for as study of lipid interactions with the K2P channel TREK-1 (PDB 8DE8) (Schmidpeter *et al.*, *Nat Commun*, 2023) (Fig. R2) shows discontinuous density adjacent to well-resolved protein side chains that are assigned to phospholipid (in this case POPA). Further, Turney *et al.* (*Nat Struct Mol Biol*, 2021) reported that the acyl-chain density below the TWIK-1 filter was visible only at lower contour levels, highlighting that the protein and lipid features differ in the maps. These examples illustrate that lipid tails often do not have the same density quality with surrounding protein elements in cryo-EM maps and that what we observe is not atypical. It is likely that the differences in densities reflect different mobilities for the protein and lipids, or heterogeneity in the lipid type. Hence, we do not think our interpretation is outside of field standards, especially as we have extensive structural, functional, and now, computational studies to support our ligand assignment.

To address this issue further, we have performed 1 µs simulations of TWIK2 and two mutants, R257A and R257K. Overall, the TWIK2 simulations show that the lipid plug conformation we model is stable (Figs. 6c, S20a-f) and reveal that Arg257 makes a dynamic set of interactions with the phosphoglycerol backbone (Figs. 6d-f, Movie S1), consistent with the poorly resolved density for this residue. Importantly, we find that the stability of the lipid position is linked to the identity of the sidechain at residue 257. The R257A mutation results in movement of the lipid position towards the intracellular side of the channel that are consistent with our structural observations

(Figs. 6g & h). Further, the simulations show that R257K behaves much like WT and highlight the role of this residue position in coordinating interactions with the plug lipids. This new mutant also shows functional properties that match the WT channel when measured on the $TWIK2_{RM}$ background that permits plasma membrane expression (Fig. 4e). Together, these data corroborate our conclusion that Arg257 is a key position for the function of the channel.

We also thank the Reviewer for pointing out the clear lipid density near F201 and L202. Our revised model of the TWIK2:nanodisc structure now includes this ligand. Its presence is noted on p. 5 of the 'results'.

Although the density appears more continuous at lower contour levels. Moreover, according to Table 1, the reported B-factors for the ligands are lower than those of the protein in all cases. This is unexpected (perhaps mistakenly swapped). If correct, it would imply that the ligand density should be even more prominent at higher contour levels, which is not observed.

This difference in B-factors is likely the consequence of averaging the varied B-factors to a single number and the fact that the ligand values represent both the selectivity filter ions and the lipids. There are many more components from the channel and these have a much wider range of B-factors than the ligands. These average numbers cannot convey the details for select features. This is why we include the B-factor map for all structures (ex. Fig.S2d). This representation allows the reader to make a more accurate comparison of different structural features.

In addition, the authors should perform refinement in C1 symmetry to demonstrate that the density observed in the central cavity is consistent with that obtained using C2 symmetry. This comparison would provide justification for the imposition of C2 symmetry and help rule out potential artifacts introduced by symmetry enforcement.

Our initial structure solution done in C1 for all maps reported in the manuscript as indicated in Figures S1e, S9e. S11e, and S16e. Maps that resulted in plugged structures all showed the pair of U-shaped densities in the central cavity in C1. An example from the TWIK2:ND structure is shown here (Fig. R3). Imposition of C2 symmetry did not fundamentally change this feature but was important for improving overall map quality. To make it clear that C1 was used we now list the 'Non-uniform refinement (C1)' step in Figs Figures S1e, S9e. S11e, and S16e and have added the following to the methods (p.35-36) (new text in italics):

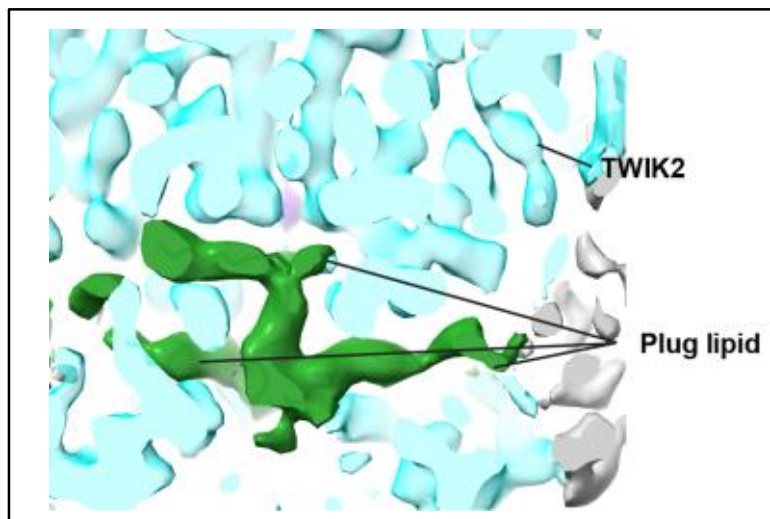

Fig. R3 TWIK2:ND map after initial C1 processing. Protein (blue) and Lipid plug (green) are indicated.

> Several rounds of 2D classification were performed and further cleaning was done by categorizing particles by 3D *ab initio* reconstruction *using C1 symmetry. Non-uniform refinement was performed on the particle set belonging from best ab-initio map using C1 symmetry.* Finally, non-uniform and local refinement were performed *using C2 symmetry* to improve resolution and map quality. Datasets corresponding to $TWIK2_{RM}$ and $TWIK2_{RM}$R257A yielded plugged conformations after non-uniform refinement.

Another point that needs to be addressed is the molecular interactions of the lipid within the cavity. In most cases, no headgroup is modeled for these lipids, consistent with the absence of corresponding density. This is not surprising, as density for either the headgroup or the acyl chains is often only partially resolved for bound lipids in cryoEM structure. In the R257A plugged

structure, a choline headgroup from phosphatidylcholine is modeled. In this model, the two positively charged headgroups are oriented directly toward each other, which is electrostatically unfavorable. More generally, while the modeled acyl chains appear to interact with surrounding hydrophobic residues as expected, there are no clear molecular contacts involving the glycerol backbone (and headgroup) of the phospholipid. This absence of interaction raises additional questions about the modeled lipids and interpretation.

We have removed the headgroup from the R257A structure.

Given the constraints imposed by the overall structure, Arg257 faces the interior cavity where we see the lipid density. We have addressed lipid interactions by using molecular dynamics simulations to investigate the behavior of a pair of POPC lipids in the central cavity. These show that Arg257 can make multiple interactions with the phosphoglycerol head group (Figs. 6a, b, & d-f). This varied coordination indicates that Arg257 is dynamic, a behavior that is consistent with its poorly resolved density. Importantly, simulations of the R257A mutation result in a drop of the lipid towards the intracellular side of the channel (Figs. 6 g-h). This behavior is consistent with our experimental observations. These new simulations provide further support of our original interpretation.

To clarify the issue about the Arg257 density, we have rewritten the description as follows (p. 6) (new text in italics).

> The lipid lower *tail* site is formed by M2 and M4 and is lined by Leu136, Leu253, Arg257, Ser260, Thr266, and Leu270 (Figs. 2b-d, S8a). *Although it is not well defined by the density, the overall structure of the channel places Arg257, a residue conserved in vertebrate TWIK2 (Fig. S5), at the level of the lipid glycerol backbone (Figs. 2c, S8a).* Except for Arg257, the lipid plug contacting residues are conserved between TWIK1 and TWIK2 (Fig. S8b-c).

Because of the observed interactions between Arg257 and the lipid, and destabilization of the lipid position with the R257A, we also simulated the R257K mutant. This mutant should still be able to coordinate the lipid. Indeed, our simulations show that this mutation yields stable, but dynamic, contacts to the lipid, and maintains a position similar to the native Arg257. Further, experimental measurement of the R257K mutant yields channels that are not different than wild-type (Fig. 4e), corroborating the idea that the Arg257 site is a key player in channel function.

The authors attempt to probe the proposed central cavity lipid-binding site using point mutations. Electrophysiology studies indicate enhanced channel activity for some mutants. However, the functional and structural data is not entirely consistent with the proposed lipid plug model. For example, R257A should not perturb lipid binding but activity is enhanced. Moreover, cryoEM data for RM-R257A shows a higher percentage of particles for plugged vs unplugged. Compare this to RM that has near equal number of particles for plugged and unplugged. If R257A were disrupting lipid binding, one would expect a reduction in the plugged particle population (not an increase). This discrepancy undermines the central hypothesis that lipids bind in the central cavity.

The reviewer is incorrect in stating that R257A should not affect lipid behavior. This residue is positioned to interact with the lipid head groups. This property is observed clearly in our new MD simulations comparing WT and R257A channels (Fig. 6). We see a drop in the lipid position in the R257A simulations ~3 Å over the course of a 1 µs unrestrained trajectory that mirrors the experimentally observed changes in the structure (Figs. 6g & h). This result supports the interpretation that Arg257 contributes to anchoring the lipid within the vestibule and is consistent with the structural changes we observe in the $TWIK2_{RM}$-R257A cryo-EM dataset.

The reviewer is correct regarding the expectations in the proportions of 'plugged' vs. 'unplugged' with the R257A mutant. Unfortunately, this assumption does not match the experimental reality we observed. We found that channels bearing the RM mutation that reveal the 'plugged' and 'unplugged' forms are more poorly behaved than the WT channel and unlike the WT channel partially precipitate during purification. Adding the R257A mutation onto the RM background exacerbates this problem. These observed differences, found using identical purification conditions, suggest that the poor biochemical behavior reflects decreased stability resulting from the loss of plug lipids. We now note this observation in the text (p. 8) as follows (new text in italics).

> (p. 8) The structural analysis of the trafficking mutant $TWIK2_{RM}$ shows that the channel can exist in two states, one having two lipid plug molecules (TWIK2:ND, TWIK2:Det, and $TWIK2_{RM}$:Det plugged) and one in which access to the central cavity is unhindered ($TWIK2_{RM}$:Det unplugged). *Consistent with this change in ligand occupancy, we observed that $TWIK2_{RM}$ was less stable during purification.*
>
> (p. 9) To investigate the structural consequences of perturbing *lipid plug-channel interactions*, we determined the structure of $TWIK2_{RM}$ R257A, a mutant that increases $TWIK2_{RM}$ basal currents (*Figs. 4d-e and S16*). *Notably, the introduction of this mutation affected the stability of the channel during purification beyond what was seen with $TWIK2_{RM}$.*

As we have no way of assessing how the poor biochemical behavior skews the population that we can observe on the grids, the most conservative approach is not to draw conclusions from the percentages of 'plugged' vs. 'unplugged' channels. This is why we did not make any such claims in the original manuscript. Because of this physical property of the samples, contrary to the reviewer's claim, the proportions of the two forms that we observe on the grids do not provide evidence that would undermine our central hypothesis.

Building on the points above, I also have several concerns regarding the structural models. The density for portions of the "ear helix" is weak or absent in the structures, yet these regions are still modeled. Similarly, potassium ions are placed in sites where the density is either weak or entirely missing. For example, in the nanodisc structure, the S0 site lacks any discernible density, yet a potassium ion is modeled there. More generally, there are multiple regions of the protein that have been modeled despite the absence of density. These regions should be excluded from the final models to avoid overinterpretation. Additionally, in Figure 1e, Tyr111 is shown adopting two distinct conformations in the nanodisc structure. However, based on the map provided, only one conformation is supported by the density.

The density corresponding to the ear helix is well resolved in our datasets (e.g., Figs. S2b, S10a, S12a, S13a, and S17a). We acknowledge that this region exhibits reduced resolvability in the $TWIK\text{-}2_{RM}$ mutant maps and that its loop is not well resolved. To avoid overinterpretation, we have removed the poorly resolved portions of the loop leading to the ear helix.

Regarding the S0 site, we revisited the maps and agree that interpreting this site as occupied is ambiguous. Hence, in response to this critique, we removed this ion from the models in which it was included and have corrected the corresponding figures accordingly.

The dual conformation of Tyr111 was modeled based on a locally refined map generated to improve the density quality in that region and is shown in Fig. 1e. This refinement revealed clear evidence for the alternate conformation (TWIK2:ND). The alternative conformation is less apparent in the consensus map. We have now generated and deposited a composite map that incorporates this focused refinement results (EMD-75026). This will allow readers to evaluate the density and modeling decisions. It is worth mentioning that local density cannot be reliably judged

using a single threshold level for all residues in any cryo-EM structure, since the flexibility of residues varies (Indeed, there was a recent discussion on the CCPEM thread by Oliver Clark regarding this issue ~19 Aug 25). The composite map addresses this point.

To clarify how we observed this conformation, we have added the following (p.6) new text in italics.

> Inspection of the TWIK2:ND density *using focused refinement* showed evidence for a second Tyr111 conformation corresponding to the 'down' state (Fig. 1d-e) in which Tyr111 interacts with a pocket formed by P1 helix residues Phe98, Phe99, and Thr102 and SF1 residue Tyr109 (Fig. 1d).

As noted above, these structures, including those from others in this family, show additional density in the central cavity. However, what is severely lacking from this paper is the identity of the lipid(s). While cryoEM maps can suggest the presence of bound lipid-like density, they rarely provide sufficient resolution to determine lipid identity with confidence. In this case, the assignment remains speculative. Additional biochemical or mass spectrometry-based experiments are necessary to establish whether specific lipids co-purify with the channel and to confirm their identities. In the absence of such supporting evidence, the current interpretation remains too circumstantial to support the central claim of a lipid plug.

Given the difficulty in assigning bound lipids by mass spec and biochemical changes in the stabilities of the mutant channels, we have not pursued this direction. The following data all support our interpretation: 1) WT channels show the plug, 2) channels bearing mutations that send the channel to the plasma membrane and yield active channels show two forms including the 'unplugged' form that is not seen in WT samples, and 3) perturbation of a key lipid coordinating residue (R257A) yields a change in plug position. These experimental observations are corroborated by the new MD studies. While we cannot definitively assign an identity to the lipid, our data provide a new working model for understanding these channels.

Some other comments:

Non-standard or imprecise terms are used throughout. For examples, the acyl chains of the lipid are described as "legs" or "bifurcated". A more accurate description would refer to the sn-1 and sn-2 positions of the acyl chains. The term "bidentate" is also misused, which refers to ligand coordination with metals.

Oxford English Dictionary defines 'bifurcate' as 'Divide into two forks, branches, or peaks'. This word accurately describes the density, which is divided into two connected parts, and was not changed.

'Bidentate' has been replaced with 'two-tailed' to convey the fact that each lipid tail has a binding site.

Reviewer #3 also did not like the use of 'leg' and suggested the more common term 'tail', which we have now implemented.

The lipid is often shown in sphere representation. In other cases, cryoEM density is shown for the lipid but, in some cases, it appears that a low contour map has been selectively carved around the modeled lipid, which may be misleading.

All displayed densities are derived directly from the deposited cryo-EM maps at their stated thresholds without any selective manual carving or masking around the modeled lipid.

In several figures, the lipid was shown in sphere representation to provide a simplified view for readers who may be less familiar with channel vestibule geometry. In others, we used cryo-EM density rendering (*PyMOL* or *ChimeraX*) to illustrate the continuity and fit of the lipid-like density at the corresponding contour levels.

The manuscript in some parts is rough, and difficult to read.

Without knowing specific points where the reviewer found manuscript difficult to read, we could not address this critique.

Reviewers #2 & 3 (co-reviewed)

In this study, the authors report a gating mechanism for the two-pore domain potassium (K2P) channel family member TWIK-2 (K2P6.1) involving lipid-mediated pore block. Using high-resolution cryo-EM structures, the authors propose that this lipid plug serves as a gating mechanism that functionally silences TWIK channels. The study addresses a longstanding question in the K2P field regarding the role of lipid densities seen in channel fenestrations. Based on the cryo-EM structures of TWIK-2 presented in this manuscript, comparisons with previously published TWIK-1 cryo-EM structures, and structure-function guided mutagenesis near putative lipid-binding sites, the authors propose that two acyl chain binding sites observed in TWIK-2 - one conserved among K2P channels ("upper leg") and another unique to TWIK-1 and TWIK-2 ("lower leg") – may be simultaneously occupied by a lipid plug in a bidentate manner. They further suggest that the R257A mutation alters the lipid plug's position, implicating this residue in lipid binding. While electrophysiological data indicate that certain mutants, including R257A, enhances TWIK-2 currents and is interpreted as disrupting lipid binding, they do not conclusively demonstrate this, and this is further not supported by structural work presented here.

We disagree with the reviewer's assessment regarding our structural work. We provide extensive structural evidence regarding the importance of the Arg257 position in stabilizing the position of the lipid plug and now support this point further with new molecular dynamics studies. We note that channels that show changes in the plug display both structural and biochemical properties that are not observed for the wild-type channel. As advised by Reviewer #3, have reframed the work as evidence for a working model of K_{2P} modulation to highlight this new proposal and need for further study.

Major Comments:

I have concerns that I would like to raise regarding modeling of the lipid within the cryo-EM density. Upon inspection of the two highest resolution structures of TWIK-2, resolved in a nanodisc (3.2 Å) and in detergent (3.7 Å), the density that I observe is broken or absent. The left panel is the density from the TWIK-2 nanodisc map (blue gray; yellow, modeled lipid) and the right is the cryo-EM density from the detergent map resolved (pink; orange, modeled lipid). Though it is possible that the lipid is not well ordered and therefore not well-resolved, I would be cautious in modeling a full lipid molecule into the pore. I would suggest that the authors model several acyl chains into the density that they are most confident about, similar to what was recently reported (see Khanra, Wang, Delgado, Long, PNAS, 2025). As an example of potential misinterpretation, inspection of the TWIK-2RM detergent 'plugged' map (3.9 Å) could lead one to interpret the pore occluding density as a lipid molecule, but oriented with its acyl chains pointing toward the bottom of the pore, rather than extending through the fenestrations (Figure 2). I would not personally conclude this, but I highlight this interpretation to illustrate that it is difficult to confidently model full lipids

into the pore within these maps, and make a point to bring this up since this is one of the main conclusions of the manuscript.

As the reviewer notes, acyl tails are dynamic, and when maps are viewed at higher thresholds, flexible regions frequently appear fragmented. When rendered at an appropriate threshold (~0.1), the vestibular density becomes continuous and adopts a geometry consistent with a lipid-like molecule rather than discrete acyl chains. (Fig. R1). We found this feature surprising and impossible to ignore (as noted in the response to Reviewer #1). This lipid-like density is observed in both nanodisc-reconstituted and detergent-purified samples of the wild-type TWIK2 protein. The properties of this density, as well as the biochemical properties of the channels, change when mutations are introduced that result in active channels. We now include MD studies that further support that such an entity can stably occupy the internal vestibule. As noted in the response to Reviewer #1, our interpretation of the lipid is not outside published examples. We have multiple lines of corroborating evidence regarding our interpretation of the density. Our studies provide a new working model for investigating how this class of K_{2P} channels are modulated. A suggested by Reviewer #3, we have softened the title to emphasize that this work does not represent the final answer but advances evidence for a novel working model for K_{2P} regulation.

Additional concerns that I would like to raise is the interpretation of the 'unplugged' and 'plugged' TWIK-2 structures presented by the authors. Upon comparing the 'unplugged' and 'plugged' maps, it is evident that the map sharpening B factor that was applied is drastically different, where the unplugged maps are sharpened to ~ -100 Å2 and the plugged maps are sharpened to ~ -230-250 Å2. Please note that this was mislabeled in Table 1 (value are reversed) and needs to be corrected. This method of comparing maps is inadequate, as high-resolution details are clearly more pronounced in the TWIK-2RM "plugged' map, as shown in Figure 3, and is even more pronounced when comparing the TWIK-2RM R257A maps. Also, since the 'unplugged' maps are poorly sharpened compared with the 'plugged' maps, I wonder if some density within the pore may have been overlooked (red circle in Figure 3). Alternatively, the density within the 'plugged' maps could be overinterpreted due to possible map "over sharpening". Overall, the maps should be compared using a similar map sharpening B factor to be able to make appropriate conclusions.

To address the concern regarding unequal sharpening, we reprocessed all "unplugged" and "plugged" maps using comparable global B-factors. Even with stronger sharpening, the $TWIK2_{RM}$ unplugged and $TWIK2_{RM}$R257A unplugged maps show an empty vestibule, aside from the granular low-level density (Fig. R4). We also note that such comparisons can be made by examining Figs. S2c, S13b, S17b, and S18b. The 'plugged' maps remain essentially unchanged when sharpened more using higher global B-factors, indicating that lipid-like densities were not a result of higher B-factor. We have updated sharpening values in Table 1 (210.4 Å² for the $TWIK2_{RM}$R257A "unplugged" map and 198.4 Å² for the $TWIK2_{RM}$ "unplugged" map). We are confident that the assignments of 'plugged' and 'unplugged' forms is correct.

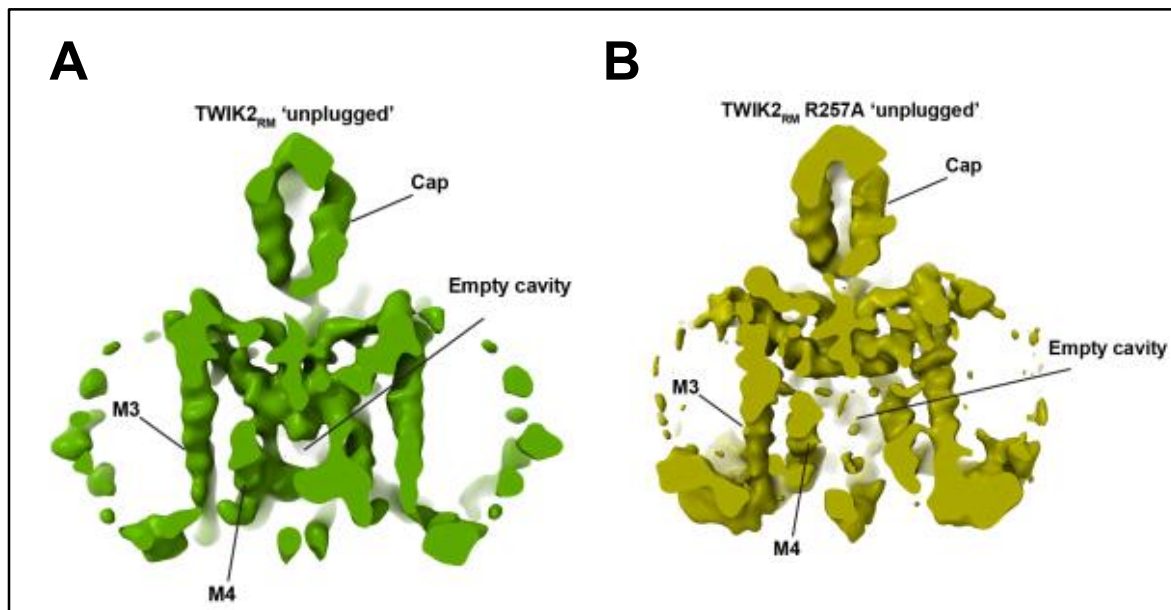

Fig. R4 Examplar densities for **A,** TWIK2RM 'unplugged' and **B**, TWIK2RM R257A 'unplugged' maps. Site of the empty cavity is indicated. the Select elements are labeled.

Regarding the density highlighted by the reviewer (red circle in Fig. 3 of their review), this feature remains faint and does not become a continuous or interpretable object under any reasonable sharpening conditions. We do not think it is prudent to provide an interpretation for this density.

Regarding the example from the reviewers about an unreasonable map interpretation (reviewer Fig. 2), we agree that one must be sensible when interpreting map features at any resolution. We note that the driving factors for our interpretation of the densities are both the density character and whether the potential occupant of the density makes physical chemical sense. One also needs corroborative evidence, such as what we provide through structural studies of mutant channels, functional tests, and MD simulations.

Another concern comes from the authors comparison of their lipid binding to what is reported in the cryo-EM structures of TWIK-1. In the TWIK-1 pH 5.5 cryo-EM map, there is clear electron density for acyl chains directly below the filter, which was what was modeled into the density by Turney et al., 2021, and is consistent with what was observed in the TWIK-1 crystal structure. I was surprised to see that, in this manuscript, the authors reinterpreted this structure and modeled in a full phospholipid bound into this map, despite very weak density in the region corresponding to the 'lower leg'. While it is conceivable that a lipid may be bound but not well resolved in the published TWIK-1 map, in my opinion, suggesting the presence of a bound lipid based on such limited density raises additional concerns for me. Furthermore, the interpretation of cryo-EM density from the TWIK-1 pH 7.5 map is difficult to believe since the map itself is of very poor quality, especially in the region of interest to the authors.

Because TWIK1 and TWIK2 are so similar, it is reasonable to ask if density corresponding to the lower leg might have been present in previously reported structures but not modeled. We find evidence for such as noted. The acyl-chain density reported by Turney *et al.* (2021) aligns with that seen in the TWIK1 crystal structure and with our assignment of the lipid upper tail. Examining lower thresholds in the maps shows that this density joins a density that occupies the lower tail cavity. Both channels have the same cavities that can accommodate the upper and lower tails. In response to the concerns of the reviewer, we have removed the following sentence from the manuscript:

> 'These observations suggest a revised interpretation of the origin of the alkyl chain density in the upper site of TWIK1 and indicate that it is from one of two legs of a lipid that blocks the pore as we observe in TWIK2.'

We note that this set of tunnels that can accommodate the lipid plug is not apparent in other K_{2P}s. Pointing out this shared architecture in the TWIK subfamily is important for advancing ideas about how such lipid plug molecules may affect function.

Overall, the mechanisms governing plug insertion, removal, or exchange remain speculative and not strongly supported by the cryo-EM maps. The authors suggest possible routes for lipid egress (e.g., conformational changes in M4/C-helix), but no direct evidence – structural, functional, or computational – is provided to support these hypotheses. As such, the precise gating mechanism and its physiological triggers remain incompletely defined. It is imperative that the authors conduct a more thorough examination of the proposed lipid plug gating mechanism. This would require MD simulations to assess lipid ingress/egress through fenestrations and correlate with plug stability and M4/C-helix motion and site-directed mutagenesis of M4/C-helix residues flanking the plug site to smaller (e.g., A) or bulkier (e.g., W) side chains and test for effects on plug stability and gating via cryo-EM or functional assays.

We disagree with the reviewer's assessment that the evidence for lipid-like blockade of the channel pore is insufficiently supported by our cryo-EM data. We present an extensive, systematic structural analysis that demonstrates the persistence of the lipid-like density in both nanodisc-reconstituted and detergent-solubilized TWIK2 channels. Structural investigation of constructs containing mutations that result in plasma membrane expression and functional

channels yield plugged and unplugged forms. This behavior not seen in the WT channel. The mutation that directs the channel to the plasma membrane also affects in the biochemical behavior of the channel by making the channel less stable during purification. This property is exacerbated by the R257A mutation. These changes in the physical properties of the channel are consistent with the observed changes in the occupancy of the plug lipid.

To investigate the role of the lipid plug further, we have now performed all-atom MD simulations to test lipid accommodation within the vestibule. The simulations demonstrate that the modeled lipid remains stably positioned throughout 1 µs of unrestrained sampling (Fig. 6). This finding reinforces the structural feasibility of the lipid plug. More importantly, simulations of R257A show a drop in the lipid plug position towards the position observed in the R257A structure (Fig. 6g-h) This R257A mutation increased channel current in electrophysiological assays and disrupted the corresponding lipid-like density in our maps. Together, these data indicate that Arg257 contributes to stabilizing the lipid plug and supports our interpretation.

Our data provide a new hypothesis for the control of TWIK2 function. Validating this hypothesis to the extent asked by the reviewer to prove a mechanism goes well beyond the scope of this initial study. In line with the request from Reviewer #3, we have changed the title to 'Evidence that a lipid plug controls K_{2P}6.1(TWIK2) function' and have now further highlighted the key questions it raises in the 'Discussion' section.

Reviewer #4

In this study Mondal and colleagues report a series of cryo-EM structures of the TWIK-2 (K2P6.1) channel, including wild-type and trafficking mutant channels in nano-discs and detergent, and an additional novel R257A mutant channel in detergent. These structures confirm some characteristic features of TWIK-2 first shown recently (and at higher overall resolution) in another study, as referenced [51], but also suggest a novel 'lipid plug' interaction, which is proposed as 'a mechanism to render the TWIK channels inactive'.

As the authors discuss, lipid-gating of (other) K2P channels has been proposed but not conclusively demonstrated, in part due to the challenging nature of testing these hypotheses. These new structures, and the re-evaluation of older TWIK-1 structures which suggest similar occupancy of both upper and lower fenestrations, raises an interesting potential new mechanism of channel regulation. However, ultimately, I feel that this study doesn't quite provide the killer experimental demonstration of this mechanism, unfortunately. I feel that this also means that the manuscript title is too assertive.

I suggest that the new data and potential mechanism are sufficiently interesting to warrant publication as a working model (with some concerns, as listed below) but this appears to be an opportunity to fully dissect what would be a novel K+ channel gating behavior. I hope the authors are allowed time to test proposed findings more thoroughly.

> We appreciate the sentiment that the title was too assertive. As a result, we have changed it to '***Evidence that*** **a lipid plug controls K_{2P}6.1(TWIK2) function'.** This rewording conveys that we are proposing a new working model, but that there are still open questions. We also changed the title of the model figure (Fig. 7) to '***Working*** **model of K_{2P}6.1(TWIK2) regulation'**

Major Concerns:

The density attributed to the lipid plug is novel in showing occupancy of the fenestrations consistent with a two-tailed native cell membrane phospholipid (and not a detergent molecule) which can block the permeation pathway. The identity of this lipid is not well determined, however. Lines 221-222 suggest contacts with the C-helix are 'consistent with phosphatidylethanolamine

head groups" but it is not clear how this is substantiated. Figures 5A,B, S18A, do not convincingly show this.

In response to this critique, we have removed this interpretation from the manuscript.

o Are there mutations in the C-helix which would be predicted to disrupt headgroup interactions? What are the effects of such mutations?

Because we do not see clear interactions between the C-helix and the lipids, we did not pursue this avenue of investigation. We tested deletion of the C-helix and truncation that removes the C-helix as well as the rest of the C-terminus of the channel. However, these changes yielded non-functional channels. We did not include these data in the revision. The open questions around the C-helix require an extensive investigation that is beyond the scope of our initial findings and are acknowledged as follows in the discussion (p. 12).

> Whether such conformational transformations constitute an autonomous mechanism driven by changes in lipid bilayer properties or requires the intervention of factors that facilitate lipid removal remains an open question in need of investigation.

• The mutagenesis experiments can be interpreted as consistent with the lipid-plug model but alone fail to convince that this exists as a functional mechanism. Previously, mutation of the equivalent of M135 in TWIK-1 (L146) has previously been implicated in altered hydrophobic gating. The M>D mutation which increased TWIK-2 currents could also impart this effect via reduced de-wetting. If a greater range of M135 substitutions fails to follow the relationship between hydrophobicity and TEVC currents shown for TWIK-1 in ref 49, this could disfavor a hydrophobic gating mechanism and support an alternative, such as the lipid plug mechanism.

In response to this point, we tested additional mutations that should alter the hydrophobicity of the central cavity (M135D/W/L/A/N). We find similar trends comparing the resultant functional changes with hydrophobicity using a well-established scale (Wimley, Cramer, and White, *Biochem* **35** 5109 1996) and sidechain volume (Fig. S14c). These data from deeper analysis of the M135 site cannot rule out either mechanism and are consistent with the idea that both plug removal and hydrophobic gating are in play. In fact, there is no reason that the two mechanisms need to be mutually exclusive, especially as mutations that increase hydrophobicity may have dual effects by increasing the residency of the lipid plug and by creating a hydrophobic barrier that needs to be dewetted for ions to pass.

We note that the strongest evidence for the plug model our structural observations as 1) the wild type channel is blocked by the lipid plug, 2) some fraction of the channels lose this block when we make a mutant that sends the channel to the plasma membrane, 3) some fraction of the channels also lose the plug with the R257A activating mutant, and show a different position of the blocking lipid.

We now include these additional mutants (Figs. 4b-c, S14a-b) and have altered the 'Results' as follows (p. 8-9) (new text in italics):

> Introduction of acidic residues at Met135 (M135D) and Arg257 (R257E) *increased basal activity with respect to* $TWIK2_{RM}$, as did replacement of Arg257 by alanine (R257A) (Fig. 4b-c, *S14b). By contrast, conservation of the positive charge in the central cavity (Arg257K) yielded channels that were not different from* $TWIK2_{RM.}$ *Because the Met135 site corresponds to a hydrophobic barrier site in TWIK1 that contributes to a dewetting mechanism[44], we investigated this site further. Mutations M135W/L/A/N/D yielded changes in the basal current of* $TWIK2_{RM}$ *that followed similar trends for both hydrophobicity[45] and sidechain volume[46] (Fig. S14c), suggesting that the dewetting mechanism may be important*

> *in TWIK2.* Comparison of ion selectivity by following the reversal potential as a function of external potassium showed no significant differences in the functional mutants from $TWIK2_{RM}$ (*Fig. S14d-e*). The increased activity observed for mutation at sites that interact with the blocking lipids is consistent with the idea that removal of the lipid plug is key to channel function *while the trends at Met135 are also consistent with dewetting[44], suggesting that both processes are important for TWIK2 function*. Importantly, the observation that introduction of a negative charge and truncation to alanine at the Arg257 site increases activity points to an important role for this position in TWIK2 function.

We had acknowledged the dewetting mechanism previously, but now have rewritten the 'Discussion' to elaborate on this point (p. 12) as follows (new text in italics):

> Wetting-dewetting of the narrow hydrophobic inner pore has been proposed as an important regulatory mechanism for TWIK channels that can be influenced by occupation of the lateral fenestration by lipid tails[13,49,50]. As such, there may also be some role for the competition between water and the lipid plug. *Importantly, the dewetting and plug mechanisms are not mutually exclusive and may both contribute to TWIK function.*

• Given the resolution of the plug density, the precise location of the atoms of any lipid appears difficult to discern. Thus, the suggestion that "The Arg257 sidechain, a residue conserved in vertebrate TWIK2 (Fig. S5), directly contacts the lipid glycerol backbone" is not well established. It's also not made clear why the R257A substitution would lead to the lower lipid binding pose.

We now include MD simulations that show that Arg257 makes varied types of contacts to the lipid glycerol backbone, indicating a dynamic interaction with the lipid plug. Further, simulations bearing the R257A mutation show a drop in the lipid position consistent with the structural observations. We think this is sufficient evidence to support the idea that Arg257 contacts the lipid plug. To clarify the link between Arg257 and the lipid pose we have rewritten the 'Results' (p. 9) as follows (new text in italics):

> Strikingly, in the $TWIK2_{RM}$ R257A-plugged form, the lipid plug resides ~5Å closer to the intracellular opening of the channel than in the other plugged TWIK2 structures (Figs. 5a-b, and S19a). *This change is consistent with the loss of stabilizing interactions with the Arg257 sidechain*. Because of this lower position, ~four upper site methylene groups and ~ten lower site methylene groups are pulled into the central cavity leaving a smaller portion of the upper and lower tails in their respective binding sites (Fig. 5a-b, and S19b).

• Strengthening this proposed model would require significant additional work, which could include MD simulations, mass spec analysis of bound lipids, or determining reconstituted channel activity in liposomes of defined composition (including altered PE levels) via the ACMA assay. Clearly, each are specialist methods, but data from any could elevate confidence in the proposed model.

In response to this suggestion, we now include extensive MD simulation analysis of wild-type, R257A, and R257K TWIK2. These studies show that the lipid position is stable and that its position and mobility are affected by the identity of the residue 257. Changes in the stability of the mutants complicate any mass spec approaches and as such, we did not pursue this direction due to the technical challenges.

• The lantibiotic, duramycin (Sigma #D3168) is reported to selectively bind to PE (PMID: 21573677). Is it possible to record TWIK2 currents in giant patches and apply this?

We did not test this hypothesis.

• The lipid plug conferring subcellular regulation is a neat idea, but what are the relative lipid compositions in the lysosome and plasma membrane? Are these compositions consistent with the RM mutation (and consequently increased plasma membrane trafficking) being likely to reduce PE binding as suggested from observation of unplugged RM mutant channels? Or binding by another possible candidate lipid?

Lysosomes have a different lipid composition from the plasma membrane. They are rich in sphingomyelin and have an uncommon negatively charged lipid bis(monoacylglyerophosphate). The situation is complex as the lipid composition changes from plasma membrane-like in early endosomes to more distinct compositions in more mature endo/lysosomes (new Refs. 55 and 56). How different lipid nanoenviroments affect membrane protein function is poorly understood as the effects are exceptionally complex involving changes in stiffness, compressibility, thickness, lipid distribution asymmetries, and other physical properties of the membrane. This is an area at the frontier of membrane protein biology and to our understanding, cannot be addressed with the precision requested by the reviewer. We now include references that highlight this complicated, and interesting issue and the diverse lipid compositions of various intracellular compartments (new Refs. 55 and 56). To clarify this point we have added the following to the ‘Discussion’ (p. 12). New text is in italics.

> Such changes in activity may be linked processes in which channel activation involves redistribution of TWIK2 channels from intracellular organelles to the plasma membranes as has been suggested during macrophage inflammasome activation[30,32-34]. *The membranes of different cellular compartments have diverse lipid compositions[55,56] and there is a growing appreciation of the importance of lipid nanoenviroments in controlling membrane protein function[55]. These factors raise the possibility that changes in local lipid nanoenvironments have a role in setting the proportion of plugged versus unplugged forms.* Together, our observations suggest the hypothesis that TWIK family activation is a ‘right place, right time’ process in which the channels remain plugged until they arrive at the cellular destination where their activity is required.

Other comments:

• The flipped-out conformation of Y111 is consistent with the recent structures from the Long group. The inactivation shown in S14 in these mutations Is interesting. Is this stabilized by elevated K+ (it seems the authors should have this data as high K+ recordings were made as shown in S14d)?

We now note that the observed inactivation in the Y111 mutants are potassium sensitive and is suppressed in elevated external potassium (20 mM is sufficient to eliminate this property). This sensitivity to external potassium is consistent with the function of the K2P channel SF C-type gates. We now mention this on p. 9 and show high potassium exemplars, and data showing the difference in inactivation properties in the new figures S15 f-g. We also include analysis of a new mutant, Y111D that changes this selectivity filter position to the most common residue found at this site in potassium channels. Interestingly this mutant is very activated and shows no inactivation, further supporting the role of the SF gate in TWIK2 function and K2Ps in general. We also refer to these results in the ‘Discussion’ on p. 13.

• As noted, the orientation of this residue has been associated with pH gating in TWIK1 and in TASK channels. Does the Y>H mutation impart pH gating to TWIK2?

We tested this hypothesis and found a pH response that was similar to the TWIK2. Hence, there is no gain of function with the Y111H mutation. We now note this on p. 9 and in new Figure S15b-d.

• The step-traces for M135D, R257A, and R257E mutations in Fig S14 appear to show that these mutations affect the voltage-activation of the channels, in that there is a significant increase in outwards currents at the -80 mV holding potential from these mutations compared with TWIK2RM. Was this consistent across recordings and can averaged data from these step protocols be included in this figure? This would be an interesting biophysical consequence, which is not discussed.

Thanks for encouraging us to mention this. It is described as follows on p. 8.

> Introduction of acidic residues at Met135 (M135D) and Arg257 (R257E) *increased basal activity with respect to TWIK2$_{RM}$*, as did replacement of Arg257 by alanine (R257A) (Fig. 4b-c, *S14b*).

• The use of 'leg' for the lipid acyl chains was a little confusing, is 'tail' not more standard?

Changed 'leg' to 'tail' as suggested (see also response to Reviewer #1)

• The x axis on S14b,d is presumably off by a value of 1. Where log10 [K+, mM] = 3 should be 2, consistent with a reversal potential of ~0mV. Can the authors confirm?

Corrected.

• Suggest to remove 'a' in: Structures of a previously characterized K2P6.1 (TWIK2), a mutant that increases the plasma membrane localization and activity, yield plugged and unplugged forms that highlight the importance of lipid removal for channel function.

Changed as suggested (p. 4)

• Suggest to add 'at' in: under conditions associated with inactivation of the channel C-type SF gate highlight the importance of structural changes [AT] this site for K2P function.

Changed as suggested (p. 6)

• Figure 3 legend includes 'd' twice, second one should be 'e'

Corrected

• Figure 5 legend includes 'd' twice, first one should be 'c'

Corrected

Revision 2

We thank the reviewers for their critical and constructive feedback on our work. We outline our responses to each of their points below. Reviewer's comments are in black. Our responses are in blue text.

Reviewer 1:

The revised manuscript appropriately moderates several of its original claims in response to reviewer critiques.

We thank the reviewer for this positive feedback and additional comments on the revised manuscript.

Previous structural studies have reported density within the TWIK channel cavity, and the present study similarly identifies comparable density that the authors interpret by modeling two lipid molecules. While this density should not be ignored, as noted by myself and Reviewer 3 in the initial review, additional experimental validation is required to substantiate both lipid occupancy and identity. At present, I remain unconvinced that cryo-EM data alone provide sufficient evidence to confidently assign these features as bound lipids, or indeed to definitively distinguish them from other possible molecular occupants. Considerable interpretive weight is placed on the density maps, and alternative explanations cannot be excluded. While I recognize the technical challenges associated with obtaining orthogonal validation, the current interpretation risks overstating what can be concluded from structural data alone. The Discussion should more explicitly acknowledge this limitation and emphasize that the presence, identity, and functional relevance of lipids within the channel cavity remain open questions in the field.

The molecular dynamics (MD) simulation section would benefit from further clarification. As currently described, simulations were initiated using structures containing the two modeled lipids within the cavity, leading to the conclusion that the simulations show stable lipid plug positions. This outcome is somewhat expected given the initial conditions and raises additional questions regarding how such lipids would exit the pore. Simulations initiated from lipid-free "unplugged" structures could provide complementary insight into whether lipids can spontaneously access the cavity. While such analyses may reasonably fall beyond the scope of the current manuscript, acknowledging this limitation would strengthen the interpretation.

We added the following (also in response to comments from Reviewer 6) to the 'Discussion' to address this point (p. 13):

> Simulations of the channel and Arg257 mutants that enhance function could provide additional insight into the role of dewetting and potential electrostatic barrier resulting from Arg257, as well as possible mechanisms for lipid ingress and egress.

The authors also report reduced biochemical stability for several channel mutants. However, decreased stability may complicate interpretation of the functional and structural results. In their response, the authors indicate that altered biochemical stability precluded additional experiments

on these variants. This limitation should be more explicitly discussed, as instability of the mutant channels may introduce ambiguity in attributing observed effects specifically to lipid interactions.

We agree that the identity of the lipid remains an open question. However, based on the structural data it is clear that the channel vestibule is blocked by two-tailed species that is most consistent with lipids and does not resemble the detergents used to purify the protein. Hence, the most parsimonious interpretation remains that these entities are lipids.

Following the suggestion from Reviewer 6, we calculated the lipid plug density based our MD simulations. The resulting maps (Fig. S21d-l) show densities that match what we see experimentally. Notably, at lower threshold (representing low resolution) the lipid plug appears to be an envelope matching our Cryo-EM densities, whereas at higher threshold the only visible parts are the far and mid segments of the tails of lipids in the lateral and vestibular cavities of the channel, respectively. This further analysis supports our assignment of the plug.

The discussion has been modified acknowledging these points (p. 13):

> Although our data show that the channel can exist in both plugged and unplugged forms, the identity of the lipids and how the lipid plug moves in and out is unclear. Corroborating the structural data, MD simulations show that the lipid plug is stable in the channel vestibule. The presence of the plug is incompatible with ion movement through the central cavity (Fig. 3h). Hence, its removal from the cavity is essential to yield a functional channel. How this can happen is not obvious.

$TWIK2_{RM}$, which shows two forms, is less stable than the WT. R257A, which also shows two forms was more unstable than $TWIK2_{RM}$. These issues are clearly mentioned (p. 8 & 9). As our observations are clearly reported, we did not elaborate further on the changes in channel stability.

Reviewer 2 & 3:

We appreciate the authors' efforts to further support their structural interpretation of lipid molecules occluding the pore and thank them for clarifying the density in their response to the reviewers. As the authors note, limitations in resolution and conformational heterogeneity are common for lipids in cryo-EM maps, with clear differences in density, for example, between their TWIK2 WT nanodisc and detergent maps. Although the modeled orientation of the lipid(s) is therefore uncertain, and we would still urge caution in model building, it is true that this does not alter the overall interpretation of the authors' proposed pore-plugging model. We also agree that the observation that the mutations destabilize the protein, potentially due to loss of the lipid ligand(s), is qualitative but a reasonable and helpful addition to the manuscript.

We appreciate the agreement on these points and thank the reviewers for their assessment of the revised manuscript.

The addition of all-atom MD simulations further strengthens the study, demonstrating that a modeled phospholipid can remain stably positioned within the cavity and that Arg257 contributes to its stabilization. However, in our original critique, we stated that mechanistic support would require, "MD simulations to assess lipid ingress/egress through fenestrations and correlate with plug stability and M4/C-helix motion and site-directed mutagenesis of M4/C-helix residues flanking the plug site to smaller (e.g., A) or bulkier (e.g., W) side chains and test for effects on plug stability and gating via cryo-EM or functional assays." While we appreciate the effort to perform MD simulations in support of plug stability, the other requested analyses were not addressed. It would be helpful if the authors could clarify why these were not pursued, as they are central to developing a mechanistic interpretation of lipid plug gating.

Overall, the current manuscript establishes a correlation between lipid density, residue interactions, and functional effects, but does not yet define the molecular pathway governing plug insertion, removal, or exchange. Thus, while the structural observations are intriguing and supported by MD simulations and structure–function analysis, the proposed gating model requires additional mechanistic support. In principle, such evidence would come from further computational sampling of lipid transit pathways and/or targeted mutagenesis of the proposed ingress/egress interface, as suggested previously.

If the authors are unable to perform these additional experiments, then the manuscript will need to be reorganized to deemphasize the lipid plug gating mechanism, as this remains a working model. Although the authors have already taken steps in this direction, the current phrasing still reads stronger than the available evidence supports.

We think there is a misunderstanding regarding mechanism. We are not advancing a model of 'gating' in the traditional sense (i.e. short term responses to stimuli, similar to what has been suggested by others) but rather are proposing a model in which the location of the channel is linked to the presence of the plug. For this reason, we have not focused on defining how the lipid gets in or out as there are a multitude of factors that could influence this process, including possibilities that involve interaction of the channel with other cellular components. This is very difficult to establish mechanistically. Moreover, to our knowledge none of the proposed lipid gating mechanisms have been proven for any K_{2P} and, as noted in the 'Introduction' (p. 3), remain controversial. The 'how' is not essential to the core message of the manuscript, which advances evidence for a regulatory working model for TWIK2. Indeed, we never used the term 'lipid plug gating mechanism' for this reason. However, it is clear that there is some confusion over this point. To address this issue we have now included the following in the discussion:

> We note that our proposed model (Fig. 7) is not a 'gating mechanism' for moment to moment control of channel function but outlines a regulatory pathway that can explain the two states that we observe.

To soften the tone further, we have rewritten the last sentence in the 'Introduction' as follows:

> The proposal that the lipid plug forms a physical barrier that prevents ion passage and that this plug can be destabilized by mutations that enhance channel function suggests a working model in which the function of the TWIK subfamily depends on lipid plug removal.

We think that our changes make it clear to the reader that this is a working model that needs further investigation.

Reviewer 5:

Please note that the reviewer's expertise is in MD simulations. Thus, this review will focus on the rigour within MD simulation methodology and the interpretation of the results. The MD simulations section of this manuscript seems to have been added in a very rushed manner, with one major flaw. This needs serious consideration before moving forward to acceptance.

We thank the reviewer evaluating our manuscript. We address the concerns raised as follows:

Major-point:

1. Figure 6g. It is stated that "The R257A change results in higher mobility of the lipid in the lipid head moiety (Fig. 6g)" under "Arg257 makes dynamic interactions with the lipid plug". The data, however, IS THE OTHER WAY ROUND. The RMSD of the head is LOWERED, suggesting a more stable binding. Unless the author has mislabelled the data, the conclusion does not hold together. The author also didn't state how the RMSD is calculated. The caption of the figure stated "RMSD plot of the lipid head relative to the starting position defined by the TWIK2:ND structure". Starting position of WHAT? The CA protein, the lipid headgroup - every atom? heavy atoms?. These matter massively in the value of the RMSD.

We apologize for the confusion on Fig. 6g. The two plots represented the behavior of the same lipid, but relative to two different references. One showed the behavior of the lipid relative to the position of the modeled lipid in the TWIK2-WT structure. The other showed behavior relative to the reference position of the 'dropped' lipid in the R257A structure. Hence, as we noted, during the simulation of the R257A system, we see that the lipid moves toward the lower position and consequently has a lower RMSD to the reference lipid in the R257A experimental structure.

All RMSDs, and now RMSFs in this manuscript have been calculated using heavy atoms. We now clarify this in the figure legends of Fig. 6 and S20.

2. The authors stated that the MD simulations were conducted for 1 us, in 3 independent conditions. WT, R257A and R257K. If I understood that the figure is labelled wrongly, the data is indeed

convincing on the importance of R257 as a key contacting residue. However, the conclusion is drawn from just 1 repeat of the MD simulations. I would request that the author perform at least 3 replicas for each condition to ensure that the POPC is indeed stable in the binding pocket.

We agree that further replicates were needed and have addressed this concern by performing triplicates of 1μS simulation for each. The updated RMSD plots (Figure S20) now reflect the averages across three replicates and suggest similar behavior as in the prior revision.

3. If the label were right, then please explain why having an alanine in the binding site is more stable than the arginine or lysine.

We apologize for the labeling confusion. The alanine mutation makes the lipid head more mobile. This is point is shown in Fig. 6.

Minor points

1. The figure legend shows a running average and a shaded average with no caption to explain what each of them means. Is it a running average? Average from 3 repeats? This needs contextualisation.

The figure has been modified and now represents a running average of RMSD from triplicates of each simulation. Shading indicated the SEM. These points are now clarified in the Figure legends of Fig. 6 and Fig. S20.

2. The H-bonding plot could be collapsed as a histogram, with the x-axis being the number of H-bonds, and the y-axis being the fractions of frames with H-bond form. With this, together with 3 repeats, you can statistically compare the tendency of H-bond formation between WT and the mutant.

Thanks for this suggestion. We now represent the H-bonding as histograms in Fig. 6d and e.

3. Caption Figure 6e, "Exemplar plot of Arg257 bridging involving coordination of phosphates from two lipids," makes no sense when the graph is plotting the number of bridging interactions. You mean the number of salt bridges between the phosphate and the guanidinium group? Then why two lipids? This is very unclear.

The lipid plug comprises a pair of lipids. The hydrogen bond histogram in Fig. 6e represents occurrences in which one arginine from a single channel subunit bridges the two lipids of the plug. We show an exemplar in Fig. 6f in which one of the two arginines (Arg257a) makes such an interaction. We clarified this point in the legend as follows:

> **f,** Simulation snapshot showing an exemplar bridging hydrogen bond made by Arg257a.

4. Again, the caption "h, Comparison of R257A lipid from simulation with the positions observed in TWIK2 and R257A.and h, TWIK2 R257K. Inset shows atom group." Why are there two h. You mean h and i? And you meant the same thing as g? And if you are going to say the same thing as g - why bother having 2 separate plots? You can have a single plot with 3 lines. If you have 3 repeats each, you can collapse all the data into a single histogram.

We have corrected the figure legend to h and i. Figure 6g and i show the lipid head behavior for the R257A and R257K mutants, respectively.

5. Figure 6h is just lipids floating in vacuo with no context from the protein structure. Why? It is bizarre to communicate those points without the side chain around it to bring the context together.

This figure shows an exemplar of the behavior plotted in 6g. The aim is to show that the lipid in the R257A simulation moves toward the dropped lipid position seen in the R257A structure and away from the position seen in the TWIK2 wild type structure. The protein is not shown to provide a simplified view. Legend is rewritten as follows:

> **h,** Exemplar comparing positions of the R257A lipid from simulation (grey) with the lipid positions observed in the TWIK2 (bright orange) and R257A (magenta) experimental structures. Channel is not shown.

Reviewer 6:

The manuscript is largely convincing and uncovers an important regulation mechanism of TWIK channels. We mostly comment on MD simulations.

The lipid plug hypothesis is plausible. However, one cannot rule out that the channel might not conduct due to dehydration of the cavity or due to a reduction of an electrostatic energy barrier, without the need for the lipid to penetrate. In that case, lipid entry could be a consequence rather than the cause. For example, the R257A mutation removes a positive electrostatic barrier along the permeation pathway, which could explain the larger currents in these mutants without invoking lipid blockage, since potassium ions are no longer repelled by the arginine. Ideally, to bring this point home, one could try simulations of the channel in the absence of the lipid, and report metrics such as water and ion density in the cavity in both cases. Given that this is not critical I won't push for these simulations in the current manuscript, but these points should be mentioned in the discussion.

We thank the reviewer for the positive evaluation of the manuscript. To address the point about dewetting and the potential barrier made by Arg257, we have included the following to the 'Discussion' (p. 13):

> Simulations of the channel and Arg257 mutants that enhance function could provide additional insight into the role of dewetting and potential electrostatic barrier resulting from Arg257, as well as possible mechanisms for lipid ingress and egress.

RMSD can be a degenerate metric. For the purpose of showing binding poses, it is better to perform clustering using RMSD as the metric and show the most populated cluster poses in the simulation, which in principle should correspond to the poses seen in the density, since both should, in principle, represent an energy minimum. Right now, the comparison shown in Fig6h seems arbitrary in terms of the lipid pose picked up from MD.

We do not think that the simulation has reached a global minimum regarding the lipid position in the R257A structure (as evident in Fig. 6g). We show an exemplar pose in Fig. 6h to illustrate the differences plotted in Fig. 6g. As we do not claim that the simulation pose represents a true minimum for the R257A structure, we have not included the clustering analysis. The main point is to show that the lipid position is dependent on the identity of residue 257. Attempts to connect the simulation with the final observed positions in the R257A structures would likely require much longer simulation times. To clarify this point, we added the following to the description of the simulations in the 'Results' section (p. 10):

> The movement of the lipid towards the cytoplasmic side of the channel was not completed in the timescale of the 1μs simulations, suggesting that longer times are required to reach the position observed experimentally.

In the same spirit, we would recommend to calculate the average lipid density from the simulation and compare it with the cryo-EM density. This would be a qualitative way to check that the lipid seen in the structure is actually plausible under simulation conditions, and vice versa, as shown by Aponte-Santamaria et al., PNAS 2012.

We thank the reviewer for this suggestion. We calculated the average lipid density from 1 μs production simulation by aligning the trajectories on the protein backbone and averaging the POPC plug occupancy over all frames. The resulting simulated density map shows a clear, continuous density for the bidentate lipid plug that closely matches the cryo-EM density observed in the experimental maps (Fig. S21d-l). This agreement validates that the lipid pose identified in the cryo-EM structure is plausible and stable under physiological bilayer conditions. Notably, under higher thresholds, the density for the head group disappears, leaving the density in the lipid tail binding sites, matching the behavior seen in the EM maps.

We also include the following description in the 'Results' section (p. 10-11):

> The lipid plug density in the TWIK2 Cryo-EM maps has poorer local resolution than the surrounding protein regions. To investigate the link between this property and lipid flexibility,

> we calculated time-averaged density maps[51] from the TWIK2, R257A, and R257K 1μs all-atom MD simulations. The resultant maps (Fig. S21d-l) show density that matches our experimental observations. Notably, at stringent density thresholds, the lipid head density is lost, whereas the density in the two lipid tail binding cavities remains. These results qualitatively recapitulate the behavior of the experimental densities, with clear enrichment of the upper and lower lipid tail positions in the respective fenestrations observed in the structures. Taken together, the simulations support both the stability of the lipid plug and emphasize the important role of the Arg257 position in lipid plug stabilization.

And the following in the 'Discussion (p. 12):

> Thus, previous observations of density in the upper tail site may represent incomplete density for similar lipid plug structures arising from lipid dynamics, an interpretation that is in line with our density analysis from MD simulations (Fig. S21d-l).

To better assess the dynamic nature of the bound lipid, RMSF analysis would be better suited (or could complement) the segment-based RMSD - the fact that RMSD is large with respect to the starting position does not mean that the lipid shows a dynamical behaviour throughout the simulation

We now include RMSFs plots of the various lipid segments (Fig. 21a-c).

Residue M135 is important for the current response of this channel and also seems to interact with the lipid, but no structural analysis is carried out of how this residue interacts with the lipid. Some more discussion could be provided on this.

We have performed functional studies mutating M135 to W/L/A/N/D to understand if this residue plays critical role in dewetting mechanism as was seen in TWIK1 (p. 8-9). Apart from showing that this residue interacts with the lipid (Fig. S8), we did not investigate this site further as our focus turned to role of Arg257, which stands out as a charged residue in the central cavity that can directly coordinate the lipid.

We thank the reviewers for their critical assessment of our work. Our responses are in blue text.

Reviewer #1

The authors have addressed my concerns and recommend for publication.

Thank you. There are no further points to address for this Reviewer.

Reviewer #2

I have no additional comments. We appreciate the authors' efforts to address our concerns and revise the wording.

Thank you. There are no further points to address for this Reviewer.

Reviewer #5

We thank the author for the revision of the manuscript. I am happy with the EPhys and the structural work which is clearly the expertise of the authors. However, there are still some error on MD simulation to my concern as follow.

1. In all of your RMSD calculation, it is said that it is fitted to the initial structure. If that so, t = 0 should have 0 RMSD. Why is it not beginning with 0? At the end of your simulation (1000 ns), the RMSD decreases - which is rather odd unless it goes back to the fitted structure. What you plot are more likely corresponded to the RMSD fitted to the end state?

We apologize for the confusion on this point. It was correctly described in the methods section but not in the Figure 6 legend. We now clarify that the RMSDs are relative to the cryo-EM coordinates for the lipid in the initial cryo-EM structures (either the WT or the R257A structures). We added the following to the Fig. 6 legend.

> Lipid RMSDs are relative to the indicated reference cryo-EM structures.

2. This figure legend
d, Exemplar plot Arg257-phosphate hydrogen bonds made byone sidechain.

e, Exemplar plot of Arg257 bridging involving coordination of phosphates fromtwo lipids by forming bidentate hydrogen bonds.

This makes no sense. You are trying to say that R257 forms less hydrogen bond with 2 lipids comparing to 1 lipid? or e only counts the case when the R257 forms two hydrogen bond simultaneously with 1 H-bond to two lipids? Can you please rephrase your figure legend, or show clearly in your figure what are you actually calculating?

'd' shows the hydrogen bond frequences for one Arg257 with one lipid. 'e' shows the frequency of bridging hydrogen bonds in which one Arg257 interacts with two lipids (the second case mentioned by the reviewer and represented by the R257a exemplar in panel 'f'). We have rewritten the figure legend as follows:

d, Plot of Arg257-phosphate hydrogen bonds made by one sidechain.

e, Plot of Arg257 bridging hydrogen bonds involving one Arg257 and two lipids.

3. Again - the mutation to A makes RMSD value lowered (orange). This suggests stabilisation of the lipid, not that the lipid is more dynamics. If this is calculated with respect to the starting structure, then clearly R to A mutation stabilised the lipid. Is this another mislabelling? If your observation is indeed true, you need to change your metric of evaluating headgroup dynamics within the binding pocket.

The lipid is more dynamic as it moves from the upper position (i.e. WT pose) to a pose that nears the position observed in the R257A cryo-EM structure. The blue curve is calculated versus the wild-type position (as noted by the label WT ref). The orange curve is calculated versus the R257A position (as noted by the label R257Aref). So yes, the lipid appears to be on its way to a new stable position from where it started (WT position → R257A position). To make this clearer, we added the following to the figure legend (new text in italics)

g, RMSD plots of the lipid head heavy atoms relative to their starting position defined by the TWIK2:ND structure (WT ref) (blue) or the position of the lipid in the TWIK2 R257A structure (R257A ref) (orange) (n =3). *These indicate that the lipid moves from the WT position towards the position seen in the R257A structure.*

Reviewer #6

All my points were sufficiently addressed

Thank you. There are no further points to address for this Reviewer.

A lipid plug affects K_{2P}6.1 (TWIK-2) function

Abhisek Mondal, Sangeeta Niranjan, and Daniel L. Minor Jr.

In this study, the authors report a gating mechanism for the two-pore domain potassium (K_{2P}) channel family member TWIK-2 (K_{2P}6.1) involving lipid-mediated pore block. Using high-resolution cryo-EM structures, the authors propose that this lipid plug serves as a gating mechanism that functionally silences TWIK channels. The study addresses a longstanding question in the K_{2P} field regarding the role of lipid densities seen in channel fenestrations. Based on the cryo-EM structures of TWIK-2 presented in this manuscript, comparisons with previously published TWIK-1 cryo-EM structures, and structure-function guided mutagenesis near putative lipid-binding sites, the authors propose that two acyl chain binding sites observed in TWIK-2 - one conserved among K_{2P} channels ("upper leg") and another unique to TWIK-1 and TWIK-2 ("lower leg") – may be simultaneously occupied by a lipid plug in a bidentate manner. They further suggest that the R257A mutation alters the lipid plug's position, implicating this residue in lipid binding. While electrophysiological data indicate that certain mutants, including R257A, enhances TWIK-2 currents and is interpreted as disrupting lipid binding, they do not conclusively demonstrate this, and this is further not supported by structural work presented here.

Major Comments:

I have concerns that I would like to raise regarding modeling of the lipid within the cryo-EM density. Upon inspection of the two highest resolution structures of TWIK-2, resolved in a nanodisc (3.2 Å) and in detergent (3.7 Å), the density that I observe is broken or absent. The left panel is the density from the TWIK-2 nanodisc map (blue gray; yellow, modeled lipid) and the right is the cryo-EM density from the detergent map resolved (pink; orange, modeled lipid).

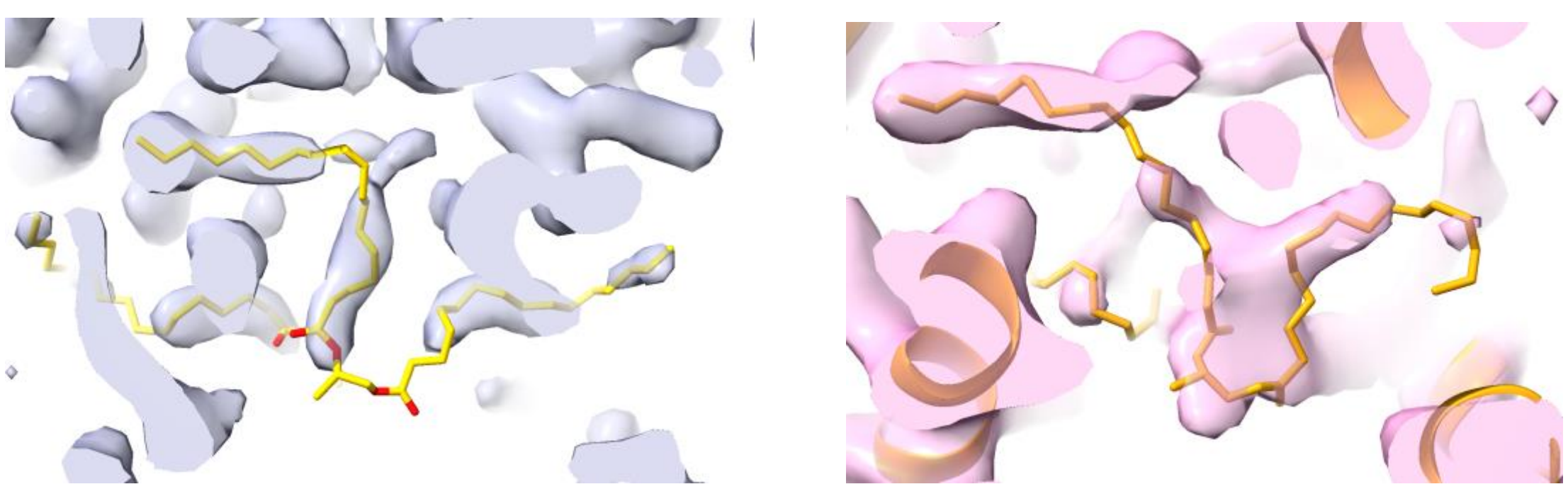

Figure 1: Modeled lipids (yellow, orange) into the density from the TWIK-2 nanodisc (blue gray) and detergent (pink) maps.

Though it is possible that the lipid is not well ordered and therefore not well-resolved, I would be cautious in modeling a full lipid molecule into the pore. I would suggest that the authors model several acyl chains into the density that they are most confident about, similar to what was

recently reported (see Khanra, Wang, Delgado, Long, PNAS, 2025). As an example of potential misinterpretation, inspection of the TWIK-2_{RM} detergent 'plugged' map (3.9 Å) could lead one to interpret the pore occluding density as a lipid molecule, but oriented with its acyl chains pointing toward the bottom of the pore, rather than extending through the fenestrations (Figure 2). I would not personally conclude this, but I highlight this interpretation to illustrate that it is difficult to confidently model full lipids into the pore within these maps, and make a point to bring this up since this is one of the main conclusions of the manuscript.

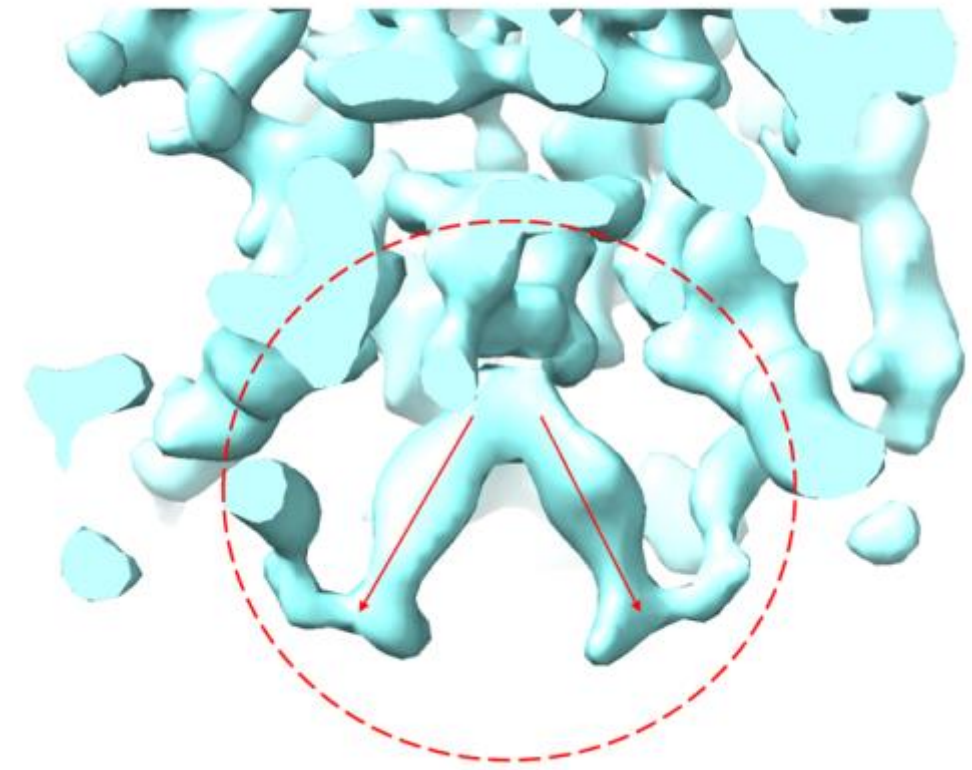

Figure 2: The TWIK-2 pore from TWIK-2_{RM} plugged cryo-EM map, with possible lipid circled and acyl chains labeled with arrows. This is in a markedly different orientation to the structures in Figure 1.

Additional concerns that I would like to raise is the interpretation of the 'unplugged' and 'plugged' TWIK-2 structures presented by the authors. Upon comparing the 'unplugged' and 'plugged' maps, it is evident that the map sharpening *B* factor that was applied is drastically different, where the unplugged maps are sharpened to ~ -100 Å^2 and the plugged maps are sharpened to ~ -230-250 Å^2. Please note that this was mislabeled in Table 1 (value are reversed) and needs to be corrected. This method of comparing maps is inadequate, as high-resolution details are clearly more pronounced in the TWIK-2_{RM} "plugged' map, as shown in Figure 3, and is even more pronounced when comparing the TWIK-2_{RM} R257A maps. Also, since the 'unplugged' maps are poorly sharpened compared with the 'plugged' maps, I wonder if some density within the pore may have been overlooked (red circle in Figure 3). Alternatively, the density within the 'plugged' maps could

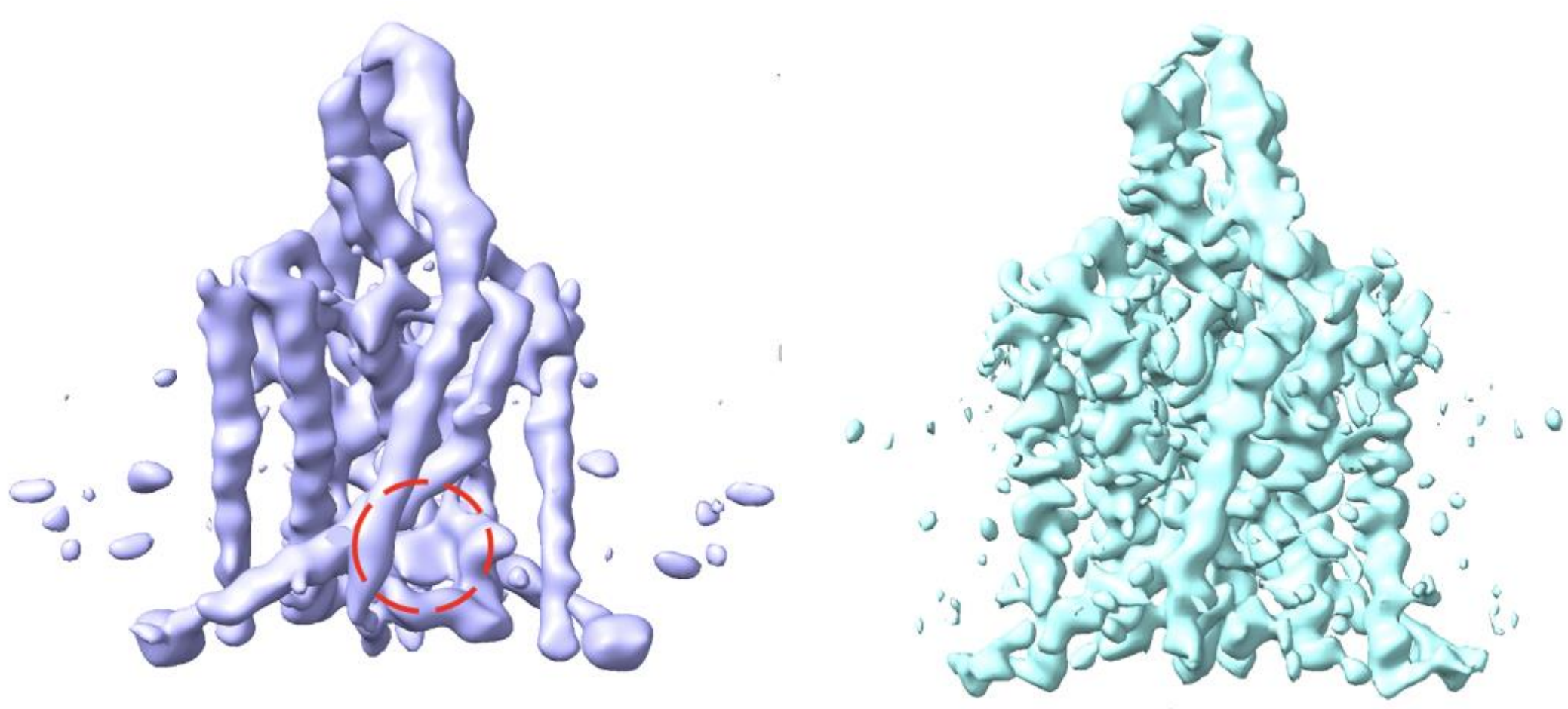

Figure 3: Cryo-EM density of the TWIK-2_{RM} unplugged (purple, left) and plugged (cyan, right) cryo-EM maps. Red circle indicated potential lipid density within the lower fenestration, though in this map, it does not appear to extend into the pore.

be overinterpreted due to possible map "over sharpening". Overall, the maps should be compared using a similar map sharpening *B* factor to be able to make appropriate conclusions.

Another concern comes from the authors comparison of their lipid binding to what is reported in the cryo-EM structures of TWIK-1. In the TWIK-1 pH 5.5 cryo-EM map, there is clear electron density for acyl chains directly below the filter, which was what was modeled into the density by Turney et al., 2021, and is consistent with what was observed in the TWIK-1 crystal structure. I was surprised to see that, in this manuscript, the authors reinterpreted this structure and modeled in a full phospholipid bound into this map, despite very weak density in the region corresponding to the 'lower leg'. While it is conceivable that a lipid may be bound but not well resolved in the published TWIK-1 map, in my opinion, suggesting the presence of a bound lipid based on such limited density raises additional concerns for me. Furthermore, the interpretation of cryo-EM density from the TWIK-1 pH 7.5 map is difficult to believe since the map itself is of very poor quality, especially in the region of interest to the authors.

Overall, the mechanisms governing plug insertion, removal, or exchange remain speculative and not strongly supported by the cryo-EM maps. The authors suggest possible routes for lipid egress (e.g., conformational changes in M4/C-helix), but no direct evidence – structural, functional, or computational – is provided to support these hypotheses. As such, the precise gating mechanism and its physiological triggers remain incompletely defined. It is imperative that the authors conduct a more thorough examination of the proposed lipid plug gating mechanism. This would require MD simulations to assess lipid ingress/egress through fenestrations and correlate with plug stability and M4/C-helix motion and site-directed mutagenesis of M4/C-helix residues flanking the plug site to smaller (e.g., A) or bulkier (e.g., W) side chains and test for effects on plug stability and gating via cryo-EM or functional assays.

Minor comments:

- Within the introduction, the labeling of K2P1.1 (TWIK1) and K2P6.1 (TWIK2) is included 13 times. Please remove additional references to TWIK1 and TWIK2.
- Figure 1e, I would suggest including a second panel (f) to separately show the up and down states of Tyr111 modeled into the density.
- 191-192 However, introduction of acidic residues at Met135 (M135D) and Arg257 (R257E) resulted **<u>'in'</u>** increased basal activity with respect to TWIK2RM, as did replacement of Arg257 by alanine (R257A).
- 200 down conformations prompted us to test **<u>'the'</u>** effect of replacing this residue with smaller, more commonly observed
- 223 The observation that ~~**the**~~ R257A affects lipid plug position indicates that this residue has a

- 299 Structures of_TWIK2 in lipid and detergent environments shows that this channel conforms to the overall
- 336 K_{2P}1.1, (no space)
- Figure 3g, is the model displayed of TWIK2 determined in the nanodisc (based on coloring), or is it intended to be the $TWIK2_{RM}$ 'unplugged' model based on the hole diagram?
- Figure S3b, the colors of the structures are too similar. Can the authors change the color of TWIK-1 displayed in this panel
- Figure S8 figure legend, it is out of order. "Sequences in 'b' and 'c' line should be before "**d,** View of K_{2P}1.1"
